# Development of Cellular Energy Metabolism During Differentiation of Human iPSCs into Cortical Neurons

**DOI:** 10.1007/s12035-025-05284-8

**Published:** 2025-11-13

**Authors:** Šárka Danačíková, Petr Pecina, Alena Pecinová, Jan Svoboda, David Vondrášek, Davide Alessandro Basello, Tomáš Čajka, Daniel Hadraba, Tomáš Mráček, Vladimír Kořínek, Jakub Otáhal

**Affiliations:** 1 Department of Pathophysiology, Second Faculty of Medicine, Charles University, Prague, Czech Republic; 2 Laboratory of Developmental Epileptology, Institute of Physiology of the Czech Academy of Sciences, Prague, Czech Republic; 3 Laboratory of Cell and Developmental Biology, Institute of Molecular Genetics of the Czech Academy of Sciences, Prague, Czech Republic; 4 Department of Physiology, Faculty of Science, Charles University, Prague, Czech Republic; 5 Laboratory of Bioenergetics, Institute of Physiology of the Czech Academy of Sciences, Prague, Czech Republic; 6 Laboratory of Biomathematics, Institute of Physiology of the Czech Academy of Sciences, Prague, Czech Republic; 7 Laboratory of Metabolomics, Institute of Physiology of the Czech Academy of Sciences, Prague, Czech Republic

**Keywords:** Human iPSCs, Neuronal differentiation, Cellular bioenergetics, Respirometry, Proteomics, Metabolic flux analysis

## Abstract

Neuronal differentiation requires extensive metabolic remodeling to support increased energetic and biosynthetic demands. Here, we present an integrated multi-omics and functional characterization of metabolic transitions during early differentiation of human induced pluripotent stem cells (iPSCs) into excitatory cortical neurons using doxycycline-inducible overexpression of neurogenin-2 (NGN2). We analyzed parental iPSCs and induced neurons (iNs) at days 7 and 14 of differentiation, integrating gene expression profiling, label-free quantitative proteomics, high-resolution respirometry, fluorescence lifetime imaging microscopy (FLIM), and ^13^C₆-glucose metabolic flux analysis. Our data reveal progressive metabolic remodeling associated with neuronal maturation, including enhanced oxidative phosphorylation, increased mitochondrial content, and respiratory capacity. Proteomic analyses showed upregulation of mitochondrial and antioxidant pathways, while FLIM indicated a progressive increase in enzyme-bound NAD(P)H, consistent with a shift toward oxidative metabolism. Notably, ^13^C₆-glucose tracing revealed delayed labeling of the intracellular pool of fully labeled glucose and tricarboxylic acid cycle metabolites, together with enhanced labeling of pentose phosphate pathway intermediates and glutathione in iNs, indicating a shift toward biosynthetic and antioxidant glucose utilization during differentiation. Despite this enhancement in mitochondrial function, differentiated neurons maintained glycolytic activity, suggesting metabolic flexibility. Our results define the first week of differentiation as a critical window of metabolic specialization and establish NGN2-iPSC-derived cortical neurons as a versatile and well-characterized model system for investigating bioenergetic remodeling during early human neurodevelopment. It provides a robust foundation for mechanistic insights and high-throughput evaluation of metabolic pathways relevant to human disease.

**Supplementary Information:**

The online version contains supplementary material available at 10.1007/s12035-025-05284-8.

## Introduction

Neuronal differentiation involves dynamic remodeling of the cellular architecture, culminating in neurite outgrowth, synapse formation, and establishment of functional networks. These changes are supported by fundamental metabolic reprogramming that enables neurons to meet increasing energy and biosynthetic demands. A hallmark of this transition is the shift from glycolytic metabolism, typical of proliferative progenitors, to oxidative phosphorylation (OXPHOS). This switch supports more effective adenosine triphosphate (ATP) production, mitochondrial biogenesis, maturation, regulation of neurotransmitter release, maintenance of redox homeostasis, and resting membrane potential [1, 2].

Beyond this global shift, aerobic glycolysis remains active in differentiating neurons and plays a crucial role in supporting localized energetic and biosynthetic demands [3, 4]. In the adult brain, aerobic glycolysis represents approximately 20% of total glucose utilization. During childhood, particularly at times of intense synaptogenesis and neuronal growth, this proportion rises to roughly threefold higher levels [5–9]. Glycolytic enzymes are enriched in axonal growth cones, where they supply local ATP to support the high-energy demands of cytoskeletal remodeling. This localized glycolysis compensates for the limited ATP supply from distally located mitochondria due to the limited diffusion capacity of ATP [10]. These observations highlight the need for spatial and temporal regulation of metabolic pathways during neurodevelopment.

A detailed characterization of metabolic regulation during early neuronal development is critical for understanding the basis of neurodevelopmental disorders, many of which are increasingly linked to impaired bioenergetics. Leigh syndrome, a rare and severe pediatric mitochondrial disease, is most commonly caused by mutations in complex I or IV of OXPHOS, although defects in complexes II and V have also been reported [11, 12]. Another mitochondrial disorder, the MELAS syndrome (mitochondrial encephalomyopathy, lactic acidosis, and stroke-like episodes), is usually caused by mutations in mitochondrial tRNA genes and is associated with impaired assembly and function of complexes I or IV [13, 14]. In epilepsy, emerging evidence suggests a complex interplay between metabolic dysregulation and epileptogenesis; however, it remains unclear whether metabolic impairment contributes to seizure susceptibility or results from ongoing epileptic activity [15–17]. Together, these examples underscore the importance of studying metabolic remodeling during human neurodevelopment and emphasize the need for reliable, neuron-specific model systems that can recapitulate early stages of differentiation and bioenergetic specialization. A detailed understanding of metabolic trajectories in healthy neurons is a necessary prerequisite for identifying how these processes are disrupted in neurodevelopmental disorders.

Human induced pluripotent stem cells (iPSCs) represent a versatile platform for investigating key questions in neurodevelopmental and neurodegenerative diseases, with broad potential for both basic and translational research. Using doxycycline (dox)-inducible overexpression of neurogenin-2 (*NGN2*), iPSCs can be rapidly and efficiently differentiated into excitatory cortical neurons within two weeks, expressing mature markers such as microtubule-associated protein 2 (MAP2) [18, 19]. These NGN2-induced neurons (iNs) exhibit functional properties such as synaptogenesis and electrophysiological activity and have been used to study neuronal maturation, connectivity, and cell subtype identity [20–22]. Multi-omics approaches have further characterized the molecular dynamics of NGN2-mediated differentiation, including transcriptional and proteomic changes associated with neuronal identity and cellular processes such as autophagy. Notably, several studies have reported increased expression of mitochondrial genes and proteins involved in OXPHOS, supporting the notion of mitochondrial remodeling during differentiation [23–25]. However, despite increasing recognition of the importance of metabolic remodeling for neuronal differentiation, the timing and functional characteristics of these changes in NGN2-induced neurons remain insufficiently characterized. In particular, it is not well established at which stages of the differentiation process key metabolic transitions occur and how they contribute to the acquisition of a mature neuronal phenotype. Unlike human brain tissue or co-culture systems, NGN2-induced neurons provide a homogeneous neuronal population, allowing direct assessment of metabolic remodeling to neurons themselves without confounding contributions from glial or vascular cells.

To address this gap, we applied an integrated multi-omics and functional approach, combining proteomics, high-resolution respirometry, fluorescence lifetime imaging microscopy (FLIM), and ^13^C_6_-glucose metabolic flux analysis, to characterize early metabolic remodeling of human iPSCs differentiating into cortical neurons. Our data identify the first week of differentiation as a critical window for bioenergetic specialization and redox adaptation, validating NGN2-induced neurons as a metabolically characterized and broadly applicable human model for investigating neurodevelopment and mitochondrial disease.

## Materials and Methods

### Cell Cultures and NGN2-Mediated Neuronal Differentiation

The human iPSCs with dox-induced expression of NGN2 were used as a model system for our experiments [19, 26]. The iPSCs were kindly provided by D. Bohačiaková (Faculty of Medicine, Masaryk University Brno, Czech Republic). The iPSCs were cultured in Essential 8 (E8) medium (Thermo Fisher Scientific), containing d-glucose (17.5 mM), l-glutamine (2.5 mM), and sodium pyruvate (0.5 mM) [27] on plates coated with vitronectin (VTN; Thermo Fisher Scientific) in feeder-free conditions. IPSCs were regularly passaged using ReLeSR™ (STEMCELL Technologies) and Dulbecco’s phosphate-buffered saline (DPBS; Thermo Fisher Scientific). The cortical neuron differentiation protocol was performed according to [19, 26]. On day 0, iPSC colonies were harvested using Accutase (Merck) to obtain a single-cell suspension. The cells were plated on VTN-coated plates and cultured in the neuronal induction medium (NIM): Dulbecco’s Modified Eagle’s Medium (DMEM)/F12 (21,331,020, Thermo Fisher Scientific), HEPES (15 mM), N2 supplement (Thermo Fisher Scientific), GlutaMAX™ (2 mM, Thermo Fisher Scientific), MEM Non-Essential Amino Acids (0.1 mM; Thermo Fisher Scientific), supplemented with dox (2 µg/ml), and ROCK inhibitor (RI; 10 µM; STEMCELL Technologies). The NIM medium contained d-glucose (17.5 mM), l-alanyl-l-glutamine (2 mM), and sodium pyruvate (0.5 mM). On days 1 and 2, the NIM medium was replaced with fresh NIM supplemented with dox only. On day 3, the cells were harvested with Accutase and plated on plates coated with poly-l-ornithine (Sigma-Aldrich) and laminin (Thermo Fisher Scientific) in cortical neuron medium (CM). The CM medium composition: BrainPhys™ Neuronal Medium (STEMCELL Technologies; cat 5790), containing d-glucose (2.5 mM), l-alanyl-l-glutamine (0.5 mM), and sodium pyruvate (0.5 mM) [28], B-27™ Supplement minus vitamin A (Thermo Fisher Scientific; cat 12,587,010), brain-derived neurotrophic factor (BDNF; 10 ng/ml; PeproTech), neurotrophin-3 (NT-3; 10 ng/ml, PeproTech), and laminin (1 µg/ml; Thermo Fisher Scientific). Half of the medium volume was changed every three days. The cells were analyzed on day 0 (iPSCs), day 7 (iNs_D7), and day 14 (iNs_D14).

### Cell Imaging

Cells were cultured in 35 mm glass-bottom dishes (Cellvis). Morphological images of iPSCs and iPSC-derived neurons were acquired using an inverted Leica DMI8 widefield microscope. Images were processed using Fiji (ImageJ) software [29].

### Reverse Transcription Quantitative Polymerase Chain Reaction (RT-qPCR)

Cell cultures were washed with DPBS and harvested using TRIzol™ Reagent (Thermo Fisher Scientific). Total RNA was isolated using the phenol–chloroform extraction method, followed by DNase I (1 U/µl; Thermo Fisher Scientific) treatment to remove residual genomic DNA. Complementary DNA (cDNA) synthesis was performed using RevertAid Reverse Transcriptase (Thermo Fisher Scientific). RT-qPCR was performed with three independent samples (biological replicates); four biological replicates were used to quantify the expression of heterogeneous nuclear ribonucleoprotein E1 (*hnRNP-E1*), and each sample was analyzed in technical triplicate. LightCycler® 480 SYBR Green I Master (Roche) was used as the detection dye, and the reactions were run in a LightCycler® 480 Instrument II (Roche). The comparative analysis involved normalization of cycle threshold (Ct) values to the reference gene TATA-box-binding protein (*TBP*) [30]. Primer sequences are provided in Online Resource 2—Table 1. Data are presented as mean ± SD, with individual points representing the mean of technical replicates from each biological sample. Statistical significance was determined using one-way ANOVA followed by Tukey’s multiple comparison test. For *REX1*, a Kruskal–Wallis test with Dunn’s multiple comparisons was applied.

### Methylation Assay

Genomic DNA was extracted using the AllPrep DNA/RNA Mini kit (Qiagen). For bisulfite modification, genomic DNA (0.5 µg) from iPSCs was treated using the Imprint DNA Modification Kit (Sigma-Aldrich). The bisulfite-treated DNA was amplified using KAPA 2G Robust Hot Start ReadyMix (KapaBiosystems) with the following primers: hFNANOG: 5′-GAGTTAAAGAGTTTTGTTTTTAAAAATTAT-3′; hRNANOG: 5′-GAAAGATATGATAAATTATTAGATTTGGGA-3′; hFOCT4: 5′-GGATGTTATTAAGATGAAGATAGTTGG-3′; hROCT4: 5′-AATAGATTTTGAAGGGGAGTTTAGG-3′. The PCR products were purified and cloned into the pGEM-T-Easy vector (Promega). Nine plasmids with cloned amplified DNA fragments were analyzed by sequencing. The methylation status at CpG dinucleotides was assessed using the online quantification tool for methylation analysis (QUMA) (Laboratory for Mammalian Epigenetic Studies, Center for Developmental Biology, RIKEN). Coordinates of CpG sites are based on the GRCh38 reference genome (Ensembl annotation version).

### Proteomics

Label-free quantitative mass spectrometry analysis (LFQ-MS) was performed with cell pellets from three independent differentiations for iPSCs and iNs_D7 and four independent differentiations for iNs_D14. Briefly, cellular pellets (100 µg of protein) were processed according to the protocol for in-solution trypsin digestion [31]. About 500 ng of desalted peptide digests was separated on a 25 cm C18 column using a 1.5-h gradient elution and analyzed in a DIA mode in the Orbitrap Exploris 480 mass spectrometer (Thermo Fisher Scientific) equipped with a FAIMS unit. The resulting raw files were processed in Spectronaut (v. 18.7, Biognosys) using directDIA mode and human proteome UP000005640_9606.fasta (UniProt release 2024_01). Downstream data processing was performed using Perseus software (version 2.0.11.0) [32]. Enrichment analysis was performed using the GSEA software version 4.3.3 (Broad Institute) [33, 34], using gene sets from the Molecular Signatures Database (MSigDB), including the Hallmark, Kyoto Encyclopedia of Genes and Genomes (KEGG), Gene Ontology Biological Process (GOBP) collections, and the cultured human stem cell classification Mueller Plurinet [35–38]. LFQ-MS data are available via ProteomeXchange with identifier PXD064787 [39, 40].

### Western Blotting

SDS-PAGE Western blotting analysis was performed essentially as described in Čunátová et al. (2024) [41]. Weighed cellular pellets were solubilized in ice-cold RIPA buffer (150 mM NaCl, 1% Nonidet NP-40, 1% sodium deoxycholate, 0.1% SDS, 50 mM Tris, pH 8.0) supplemented with a phosphatase inhibitor cocktail (1:200, Sigma-Aldrich P5726) and Benzonase® nuclease (1:1000, Merck 70,664). Samples were centrifuged (10,000 × g, 15 min), and the protein concentration of the supernatant was determined by BCA assay (Sigma-Aldrich B9643). Samples were mixed with SLB buffer (sample lysis buffer, final concentrations; 2% (v/v) 2-mercaptoethanol, 4% (w/v) SDS, 50 mM Tris (pH 7.0), 10% (v/v) glycerol, 0.02% Coomassie Brilliant Blue R-250) and incubated at 65 °C for 10 min. Protein aliquots (30 µg) were separated in 12% polyacrylamide gels using the Mini-PROTEAN III apparatus (Bio-Rad, USA). Proteins were transferred to polyvinylidene difluoride (PVDF) membranes (Immobilon FL 0,45 µm, Merck) by semi-dry electroblotting (0.8 mA/cm^2^, 1 h) using a Transblot SD apparatus (Bio-Rad). For immunodetection, the primary and secondary antibodies, together with their validation, are listed in Online Resource 2—Table 2. Signals were detected by Odyssey imager (LI-COR Biosciences) and quantified by Image Lab software (Bio-Rad). For Western blotting, three independent neuronal differentiations (three biological replicates) were analyzed for each condition, with each biological replicate performed in technical triplicate. Statistical analysis was performed using one-way ANOVA followed by Tukey’s multiple comparisons test. Error bars represent ± SD. Original, unprocessed Western blot image files and corresponding quantification reports are available in Online Resource 4.

### Oxygen Consumption

IPSCs and iPSC-derived neurons were prepared as cell suspensions to measure oxygen consumption. For iPSCs, cells were washed with DPBS and dissociated using Accutase. For iPSC-derived neurons, cells were washed with DPBS and incubated in an enzymatic mix of Accutase and papain (1:1; Worthington Biochemical) following the manufacturer’s protocol. After enzymatic dissociation, cells were centrifuged and resuspended in the assay medium: DMEM (Merck, cat: D5030) supplemented to final concentrations of 10 mM glucose, 1 mM pyruvate, 2 mM glutamine, and 0.20% bovine serum albumin (BSA). Oxygen consumption was measured immediately after cell dissociation using the Oxygraph-2 k instrument (Oroboros Instruments GmbH, Innsbruck, Austria). Following calibration, cells were loaded into the chambers at a density of 1.0–1.5 × 10⁶ cells per chamber, the chambers were closed airtight, and cells were allowed to equilibrate. During the measurement, oxygen concentration dropped from an initial 185 nmol/ml to approximately 120 nmol/ml. After recording routine oxygen consumption rate, sequential injections of metabolic inhibitors and uncouplers were performed: 0.25 µM oligomycin (OLIG), 0.75–3.5 µM carbonylcyanide-p-trifluoromethoxyphenylhydrazone (FCCP), and 0.25 µM rotenone (ROT), to record respiratory rates representing proton leak-driven respiration (LEAK), maximal capacity of the electron-transport system (ETS), and non-mitochondrial residual oxygen consumption (ROX) (Fig. 4a). Oxygen consumption rates were analyzed using DatLab 5 software (Oroboros Instruments GmbH). Data were normalized to cell count and expressed as pmol O₂/(s* Mill). Statistical analysis was performed using one-way ANOVA followed by Tukey’s multiple comparisons test. For LEAK, routine control ratio, and respiratory reserve capacity, a Kruskal–Wallis test with Dunn’s multiple comparisons was applied. Error bars represent ± SD.

### Fluorescence Lifetime Imaging Microscopy (FLIM)

Cells (iPSCs, iNs_D8, and iNs_D14-16) were cultured in 35 mm glass-bottom dishes (Cellvis) and measured in their respective culture medium at 37 °C. The temperature and atmosphere mixture of 5% CO₂ with compressed air was controlled by an Okolab Bold Line top stage incubator with an H301-K-Frame chamber (Okolab S.r.l., Italy).

FLIM measurements were performed using a Leica SP8 WLL MP microscope (Leica Microsystems GmbH, Germany) equipped with a 63x/1.2 water objective. Fluorescence was generated with a two-photon excitation source, an infrared pulsed laser (Chameleon Discovery TPC, Coherent Inc., USA). The laser power was set approximately to 10 mW at sample plane (measured with PM3 sensor + FieldMax II, Coherent Inc., USA). A potassium dihydrogen phosphate (KDP) crystal was utilized as a reference for measuring the instrument response function (IRF). An excitation laser wavelength of 750 nm was used for nicotinamide adenine dinucleotide (phosphate) (NAD(P)H), while a coumarin sample excited by a laser at 940 nm served as a calibration control. Fluorescence was detected by a HyD-RLD non-descanned detector, using a 472/30 nm bandpass filter to capture the emission signal for NAD(P)H, and 525/50 nm for coumarin 6 (98% dissolved in ethanol). Fluorescence decays were measured by a time-correlated single photon counting (TCSPC) system (Simple-Tau-150-D1, Becker and Hickl GmbH, Germany). Images were acquired with 256 × 256 pixels, 0.48 × 0.48 μm pixel size, at 100 Hz scanning speed, 12 bit, with a 1.5 × zoom. Measurements were performed in unidirectional mode, with a 5-min acquisition time [42, 43].

FLIM data were analyzed using the software SPCImage NG v9.0 (Becker and Hickl) for phasor plot analysis. A binning factor of 2 was applied to improve the signal-to-noise ratio. The measured IRF was used for the analysis. Further processing, including cell segmentation, was performed in MATLAB software (MathWorks), focusing on measuring intensity-weighted fluorescence lifetimes (τ) and calculating the free/bound NAD(P)H ratio [44]. Normality of the data was tested by Q-Q plot and the Shapiro–Wilk test. Statistical analysis was performed using one-way ANOVA followed by Tukey’s multiple comparisons test. Error bars represent ± SD.

### Glucose Tracing

IPSCs and iPSC-derived neurons iNs_D14 were cultured in triplicates on 60 mm dishes in their respective growth media (see the part “Cell Cultures and NGN2-Mediated Neuronal Differentiation” for details). The cultured medium was replaced with unlabeled DMEM medium, supplemented to final concentrations of HEPES (10 mM), sodium bicarbonate (30 mM), sodium pyruvate (1 mM), glutamine (1 mM), and glucose (5 mM) for 2 h to equilibrate the metabolite levels in both cell types. Then, the medium was replaced with tracing medium (same as unlabeled DMEM medium, except that unlabeled glucose was replaced with stable isotope-labeled U-^13^C_6_-glucose (5 mM; Cambridge Isotope Laboratories)), and cells were collected before the addition of tracing medium and subsequently after 5, 15, 60, and/or 360 min of incubation time. Cells were washed with DPBS, harvested (on ice), centrifuged (200 × g, 5 min, 4 °C), and the resulting cell pellets were immediately frozen in a dry ice/ethanol bath. The samples were stored at − 80 °C.

### LC–MS-Based Metabolomics

Cells were processed using the LIMeX workflow, and polar metabolites were extracted with a biphasic solvent system composed of cold methanol, methyl tert-butyl ether, and 10% methanol, followed by liquid chromatography-mass spectrometry (LC–MS) analysis [45]. Specifically, one aliquot from the bottom aqueous phase was evaporated and resuspended in an acetonitrile/water (4:1, v/v) mixture containing CUDA and Val-Tyr-Val as internal standards. This sample was analyzed using the hydrophilic interaction chromatography (HILIC) metabolomics platform in positive (for detection of glutathione) or negative (for detection of glucose) electrospray ionization mode. Another aliquot of the bottom phase was mixed with an acetonitrile/isopropanol (1:1, v/v) mixture, evaporated, then resuspended in 5% methanol with 0.2% formic acid, again including CUDA and Val-Tyr-Val as internal standards. This sample was analyzed using the reversed-phase liquid chromatography (RPLC) platform in negative electrospray ionization mode (for detection of ribose-5-phosphate, citrate, succinate, fumarate, and lactate).

LC–MS analysis was conducted using a Vanquish UHPLC system (Thermo Fisher Scientific) coupled to an Orbitrap Exploris 480 mass spectrometer (Thermo Fisher Scientific). Detailed chromatographic and detection parameters are provided elsewhere [46]. For ^13^C_6_-metabolic flux analysis, MS1 data were acquired at a mass resolving power of 180,000 FWHM. Data were processed using MRMPROBS software to provide peak heights of ^13^C-isotopologues based on the total number of carbon atoms per analyte. Isotopic labeling was corrected for natural ^13^C-abundance using IsoCor software [47]. Statistical analysis was performed using an unpaired *t*-test. Data represent mean ± SD from three independent experiments.

## Results

### Establishment and Characterization of an In Vitro Cortical Neuron Model

**Fig. 1:**
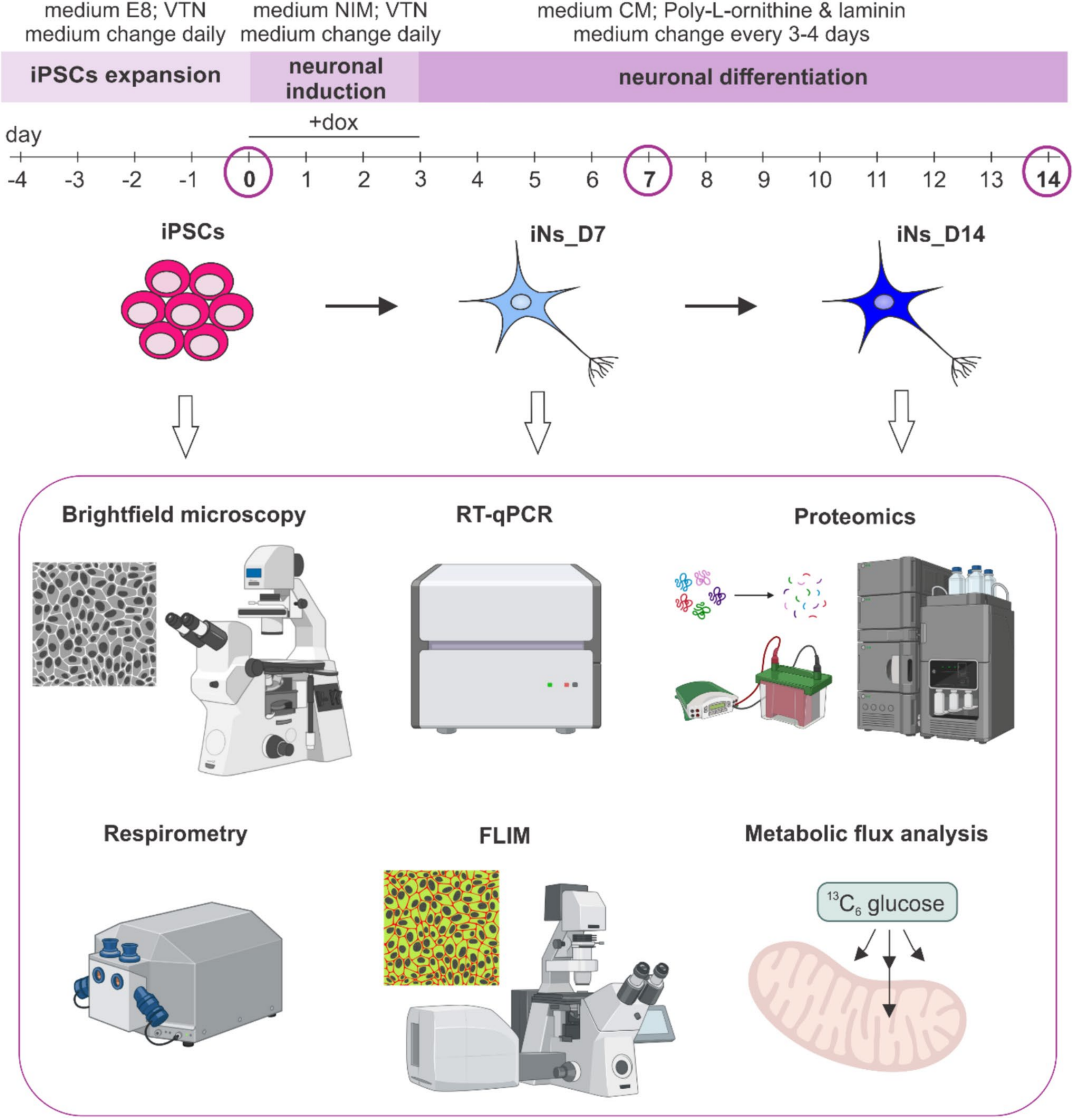
Schematic overview of the differentiation protocol from human induced pluripotent stem cells (iPSCs) into cortical neurons and methods used for cell characterization. The 2–3 week procedure is divided into three phases: iPSCs expansion, neuronal induction, and neuronal differentiation. During the expansion phase, iPSCs are cultured in Essential 8 (E8) medium on vitronectin (VTN)-coated plates. Neuronal induction is initiated by addition of doxycycline (dox) to the neuronal induction medium (NIM), with cells maintained on VTN-coated plates. During the maturation phase, NGN2-induced neurons (iNs) are cultured in cortical medium (CM) on poly-l-ornithine and laminin-coated plates. Key collection time points are indicated: undifferentiated iPSCs (day 0), iNs at day 7 (iNs_D7), and day 14 (iNs_D14). Undifferentiated iPSCs, iNs_D7, and iNs_D14 were analyzed using the following methods: morphological analysis using bright-field microscopy (Brightfield microscopy), evaluation of pluripotent and neural markers by reverse transcription quantitative polymerase chain reaction (RT-qPCR), proteomic profiling by liquid chromatography-mass spectrometry and Western blotting (Proteomics), functional assessment using high-resolution respirometry (respirometry), fluorescence lifetime imaging microscopy (FLIM), and metabolic flux analysis with ^13^C_6_-labeled glucose (metabolic flux analysis). Created in BioRender

**Fig. 2:**
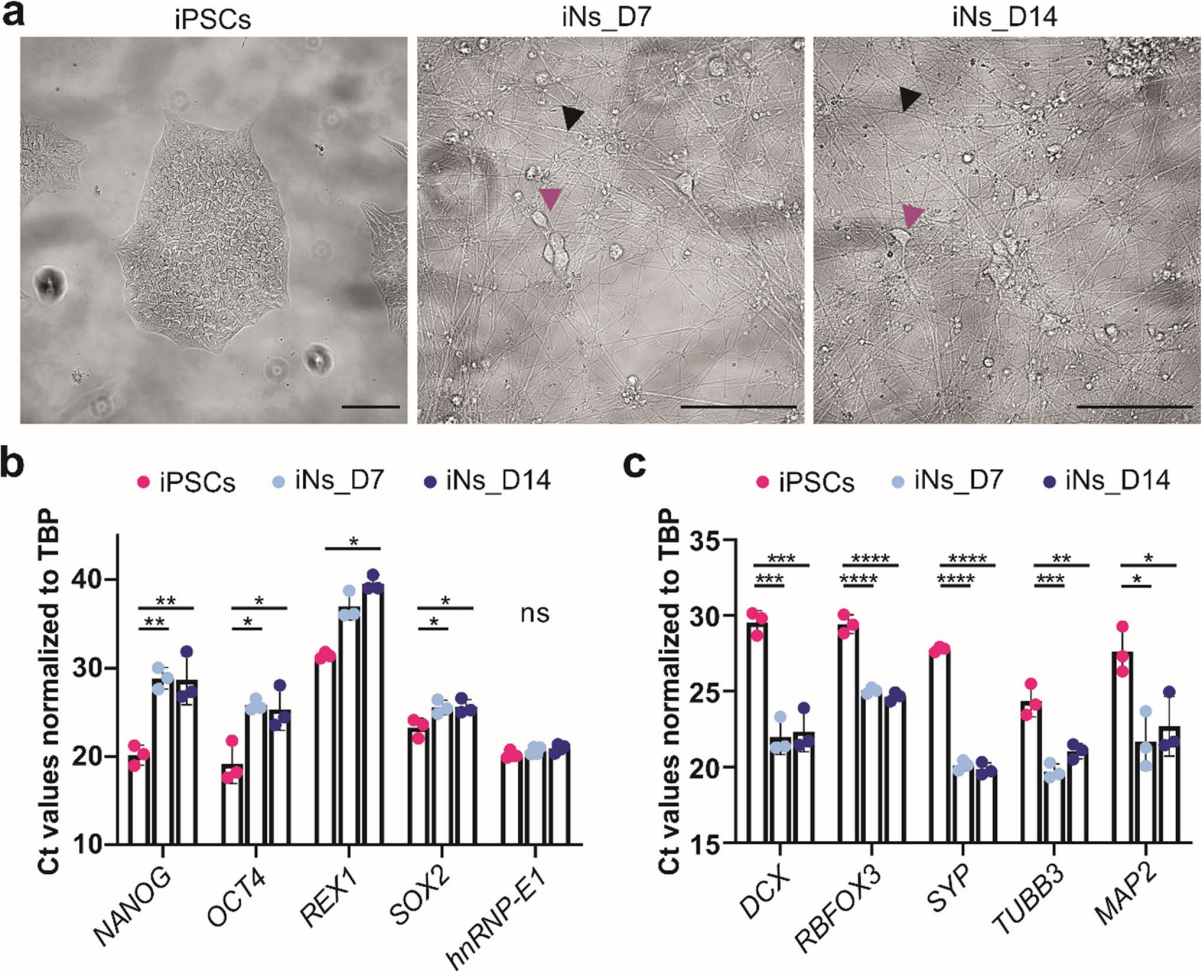
Morphological changes indicative of a neuronal phenotype decreased expression of pluripotency genes and upregulation of neuronal markers during differentiation of iPSCs into iNs. **a** Representative brightfield images showing the morphology of iPSCs and iNs_D7 and iNs_D14. Purple arrow: neural cell body; black arrow: neurite. Scale bar 100 µm. **b** RT-qPCR analysis of pluripotency markers (*NANOG*, *OCT4*, *REX1*), pluripotency and neural stem cell marker (*SOX2*), and heterogeneous nuclear ribonucleoprotein E1 (*hnRNP-E1*) in iPSCs, iNs_D7, and iNs_D14. **c** RT-qPCR analysis of neural markers (*DCX*, *RBFOX3*, *SYP*, *TUBB3*, *MAP2*). Cycle threshold (Ct) values were normalized to the TATA-box-binding protein (*TBP*) gene expression level. Lower Ct values correspond to higher gene expression, and higher Ct values indicate lower gene expression. Each data point represents the mean of technical replicates from one biological replicate, normalized to *TBP*. Data are presented as mean ± SD (*n* = 4 for *hnRNP-E1*; *n* = 3 for all other markers). Statistical significance was determined using one-way ANOVA followed by Tukey’s multiple comparison test. For *REX1*, a Kruskal–Wallis test with Dunn’s multiple comparisons was applied. (*) *p* ≤ 0.05; (**) *p* ≤ 0.01; (***) *p* ≤ 0.001; (****) *p* ≤ 0.0001. DCX, doublecortin; MAP2, microtubule-associated protein 2; NANOG, Nanog homeobox; OCT4, octamer-binding transcription factor 4; RBFOX3, RNA binding fox-1 homolog 3; REX1, reduced expression 1 (zinc finger protein 42); SOX2, SRY-box transcription factor 2; SYP, synaptophysin; TUBB3, tubulin beta 3 class III

To establish a platform for proteomic and metabolic analyses, we differentiated human iPSCs into excitatory cortical neurons using a protocol adapted from Fernandopulle et al. (2018) [19, 26]. Cells were collected for analysis at day 0 (iPSCs), day 7 (iNs_D7), and day 14 (iNs_D14). The differentiation protocol and methods for cell characterization are summarized in Fig. 1. During the expansion phase, iPSCs formed round compact colonies. Upon induction, cells acquired neuronal morphology, including neurite outgrowth, indicating successful differentiation (Fig. 2a).

At the molecular level, differentiation was confirmed by changes in the expression of pluripotency and neuronal markers assessed by RT-qPCR. Expression of pluripotency markers Nanog homeobox (*NANOG*), octamer-binding transcription factor 4 (*OCT4*), reduced expression 1 (*REX1*), and SRY-box transcription factor 2 (*SOX2*) significantly decreased in both iNs_D7 and iNs_D14 compared to iPSCs (Fig. 2b), consistent with the loss of pluripotent identity. In parallel, early and mature neuronal markers, including doublecortin (*DCX*), RNA binding fox-1 homolog 3 (*RBFOX3*), synaptophysin (*SYP*), tubulin beta 3 class III (*TUBB3*), and *MAP2*, were upregulated (Fig. 2c), confirming the acquisition of the neuronal phenotype. All Ct values were normalized to the housekeeping gene *TBP*, which remained stable throughout the differentiation. Additionally, hnRNP-E1 was used as an additional normalization control for Western blot analysis (Fig. 2b).

As a part of routine quality control during iPSC maintenance, the methylation status of *NANOG* and *OCT4* gene promoters was assessed, confirming transcriptionally active states via detection of unmethylated CpG islands (Online Resource 1—Fig. S1) [48]. These results together demonstrate efficient neuronal differentiation, validated across morphological and transcriptional levels.

### The Transition to a Neuronal Phenotype Involves the Activation of Oxidative Metabolism and Mitochondrial Biogenesis

**Fig. 3:**
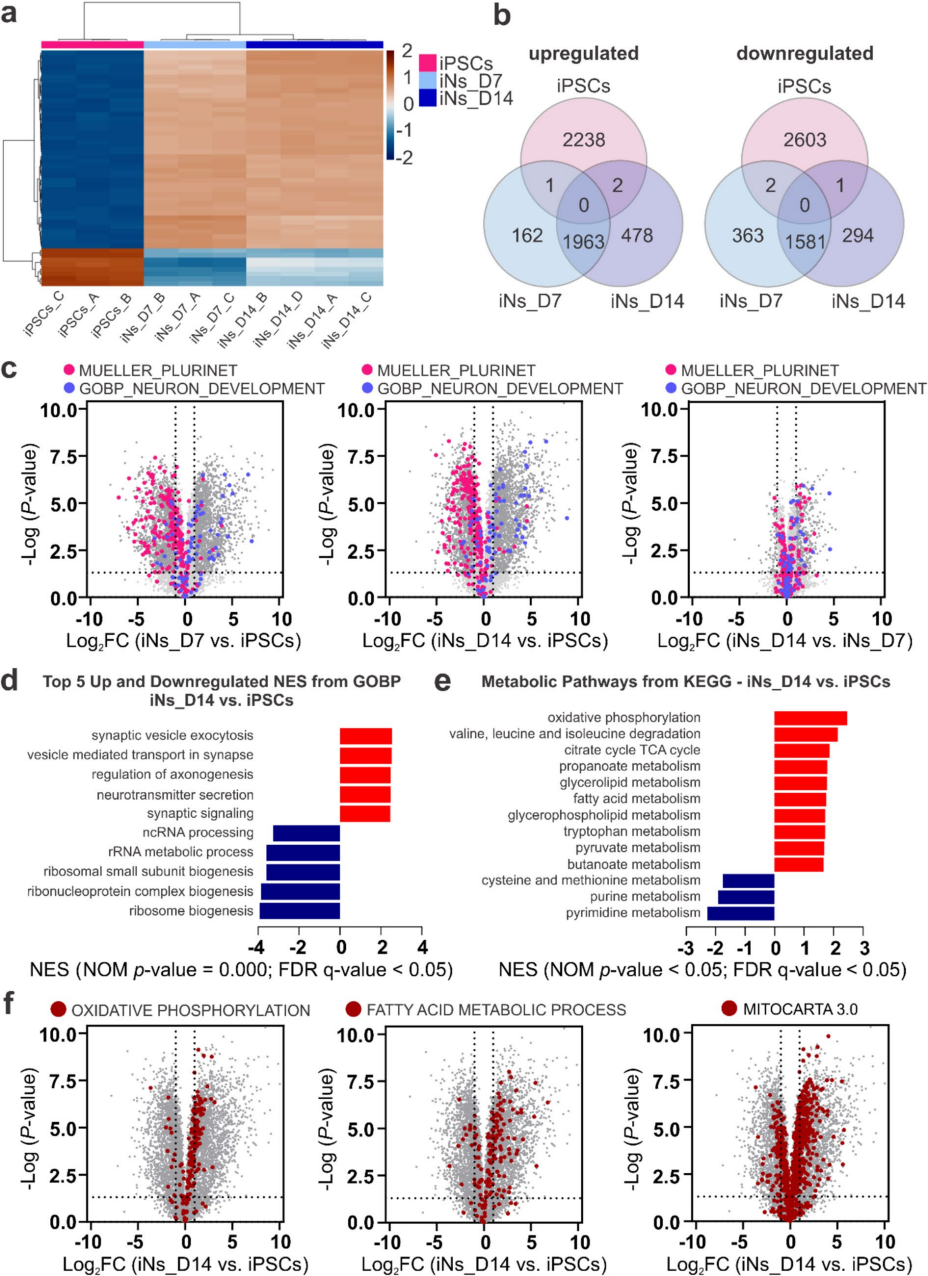
Dynamic changes in protein abundance, neuronal identity, and metabolic pathways during differentiation of human iPSCs into iNs. Human iPSCs exhibit distinct changes during differentiation, with the most pronounced transition occurring between iPSCs and day 7 cells (iNs_D7), with further but less extensive changes observed between iNs_D7 and day 14 cells (iNs_D14). Proteomic profiling was performed using label-free quantification mass spectrometry (LFQ-MS) on independent differentiations: *n* = 3 (iPSCs), *n* = 3 (iNs_D7), and *n* = 4 (iNs_D14). **a** Heatmap of the 50 most differentially expressed proteins with the highest fold change (log_2_FC). Hierarchical clustering shows different expression profiles in iPSCs, iNs_D7, and iNs_D14. Red means higher expression, and blue means lower expression. The proteins annotated by gene names, based on their corresponding UniProt/Swiss-Prot identifiers, are listed in Online Resource 3. **b** Venn diagrams with upregulated and downregulated proteins (log_2_FC ≥  ± 1; *p* < 0.05). The largest overlap is seen between iNs_D7 and iNs_D14. **c** Volcano plots show differing protein expressions (*x*-axis: log_2_FC; *y*-axis: − log_10_ *p*-value). Significantly upregulated and downregulated proteins are shown in dark gray. Pink highlights proteins related to pluripotency (Mueller Plurinet), and blue highlights proteins involved in neuron development (Gene Ontology Biological Process (GOBP) Neuron Development). Comparisons: iNs_D7 vs. iPSCs; iNs_D14 vs. iPSCs; iNs_D14 vs. iNs_D7. **d** GOBP enrichment analysis in iNs_D14 vs. iPSCs. Top five upregulated (red) and downregulated (blue) metabolic pathways based on normalized enrichment scores (NES). Significance: NOM *p*-value = 0.000; FDR *q*-value < 0.05. **e** Enrichment of metabolic pathways from the Kyoto Encyclopedia of Genes and Genomes (KEGG) database in iNs_D14 vs. iPSCs. Shown are the ten most up- and downregulated metabolic pathways (NOM *p*-value < 0.05; FDR *q*-value < 0.05). **f** Volcano plots show the differentially expressed proteins in iNs_D14 compared to iPSCs. All proteins are shown in dark gray. Proteins from the Hallmark Oxidative Phosphorylation, GOBP Fatty Acid Metabolic Process, and MitoCarta 3.0 sets are shown in dark red

Early neuronal differentiation is associated with extensive proteomic and metabolic remodeling. To characterize these changes, we performed LFQ-MS on iPSCs, iNs_D7, and iNs_D14. Hierarchical clustering of the top 50 differentially expressed proteins separated iPSCs from both neuronal populations, with a substantial proportion of proteins linked to neurodevelopmental processes (Fig. 3a, Online Resource 3). In agreement with these observations, proteomic analysis revealed a decrease in the abundance of pluripotency-associated proteins (NANOG, OCT4, SOX2) and increased levels of neuronal markers (DCX, RBFOX3, SYP, TUBB3, MAP2) during neuronal differentiation (Online Resource 1—Fig. S5), consistent with transcriptional changes detected by RT-qPCR (Fig. 2b–c). Subsequently, a comparison of significantly up- and downregulated proteins (log_2_FC ≥  ± 1; *p* < 0.05) revealed a substantial overlap between iNs_D7 and iNs_D14, including 1963 upregulated and 1581 downregulated proteins compared to iPSCs (Fig. 3b). These findings suggest that the most pronounced proteomic changes occur within the first week of differentiation.

Enrichment analysis, primarily based on the GOBP database, revealed biological processes driving neuronal differentiation. Significant enrichment of pathways involved in neuronal development, including axonogenesis, synaptic function, and signaling, was observed in both iNs_D7 and iNs_D14 compared to iPSCs. In contrast, downregulated pathways were primarily associated with RNA processing and ribosome biogenesis (Fig. 3d, Online Resource 1—Fig. S2a-b), consistent with a reduction in cell proliferation. Volcano plots further illustrated a shift from pluripotency-associated proteins (Mueller Plurinet) to neuron-specific proteins (GOBP Neuron Development) during differentiation (Fig. 3c).

Given the critical role of metabolism in neuronal development, we next assessed the metabolic pathway regulation. KEGG pathway enrichment analysis revealed the most significant upregulation of the OXPHOS pathway and downregulation of purine and pyrimidine metabolism in iNs_D7 and iNs_D14 compared to iPSCs (Fig. 3e, Online Resource 1—Fig. S2c), further supporting reduced proliferative activity and a shift toward a postmitotic neuronal phenotype. In the direct comparison of iNs_D14 to iNs_D7, we observed the most substantial upregulation in glycerophospholipid metabolism, consistent with ongoing membrane remodeling during neuronal maturation, while glycolysis and gluconeogenesis pathways were downregulated (Online Resource 1—Fig. S2d).

Integration of enrichment analyses and volcano plots further confirmed metabolic reprogramming, with upregulation of OXPHOS (Hallmark OXPHOS), fatty acid metabolism (GOBP Fatty Acid Metabolic Process), and mitochondrial proteins (MitoCarta 3.0). In contrast, glycolytic pathways remained largely unchanged (Hallmark Glycolysis). Consistent with a shift from a proliferative to a post-mitotic neuronal state, we also observed a reduction in cell cycle-associated proteins (KEGG cell cycle) (Fig. 3f, Online Resource 1—Fig. S2e; Fig. S3a-c).

Together, our findings demonstrate that the first week of neuronal differentiation represents a critical period of proteomic and metabolic reprogramming. This includes suppression of pluripotency markers, acquisition of neuronal identity, and reduced proliferative activity. Differentiating neurons also exhibit enhanced glycerophospholipid metabolism, consistent with the extensive membrane remodeling required for neurite outgrowth and synaptogenesis. Moreover, neuronal maturation is marked by activation of mitochondrial biogenesis and upregulation of oxidative metabolic pathways, including OXPHOS and fatty acid oxidation.

### Mitochondrial Respiratory Capacity Increases During Neuronal Differentiation

**Fig. 4:**
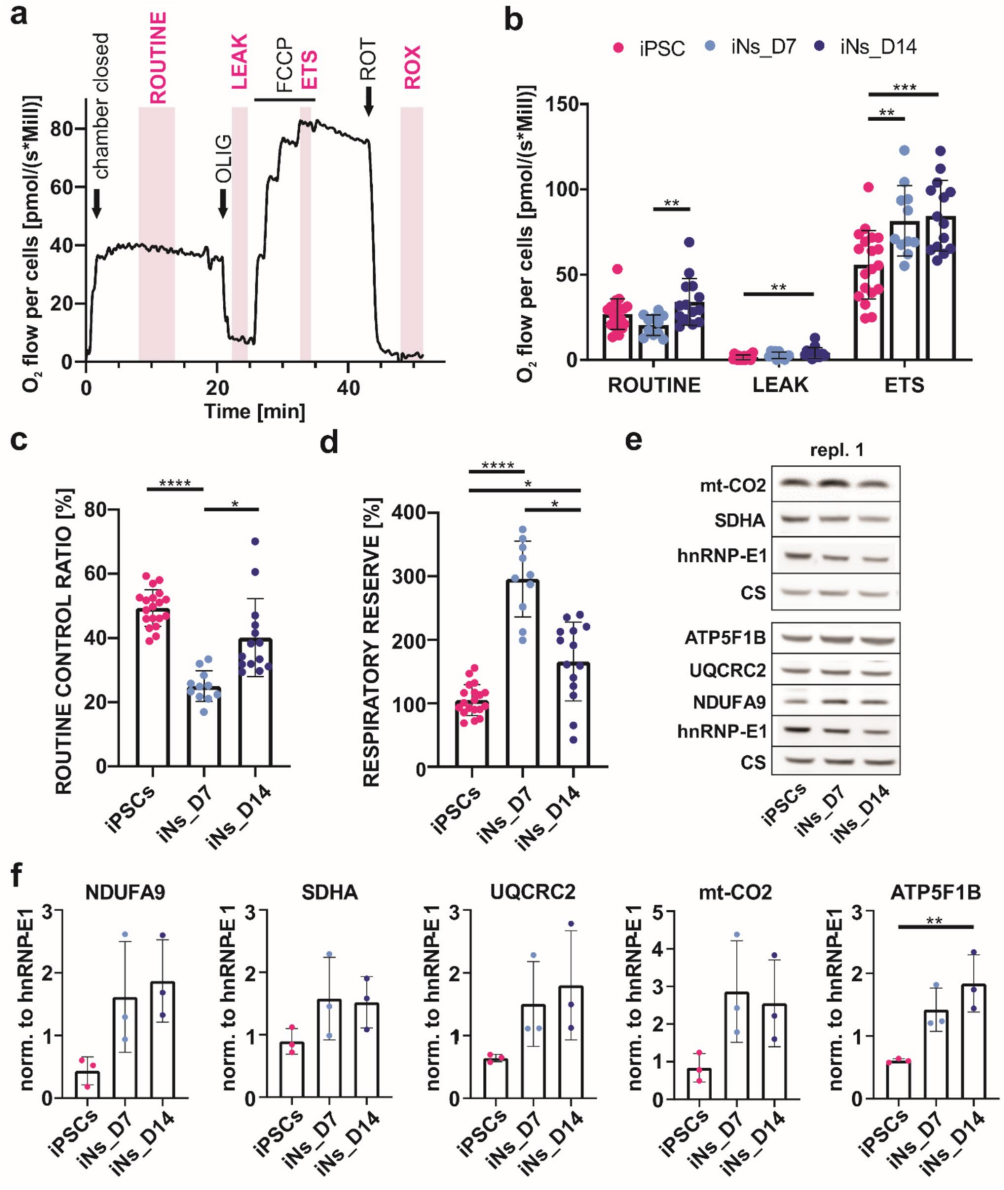
Neuronal differentiation is associated with increased mitochondrial respiratory capacity and mitochondrial biogenesis. **a** Schematic representation of the high-resolution respirometry protocol performed on intact dissociated cells using the Oxygraph-2 k system. The measurement starts with the closing of the measurement chamber (chamber closed), followed by the assessment of routine respiration (ROUTINE), the addition of oligomycin (OLIG) to determine leak respiration (LEAK), carbonylcyanide-p-trifluoromethoxyphenylhydrazone (FCCP) to induce the maximum capacity of the electron transport system (ETS), and rotenone (ROT) to measure residual oxygen consumption (ROX). **b** Oxygen consumption rates normalized to cell number show increased ETS capacity in both iNs_D7 and iNs_D14 compared to iPSCs. ROX was subtracted from all other values to correct for non-mitochondrial respiration. Samples for Oxygraph measurements: *n* = 19 (iPSCs), *n* = 10 (iNs_D7), and *n* = 14 (iNs_D14). **c** Routine control ratio (ROUTINE/ETS*100 ratio) as a measure of the proportion of ETS capacity utilized by the cell during routine respiration (expressed in % of ETS). **d** Respiratory reserve capacity is calculated as (ETS – ROUTINE)/ROUTINE*100 (expressed in % of ROUTINE). **e** Representative Western blot of selected catalytic subunits of mitochondrial OXPHOS complexes NDUFA9, SDHA, UQCRC2, mt-CO2, ATP5F1B, and normalization markers hnRNP-E1 and CS. One representative biological replicate (in technical triplicate); complete Western blot image from all biological replicates is available in Online Resource 1—Fig. S4a. **f** Representative Western blot quantification of subunits of mitochondrial OXPHOS complexes NDUFA9, SDHA, UQCRC2, mt-CO2, and ATP5F1B, normalized to hnRNP-E1 as a nuclear marker. Each dot represents one biological replicate, calculated as the mean of technical triplicates. Statistical analysis was performed using one-way ANOVA followed by Tukey’s multiple comparisons test. For LEAK, routine control ratio, and respiratory reserve capacity, a Kruskal–Wallis test with Dunn’s multiple comparisons was applied. Error bars represent ± SD. (*) *p* ≤ 0.05; (**) *p* ≤ 0.01; (***) *p* ≤ 0.001; (****) *p* ≤ 0.0001. NDUFA9, NADH dehydrogenase [ubiquinone] 1 alpha subcomplex subunit 9, mitochondrial (complex I); SDHA, succinate dehydrogenase [ubiquinone] flavoprotein subunit, mitochondrial (complex II); UQCRC2, cytochrome bc1 complex subunit core2, mitochondrial (complex III); mt-CO2, cytochrome c oxidase subunit 2 (complex IV); ATP5F1B, ATP synthase subunit beta, mitochondrial (complex V)

Following the upregulation of mitochondrial proteins and metabolic pathways remodeling identified by proteomic analyses, we examined the mitochondrial function using high-resolution respirometry. Oxygen consumption measurements were performed on intact cells (iPSCs, iNs_D7, and iNs_D14) using the Oxygraph-2 k system, following a protocol including sequential measurement of routine, leak, ETS, and ROX respiration states (Fig. 4a).

Routine respiration, a measure of the actual cellular metabolic activity, was not changed between the iPSCs and differentiating neurons [49–51]. However, it was significantly higher in iNs_D14 compared to iNs_D7 (*p* = 0.0053), suggesting increased basal respiratory demands in more mature neurons. The rates of leak respiration after inhibiting ATP synthase were negligible in iPSCs as well as in differentiating neurons. This indicates that routine respiration rates genuinely reflect the ATP demand-driven mitochondrial activity. Importantly, the ETS respiratory capacity induced by FCCP was substantially increased in both iNs_D7 (81.6 ± 20.7 pmol/(s*Mill); *p* = 0.0052) and iNs_D14 (84.6 ± 20.8 pmol/(s*Mill); *p* = 0.0007), compared to iPSCs (55.8 ± 20.1 pmol/(s*Mill)). Finally, ROX was measured after ROT treatment and subtracted from all conditions to exclude non-mitochondrial oxygen consumption (Fig. 4b).

To further quantify the mitochondrial functional adaptation, we calculated the routine control ratio and the respiratory reserve capacity. The routine control ratio (routine respiration/ETS capacity*100) was markedly reduced in iNs_D7 (25%) compared to iPSCs (49.3%) and partially restored in iNs_D14 (40%), indicating that early neurons preserve a large respiratory reserve, while more mature neurons utilize a greater proportion of their maximal respiratory capacity (Fig. 4c). The respiratory reserve capacity, calculated as (ETS − routine)/routine*100 respiration, represents the increase in respiration that cells can achieve above their routine respiration. It was approximately threefold higher in iNs_D7 compared to iPSCs (296% vs. 105%) and remained elevated in iNs_D14 (166%) (Fig. 4d), consistent with a progression from a metabolically flexible to a more active respiratory state during neuronal maturation.

To corroborate these functional findings, we analyzed the abundance of OXPHOS proteins by Western blotting (Fig. 4e, Online Resource 1—Fig. S4a), applying two different normalization strategies to address distinct biological questions. First, normalization to the nuclear marker hnRNP-E1 allowed us to assess changes in OXPHOS protein abundance relative to cell number. Using this approach, we observed elevated OXPHOS complex subunit levels in both iNs_D7 and iNs_D14 compared to iPSCs, with a significant increase in ATP synthase subunit beta (ATP5F1B) (complex V subunit; *p* = 0.0091) (Fig. 4f). Notably, the hnRNP-E1 level was higher in iPSCs, likely reflecting their characteristically larger nuclear-to-cytoplasmic ratio associated with pluripotency, where nuclear proteome represents larger fraction of the total cellular proteome. These results were consistent with our LFQ-MS proteomic data, which also demonstrated the upregulation of mitochondrial proteins (MitoCarta 3.0) in neurons. Second, normalization to the mitochondrial matrix enzyme citrate synthase (CS), an established marker for mitochondrial content, enabled us to distinguish whether the increase in OXPHOS proteins was due to an overall rise in mitochondrial content or represented a selective upregulation of the OXPHOS complexes on the mitochondrial membrane relative to other mitochondrial proteins. When normalized to CS, the relative abundance of OXPHOS proteins showed no significant change (Online Resource 1—Fig. S4b), indicating that their upregulation occurs in proportion to mitochondrial expansion. Consistently, elevated CS levels in iNs (Online Resource 1—Fig. S4c) support the interpretation that differentiating cells undergo coordinated mitochondrial biogenesis, with both mitochondrial content and OXPHOS proteins increasing proportionally.

Taken together, our findings demonstrate that neuronal differentiation is accompanied by robust mitochondrial biogenesis and an increase in respiratory capacity. Despite this enhancement, routine respiration in differentiated neurons utilizes only a fraction of their maximal ETS capacity, suggesting the presence of a substantial respiratory reserve. Such reserve capacity may represent an important feature of metabolically flexible neurons, relevant to both development and disease modeling in vitro.

### Progressive Metabolic Shift Toward Oxidative Phosphorylation During Neuronal Differentiation

**Fig. 5:**
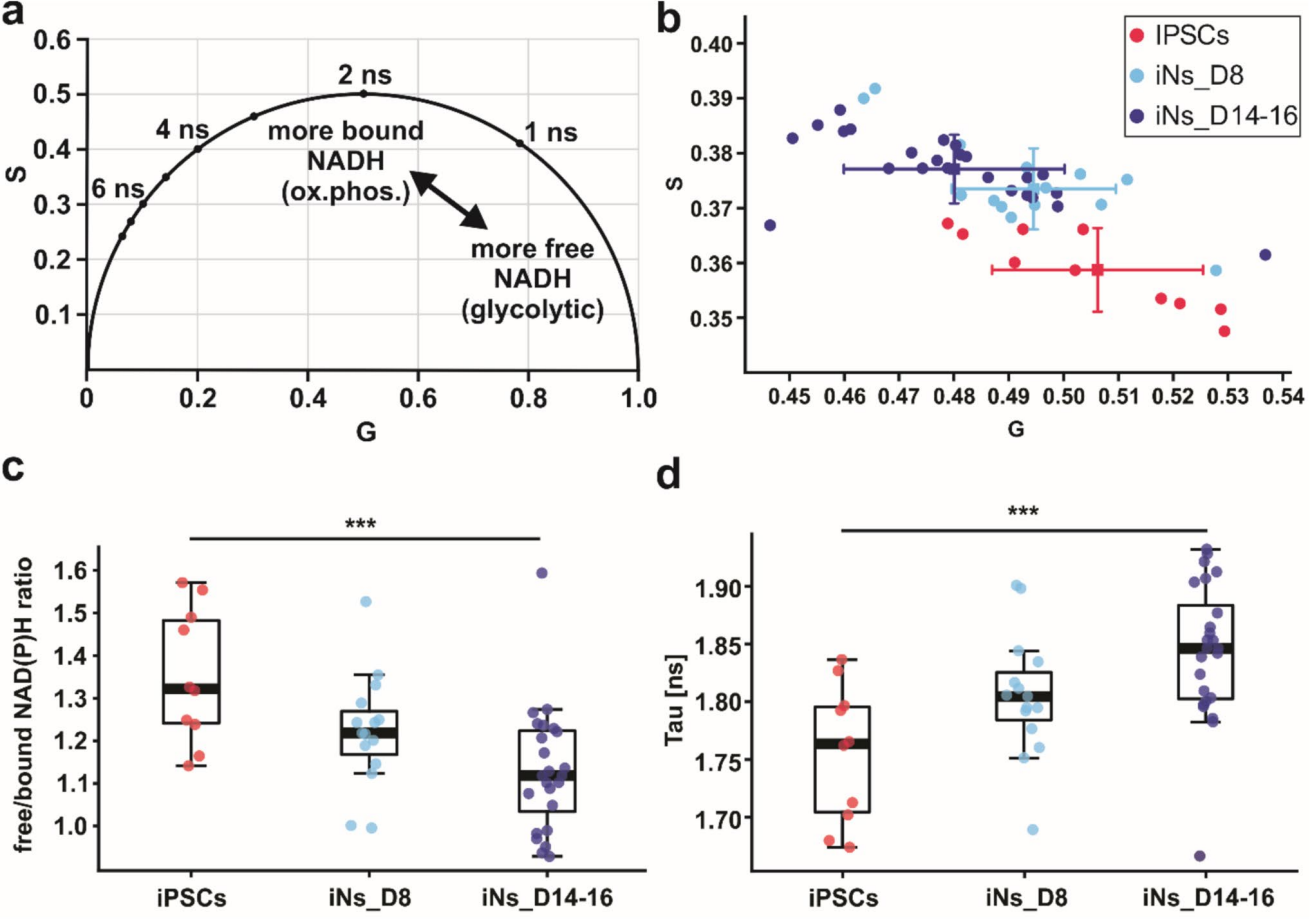
Fluorescence lifetime imaging microscopy (FLIM) analysis shows a metabolic shift from glycolysis to oxidative phosphorylation during neuronal differentiation. **a** Schematic representation of NAD(P)H phasor plot. G and S represent the real and imaginary components of FLIM phasor space. **b** Section of the phasor plot showing the coordinates of basal NAD(P)H fluorescence; each dot represents an individual measurement. **c** Free/bound NAD(P)H ratio [%] and **d** intensity-weighted fluorescence lifetime (Tau [ns]) measured by FLIM in human iPSCs at day 8 (iNs_D8) and at days 14 to 16 (iNs_D14–16) under basal conditions. A decrease in the free/bound NAD(P)H ratio and an increase in Tau during differentiation indicate a shift toward OXPHOS. Sample size: *n* = 10 (iPSCs), *n* = 15 (iNs_D8), and *n* = 24 (iNs_D14-16). Statistical analysis was performed using one-way ANOVA followed by Tukey’s multiple comparisons test. Error bars represent ± SD. (***) *p* ≤ 0.001

To further validate the metabolic shift at the single-cell level, we performed FLIM to assess the mitochondrial function and redox status during neuronal differentiation. This approach allowed us to measure the fluorescence lifetime of NAD(P)H and distinguish between its free and enzyme-bound forms, providing quantitative information on cellular metabolic state. Free NAD(P)H exhibits a short fluorescence lifetime (< 0.4 ns), whereas protein-bound NAD(P)H shows a much longer lifetime (typically in a range of 1.9 to 5.7 ns). The ratio of free to bound NAD(P)H, along with the fluorescence lifetime (Tau), is commonly used as indicators of metabolic activity related to oxidative metabolism [52–57]. We analyzed NAD(P)H autofluorescence in iPSCs, iNs at day 8 (iNs_D8), and days 14 to 16 (iNs_D14-16) under basal conditions. We observed a progressive decrease in the free/bound NAD(P)H ratio during differentiation, with statistically significant reductions between iPSCs and iNs_D14-16 (Fig. 5c). This decline in the free/bound NAD(P)H ratio indicates a decreased pool of free NAD(P)H and a shift toward its enzyme-bound form. In parallel, the fluorescence lifetime (Tau) gradually increased from iPSCs to iNs_D14-16, suggesting enhanced participation of NAD(P)H in OXPHOS pathways (Fig. 5d).

These findings suggest that the proportion of enzyme-bound NAD(P)H increases with differentiation, indicating a sustained and progressive metabolic shift from glycolysis toward OXPHOS [55–57]. This interpretation is also supported by the phasor plot analysis (Fig. 5a–b), which showed a visible shift along the isoline from iPSCs to iNs_D14-16. These FLIM-based observations align well with our proteomic and respirometry data, which revealed increased ETS capacity and respiratory efficiency, indicating coordinated enhancement of OXPHOS activity during early neuronal maturation.

### Metabolic Rearrangement During Differentiation Reflects Changes in the Way Glucose is Utilized

**Fig. 6:**
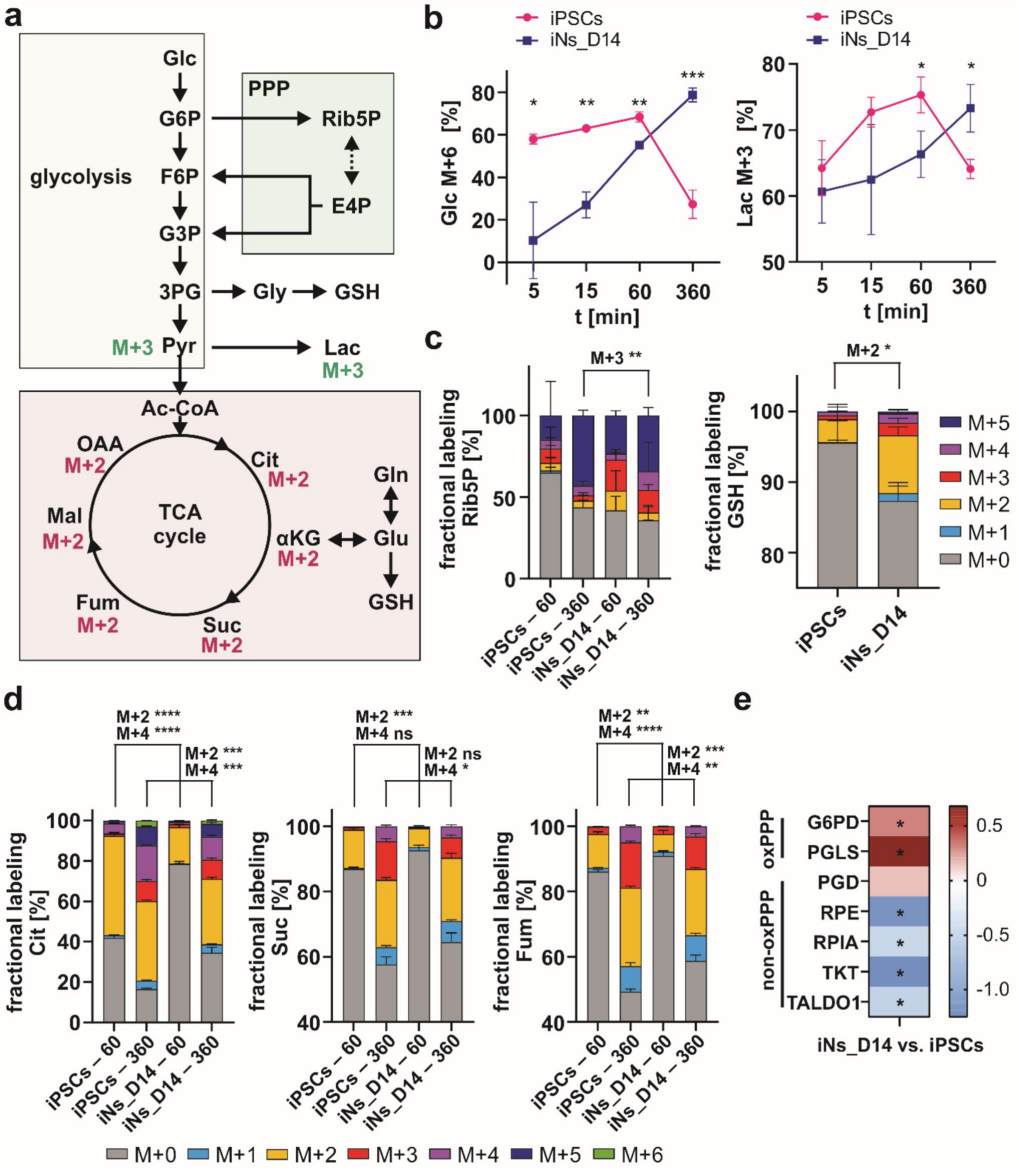
^13^C₆-glucose tracing and proteomics reveal preferential utilization of the pentose phosphate pathway (PPP) and delayed labeling of the intracellular pool of fully labeled glucose, lactate, and tricarboxylic acid (TCA) cycle metabolites in differentiated neurons. **a** Schematic representation of ^13^C incorporation from ^13^C_6_-glucose into glycolysis, the PPP, and the TCA cycle. Isotopomers M + 2 and M + 3 indicate the number of labeled carbons. **b** Time-course of isotopomer distribution of ^13^C-labeled glucose (Glc) (M + 6) and lactate (Lac) (M + 3) at 5, 15, 60, and 360 min after the addition of ^13^C_6_-glucose to the culture medium. The measurement was performed in human iPSCs and iNs_D14 cells. **c** Fractional ^13^C-enrichment of ribose-5-phosphate (Rib5P, %) derived from ^13^C_6_-glucose tracing at 60 and 360 min and glutathione (GSH) at 360 min, reflecting activity of the PPP. Increased M + 2 isotopomer of GSH in iNs_14 supports increased antioxidant defense. **d** Fractional labeling of metabolites of the TCA cycle (60, 360 min) reveals delayed incorporation of glucose-derived carbon into the TCA cycle (e.g., Cit M + 2, Suc M + 2, Fum M + 2). **e** Proteomic LFQ-MS analysis shows increased abundance of enzymes in the oxPPP (e.g., G6PD, PGLS) and downregulation of enzymes in the non-oxidative branch (e.g., TKT, TALDO1), indicating pathway rerouting during neuronal differentiation. Statistical analysis for glucose tracing was performed using an unpaired *t*-test, comparing the same isotopomer (M + X) of a given time point between iPSCs and iNs_D14. Data represent mean ± SD from three independent experiments. (ns) *p* > 0.05; (*) *p* ≤ 0.05; (**) *p* ≤ 0.01; (***) *p* ≤ 0.001; (****) *p* ≤ 0.0001. For proteomic data from three independent experiments, asterisk indicates significance (log_2_FC ≥  ± 1; *p* < 0.05). 3PG, 3-phosphoglycerate; αKG, α-ketoglutarate; Ac-CoA, acetyl-coenzyme A; Cit, citrate; E4P, erythrose 4-phosphate; F6P, fructose 6-phosphate; Fum, fumarate; Glc, glucose; G3P, glyceraldehyde-3-phosphate; G6P, glucose 6-phosphate; G6PD, glucose-6-phosphate dehydrogenase; Gln, glutamine; Glu, glutamate; Gly, glycine; Mal, malate; non-oxPPP, non-oxidative pentose phosphate pathway; OAA, oxaloacetate; oxPPP, oxidative pentose phosphate pathway; PGD, phosphogluconate dehydrogenase; PGLS, 6-phosphogluconolactonase; Rib5P, ribose 5-phosphate; RPE, ribulose-phosphate 3-epimerase; RPIA, ribose 5-phosphate isomerase A; Suc, succinate; TALDO1, transaldolase 1; TKT, transketolase

Since glucose is the primary energy source for the brain, we performed a flux analysis using ^13^C₆-glucose to examine the incorporation of glucose-derived carbons into downstream metabolites during in vitro neuronal differentiation. We compared iPSCs and iNs_D14 incubated with labeled glucose for 5, 15, 60, or 360 min and analyzed labeling of glycolytic metabolites, as well as metabolites from downstream pathways such as the tricarboxylic acid (TCA) cycle or the pentose phosphate pathway (PPP) (Fig. 6a). As a result, iPSCs exhibited higher glycolytic activity, as reflected by both increased intracellular M + 6 glucose and glucose-derived lactate (M + 3), particularly at the early time points (5–60 min) (Fig. 6b; Online resource 1—Fig. S6). At 360 min, labeled fractions of both glucose and lactate in iPSCs were reduced. This is most likely due to a depletion of the M + 6 glucose pool rather than a change in glycolytic metabolism. While glucose-derived lactate M + 3 is decreased in 5–60 min, the incorporation of glucose-derived carbons into TCA cycle metabolites was also reduced upon neuronal differentiation as demonstrated by decreased levels of citrate, succinate, and fumarate M + 2 isotopologues throughout the time course (Fig. 6d; Online Resource—Fig. S6). Given that routine respiration was comparable between both cell types, the data suggest that reduced incorporation of glucose-derived carbons into the TCA cycle is likely due to the increasing reliance on alternative substrates such as fatty acids or glutamine [58, 59]. This interpretation is further supported by slower labeling of the intracellular pool of fully labeled glucose in neurons (Fig. 6b), which is reflected in the delayed labeling of TCA cycle intermediates.

Further, we analyzed the metabolic flux of glucose into the PPP, which produces NADPH and ribose-5-phosphate (Rib5P), metabolites important for biosynthesis, redox balance, and de novo nucleotide synthesis. The fractional labeling of Rib5P revealed a different pattern between iPSCs and differentiating neurons, with neurons showing higher overall labeling as well as an increase in the M + 3 isotopomer at 360 min, suggesting alterations in the PPP (Fig. 6c; Online resource 1—Fig. S6). To investigate the PPP pathway further, we analyzed the abundance of PPP enzymes by LFQ proteomics, which revealed higher levels of enzymes of the oxidative branch PPP (ox PPP). In particular, glucose-6-phosphate 1-dehydrogenase (G6PD), the rate-limiting enzyme of oxPPP, was significantly upregulated and is involved in the conversion of glucose 6-phosphate to 6-phosphoglucono-1,5-lactone, with concomitant production of NADPH [60]. On the other hand, almost all enzymes of non-oxidative PPP (non-oxPPP) are significantly downregulated, which is consistent with a shift toward the oxidative branch and thus with an increased potential for NADPH production (Fig. 6e). NADPH plays a crucial role in the synthesis of various biomolecules, such as fatty acids and amino acids, and is also essential for maintaining the cell’s antioxidant defenses by donating electrons to reduce oxidized glutathione. The possibility that differentiated neurons need higher antioxidant capacity is further supported by observed rewiring of glucose to glutathione, as the glucose-derived glutathione M + 2 levels are significantly increased in differentiated neurons relative to iPSCs (Fig. 6c). This observation aligns with the proteomic enrichment of mitochondrial antioxidant pathways in iNs (GOBP Cellular Response To Oxidative Stress; data not shown). Together, these findings highlight a metabolic rewiring during neuronal differentiation, characterized by slower accumulation of the labeled intracellular glucose pool in neurons compared to iPSCs, which may underlie the reduced flux into the TCA cycle. Instead, labeled glucose appears to be increasingly directed into the oxPPP and glutathione biosynthesis, supporting enhanced antioxidant capacity.

## Discussion

**Fig. 7:**
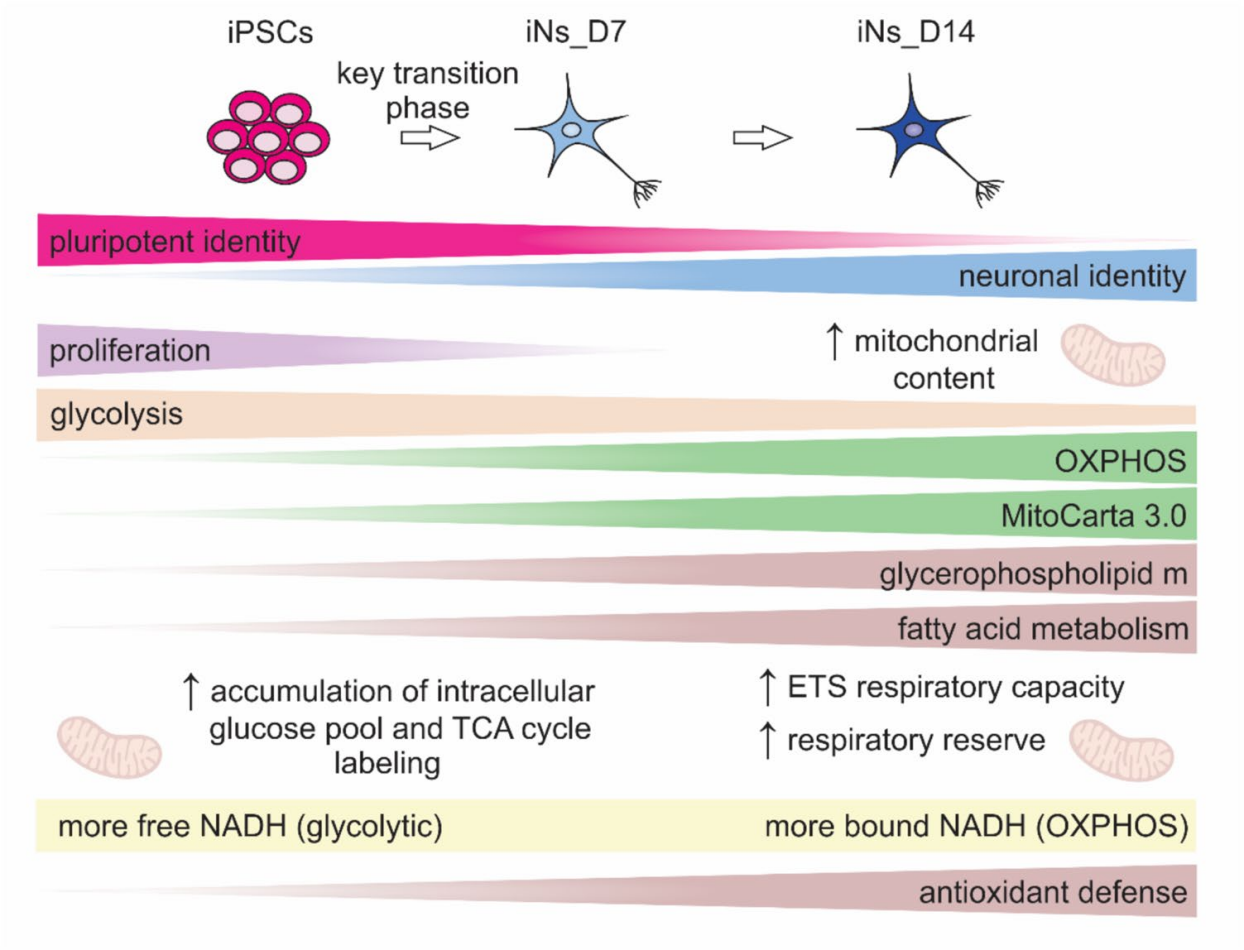
Schematic overview summarizing the temporal trajectory and coordination of key metabolic pathways during early neuronal maturation. ETS, electron transport system; OXPHOS, oxidative phosphorylation; TCA, tricarboxylic acid. Created in BioRender

In this study, we present a comprehensive, multi-modal characterization of metabolic remodeling during neuronal differentiation from human iPSCs. By integrating proteomics, high-resolution respirometry, FLIM, and ^13^C metabolic flux analysis, we uncover a coordinated transition toward oxidative metabolism, early mitochondrial biogenesis, and a rerouting of glucose utilization. Our findings show that early neuronal maturation is accompanied by increased mitochondrial content, enhanced respiratory capacity and function, a metabolic shift toward OXPHOS, and redirection of glucose-derived carbons toward biosynthetic and antioxidant pathways. Importantly, we identify the first week of differentiation as a critical window of metabolic specialization, establishing a functional reference framework for modeling human neurodevelopmental and mitochondrial disease. Our results are summarized in a schematic overview (Fig. 7) that highlights the temporal trajectory and coordination of key metabolic pathways involved in early neuronal maturation.

### Mitochondrial Biogenesis and Metabolic Shift

Our findings reveal that the metabolic transition toward OXPHOS occurs very early during NGN2-induced neuronal differentiation from human iPSCs. This shift is functionally supported by increased respiratory ETS capacity, elevated levels of mitochondrial proteins, and an expanding pool of enzyme-bound NAD(P)H. By integrating proteomics, high-resolution respirometry, and NAD(P)H FLIM, we provide direct functional evidence of this remodeling, extending previous transcriptomic and proteomic analyses [2, 3, 25]. In agreement with earlier reports, we observe features consistent with mitochondrial biogenesis, including upregulation of key regulators such as peroxisomal proliferating activating receptor *γ* coactivator-1*α* (PGC-1*α*) and mitochondrial transcription factor A (TFAM), as well as reduced expression of key glycolytic enzymes, exemplified by downregulation of hexokinase 2 (HK2, data not shown). Pathway-level enrichment of oxidative metabolism and MitoCarta 3.0 proteins, together with increased ETS capacity, further supports the view that mitochondrial activation is an early, coordinated, and conserved feature of neuronal differentiation. Furthermore, the conserved nature of this metabolic switch across various neuronal subtypes has been previously noted [2]. Notably, this metabolic transition is paralleled by nuclear remodeling, as indicated by the stronger hnRNP-E1 signal in iPSCs, which likely reflects their characteristically large nuclear volume, a hallmark of pluripotency.

Importantly, our data emphasize that mitochondrial biogenesis is initiated early during neuronal differentiation rather than representing a late maturation event. This process is likely driven by transcriptional activation of mitochondrial regulators, including PGC-1α and nuclear respiratory factor 1 (NRF-1), as shown in neuroprogenitor cells after inhibition of histone deacetylase (HDAC) [61]. In that study, epigenetic modulation induced mitochondrial DNA replication, increased expression of key biogenesis factors, and enhanced the OXPHOS function. The observed increase in the respiratory reserve capacity and levels of mitochondrial enzymes observed in our system is consistent with this model, supporting the interpretation that mitochondrial remodeling is a tightly regulated early event aligned with the increasing energetic and biosynthetic demands of developing neurons.

Moreover, our findings reveal the rewiring of glucose-derived metabolites during neural maturation, indicative of increased metabolic flexibility. Such flexibility may be critical for localized ATP generation in regions distant from mitochondria, such as axonal growth cones, where glycolytic enzymes have been shown to accumulate [10, 62]. Supporting this compartmentalized energy strategy, synaptic mitochondria have been shown to express lower levels of OXPHOS subunits and calcium-buffering proteins than their non-synaptic counterparts, indicating limiting oxidative capacity and highlighting the importance of glycolytic ATP production at the synapse [63]. This spatially adaptive metabolic flexibility also facilitates regenerative processes, as glycolytic ATP and sustained mitochondrial transport are required for efficient axonal regrowth [64]. Taken together, our data support the view that mitochondrial activation is an early, conserved, and functionally integrated feature of neuronal differentiation, during which neurons establish a metabolically flexible phenotype combining sustained glycolytic activity with enhanced OXPHOS.

### Rewiring of Glucose Metabolism During Neuronal Differentiation

By applying ^13^C_6_-glucose metabolic tracing, we uncovered an early and dynamic reorganization of glucose utilization during neuronal differentiation. iPSCs exhibited a robust glycolytic activity [65, 66], as reflected by the rapid accumulation of fully labeled intracellular glucose (M + 6) and its subsequent conversion to lactate (M + 3). In contrast, iNs displayed a slower increase in both glucose and lactate pools. At timepoint 360 min, we observed a pronounced drop in both fully labeled glucose and lactate in iPSCs, most likely reflecting depletion of the extracellular glucose supply (5 mM) in the medium, which may have contributed to the onset of gluconeogenesis, highlighting a metabolic adaptation in response to nutrient availability [67]. Importantly, the slower labeling of both glucose and lactate pools in neurons may indicate a reduced rate of glucose uptake, which might account for delays in the labeling of intermediates in the TCA cycle. These observations are therefore consistent with the interpretation that neurons may not utilize glucose as their predominant metabolic substrate and rely more on alternative substrates to support mitochondrial respiration [58, 59], indicating a well-regulated metabolic reorganization as differentiation progresses.

Our proteomic data further support a shift toward alternative substrates fueling mitochondrial respiration. Enrichment of fatty acid and amino acid metabolism pathways suggests that iPSC-derived neurons rely less on glucose oxidation and more on other alternative substrates to fuel OXPHOS. These findings align with previous studies showing the utilization of fatty acid oxidation by early neurons or progenitors and glutamine oxidation as a key anaplerotic input into the TCA cycle in maturing neurons [68–70]. Notably, iPSC-derived neurons retain the enzymatic capacity to metabolize glucose and incorporate it into the TCA cycle even in the absence of glial support [71]. In summary, neuronal differentiation involves a metabolic rewiring with delayed glucose contribution to the TCA cycle and increased reliance on alternative substrates.

### Enhanced Redox Homeostasis via Upregulation of the Pentose Phosphate Pathway

Our study reveals a rearrangement of the PPP, increased levels of oxPPP enzymes, and higher production of glutathione from glucose-derived metabolites during early neuronal differentiation, suggesting an adaptive response to a potential increase in oxidative stress. Enhanced glutathione labeling in neurons may derive from glucose carbons routed through 3-phosphoglycerate to glycine synthesis or via glucose-derived glutamate from the TCA cycle [72, 73]. The oxidative branch of the PPP generates NADPH, which is required for redox homeostasis and biosynthetic processes. This observation is consistent with previous studies demonstrating that PPP-derived NADPH is essential for glutathione regeneration and protection against oxidative damage [74, 75]. Indeed, glutathione depletion has been shown to increase oxidative stress in neurons [76, 77].^21^

Importantly, moderate levels of reactive oxygen species (ROS) have been shown to promote neuronal differentiation, whereas excessive oxidative stress impairs this process, highlighting the need for a tightly regulated redox environment [74, 78, 79], as also reflected by the enrichment of antioxidant pathways in our proteomic data and by enhanced glutathione labeling. The rearranged PPP pathway in our system thus likely represents an early metabolic adaptation to meet the antioxidant and signaling demands of differentiating neurons. In line with their long-term survival demands, postmitotic neurons retain elements of the DNA replication machinery, including cyclins and DNA polymerases, which may facilitate the genome maintenance and repair during early differentiation (iNs_D7–iNs_D14) [80].

Future studies should explore how modulation of the oxidative PPP and NADPH-dependent antioxidant pathways impacts neuronal differentiation and maturation. This may yield therapeutic insights, particularly in neurodevelopmental or neurodegenerative disorders characterized by redox imbalance or impaired neurogenesis.

### Understanding Normal Brain Development

Our data reveal that iNs recapitulate key metabolic transitions observed during early human neurodevelopment. In line with in vivo evidence [2, 3, 81], we observed coordinated shift from glycolysis toward OXPHOS, increased mitochondrial content, and enhanced redox capacity. Notably, iNs retained glycolytic activity at day 14, reflecting a fetal-like metabolic profile consistent with high biosynthetic and antioxidant demands during neurogenesis and synaptogenesis [71, 82]. This stage is also particularly vulnerable to oxidative damage due to the limited mtDNA repair capacity [83–85]. While previous transcriptomic studies mapped iNs_D7 to 8–22 weeks post-conception and iNs_D42 to postnatal day 86 [21], our data provide complementary metabolic validation of these developmental stages. This underscores the value of iNs as a tractable and temporally defined system for studying human neurodevelopmental bioenergetics.

### Disease Modeling

The cortical iNs, along with their metabolic characterization approach presented in this study, provide a robust and translationally relevant model to investigate metabolic alterations in both neurodevelopmental and neurodegenerative disorders. This system is especially relevant for conditions involving mitochondrial dysfunction or redox imbalance, such as Leigh syndrome or cyclin-dependent kinase-like 5 (*CDKL5*) deficiency disorder, where impaired OXPHOS, altered mitochondrial dynamics, and increased glycolytic reliance have been documented [12, 86, 87]. Using our integrated methodology (combining NAD(P)H-FLIM, proteomics, fluxomics, and high-resolution respirometry), such disease-specific phenotypes can be functionally validated and quantitatively assessed in human neurons.

Our approach also enables the study of redox-related phenotypes in Parkinson’s disease (e.g., *PARK2* mutations), Huntington’s disease, and other conditions characterized by defective respiratory capacity and oxidative stress [82, 88, 89]. The enhanced PPP flux and increased glucose-derived labeling of glutathione (Fig. 6) may serve as sensitive readouts of antioxidant adaptation in disease-relevant models.

Furthermore, this system offers a tractable model for investigating metabolic stress and nutrient adaptation. The observed metabolic flexibility, particularly delayed glucose oxidation and compensatory substrate use, can be harnessed to model pathological states such as GLUT1 deficiency, neonatal hypoglycemia, or ketogenic interventions. Controlled substrate manipulation (e.g., lactate, ketone bodies, low/high glucose) allows direct interrogation of neuronal resilience and adaptation mechanisms [90, 91].

Together, these findings highlight the potential of iNs as a functional and scalable model for studying the pathophysiological impact of metabolic impairments in human neurons across developmental and disease contexts.

### Limitations and Advances

While our study offers a multidimensional and functionally validated characterization of early neuronal metabolic remodeling, several limitations related both to the model and the study design must be acknowledged. The iNs model offers a valuable platform for studying human neurodevelopmental bioenergetics. However, as an in vitro system, it lacks the full physiological complexity of the in vivo brain environment, particularly the absence of glial cells, vascular elements, and extracellular matrix components. For instance, co-culture of iNs with astrocytes has been shown to better reflect in vivo metabolic dynamics through the astrocyte-neuron lactate shuttle and functional maturation of the model [92, 93]. Moreover, the current 2D monoculture lacks the three-dimensional structural complexity and spatial organization of the developing brain. Future development of 3D brain organoids may more accurately recapitulate the tissue-level architecture and cell–cell metabolic interactions. Furthermore, our study focused exclusively on excitatory cortical neurons. Other neuronal subtypes, such as inhibitory interneurons or dopaminergic neurons, may exhibit different metabolic profiles during differentiation. For example, GABAergic interneurons (e.g., parvalbumin-positive neurons) possess different redox dynamics and delayed antioxidant system maturation, making them particularly vulnerable to oxidative stress during critical developmental windows [94, 95]. Additionally, while our findings confirm the association between the metabolic shift and neuronal maturation, we did not employ pathway-specific perturbations (e.g., pharmacological or genetic inhibition of the PPP) to determine causality. The conclusions thus remain descriptive rather than mechanistic. By focusing on the first 14 days of differentiation, our study concentrates on the early phases of neurodevelopment but does not include the later phases, such as synaptic maturation, network formation, or metabolic establishment. A further limitation is that respirometry measurements were performed on dissociated single-cell suspensions. Although short-term suspension has been shown not to affect routine or leak respiration, with differences in ETS capacity attributed to methodological factors rather than to cell suspension itself [49], this approach may still introduce some stress and should be considered. Finally, FLIM-derived NAD(P)H lifetimes reflect shifts in the redox state and metabolic engagement but can also be influenced by changes in the overall NAD(H) pool size [54]. Therefore, they do not identify the specific pathways or functional outcomes involved, necessitating validation through additional metabolic and proteomic analyses. Nonetheless, the NGN2-iPSC-derived cortical neuron model provides a rapid, efficient, and reproducible and widely used system for investigating neuronal metabolism, compared with spontaneous differentiation or small molecule–driven differentiation, which require several weeks to months and often generate more heterogeneous populations [22, 93, 96–99]. Additionally, viral overexpression approaches can result in random genomic integration, leading to variable transgene expression and inconsistent differentiation timing [100]. The NGN2-iPSC protocol allows the generation of neurons suitable for metabolic analyses without the need for complex co-culture systems or media. iPSCs also allow genetic manipulation, including the introduction of patient-specific mutations. Moreover, NGN2-induced neurons exhibit functionally mature electrophysiological properties, including reliable synaptic transmission and action potential firing, making them suitable for studies of human neuronal network activity [101–103]. This makes the model a practical platform for systematic and high-throughput studies of neurodevelopmental metabolic dynamics and their pathology. While these advantages make the model highly useful for in vitro applications, addressing its limitations in future studies will deepen our understanding of neuronal metabolic development and enhance its translational potential.

## Conclusion

In summary, our study provides an integrated and functionally validated characterization of metabolic remodeling during early NGN2-mediated neuronal differentiation. We show that the transition from iPSCs to neurons is accompanied by coordinated changes in mitochondrial function, energy metabolism, and redox balance, with the first week emerging as a decisive period of bioenergetic specialization. An important strength of our approach is the use of a neuron-only system, which allows us to define metabolic trajectories intrinsic to neurons without interference from non-neuronal cell types. By combining proteomics, functional assays, and metabolic flux analysis, we establish a multi-modal reference framework that links molecular and functional aspects of neuronal maturation. This comprehensive view establishes NGN2-induced neurons as a metabolically validated model system and offers a foundation for future investigations of neurodevelopmental and mitochondrial disorders. Our study thus provides a robust and tractable model for uncovering metabolic disruptions underlying human neurodevelopmental and neurodegenerative diseases.

## Supplementary Information

Below is the link to the electronic supplementary material.

ESM 1 Supplementary Material 1: Supplementary Figures. (PDF 1.56 MB)

ESM 2 Supplementary Material 2: Supplementary Methods. (PDF 227 KB)

ESM 3 Supplementary Material 3: Table of the 50 most differentially expressed proteins in iPSCs, iNs_D7 and iNs_D14. (XLSX 21.6 KB)

ESM 4 Supplementary Material 4: Zipped folder containing uncropped Western blot images, quantification reports, and a descriptive summary file. (ZIP 4.70 MB)

## Data Availability

The mass spectrometry proteomics data have been deposited to the ProteomeXchange Consortium via the PRIDE partner repository with the dataset identifier PXD064787.

## Abbreviations

3PG: 3-Phosphoglycerate
αKG: α-Ketoglutarate
Ac-CoA: Acetyl-coenzyme A
ATP: Adenosine triphosphate
ATP5F1B: ATP synthase subunit beta
BDNF: Brain-derived neurotrophic factor
CDKL5: Cyclin-dependent kinase-like 5
Cit: Citrate
CM: Cortical neuron medium
CS: Citrate synthase
Ct: Cycle threshold
DCX: Doublecortin
dox: Doxycycline
DPBS: Dulbecco’s phosphate-buffered saline
E4P: Erythrose 4-phosphate
E8: Essential 8
ETS: Electron transport system
F6P: Fructose 6-phosphate
FC: Fold change
FCCP: Carbonylcyanide-p-trifluoromethoxyphenylhydrazone
FLIM: Fluorescence lifetime imaging microscopy
Fum: Fumarate
G3P: Glyceraldehyde-3-phosphate
G6P: Glucose 6-phosphate
G6PD: Glucose-6-phosphate dehydrogenase
GABA: γ-Aminobutyric acid
Glc: Glucose
Gln: Glutamine
Glu: Glutamate
GLUT1: Glucose transporter 1
Gly: Glycine
GOBP: Gene Ontology Biological Process
GSEA: Gene Set Enrichment Analysis
GSH: Glutathione
HDAC: Histone deacetylase
HILIC: Hydrophilic interaction chromatography
hnRNP-E1: Heterogeneous nuclear ribonucleoprotein E1
iNs: NGN2-induced neurons
iNs_D7: NGN2-induced neurons at day 7
iNs_D14: NGN2-induced neurons at day 14
iPSCs: Induced pluripotent stem cells
IRF: Instrument response function
KEGG: Kyoto Encyclopedia of Genes and Genomes
LC-MS: Liquid chromatography-mass spectrometry
LFQ-MS: Label-free quantitative mass spectrometry
NAD(P)H: Nicotinamide adenine dinucleotide (phosphate)
NANOG: Nanog homeobox
NDUFA9: NADH dehydrogenase [ubiquinone] 1 alpha subcomplex subunit 9
NDUFS4: NADH dehydrogenase [ubiquinone] iron-sulfur protein 4
NES: Normalized enrichment score
NGN2: Neurogenin-2
NIM: Neuronal induction medium
non-oxPPP: Non-oxidative pentose phosphate pathway
NRF-1: Nuclear respiratory factor 1
NT-3: Neurotrophin-3
Mal: Malate
MAP2: Microtubule-associated protein 2
MELAS: Mitochondrial encephalomyopathy, lactic acidosis, and stroke-like episodes syndrome
mt-CO2: Cytochrome c oxidase subunit 2
OCT4: Octamer-binding transcription factor 4
OAA: Oxaloacetate
OLIG: Oligomycin
OXPHOS: Oxidative phosphorylation
oxPPP: Oxidative pentose phosphate pathway
PARK2: Parkinson protein 2
PGC-1*α*: Proliferating activating receptor *γ* coactivator-1*α*
PGD: Phosphogluconate dehydrogenase
PGLS: 6-Phosphogluconolactonase
PPP: Pentose phosphate pathway
RT-qPCR: Reverse transcription quantitative polymerase chain reaction
RBFOX3: RNA binding fox-1 homolog 3
REX1: Reduced expression 1 (zinc finger protein 42)
Rib5P: Ribose 5-phosphate
ROS: Reactive oxygen species
ROT: Rotenone
ROX: Residual oxygen consumption
RPE: Ribulose-phosphate 3-epimerase
RPIA: Ribose 5-phosphate isomerase A
SDHA: Succinate dehydrogenase [ubiquinone] flavoprotein subunit
SOX2: SRY-box transcription factor 2
Suc: Succinate
SURF1: Surfeit locus protein 1
SYP: Synaptophysin
TALDO1: Transaldolase 1
TBP: TATA-box-binding protein
TCA: Tricarboxylic acid
TFAM: Mitochondrial transcription factor A
TKT: Transketolase
tRNA: Transfer ribonucleic acid
TUBB3: Tubulin beta 3 class III
UQCRC2: Cytochrome b-c1 complex subunit 2
VTN: Vitronectin
