# Supplementary material for: Development of Cellular Energy Metabolism During Differentiation of Human iPSCs into Cortical Neurons
Source: Mol Neurobiol. 2025 Nov 13;63(1):37. doi: 10.1007/s12035-025-05284-8 (PMC12615542; doi:10.1007/s12035-025-05284-8)
Supplement: Supplementary file 1 — Supplementary Material 1: Supplementary Figures. (PDF 1.56 MB) [file 12035_2025_5284_MOESM1_ESM.pdf]

Online Resource 1

Title:
Development of Cellular Energy Metabolism During Differentiation of Human iPSCs Into Cortical Neurons

Authors:
Šárka Danačíková[1,2,3,4], Petr Pecina[5], Alena Pecinová[5], Jan Svoboda[1], David Vondrášek[6], Davide Alessandro Basello[6], Tomáš Čajka[7], Daniel Hadraba[6], Tomáš Mráček[5], Vladimír Kořínek[3], Jakub Otáhal[1,2]*

[1]Department of Pathophysiology, Second Faculty of Medicine, Charles University, Prague, Czech Republic
[2]Laboratory of Developmental Epileptology, Institute of Physiology of the Czech Academy of Sciences, Prague, Czech Republic
[3]Laboratory of Cell and Developmental Biology, Institute of Molecular Genetics of the Czech Academy of Sciences, Prague, Czech Republic
[4]Department of Physiology, Faculty of Science, Charles University, Prague, Czech Republic
[5]Laboratory of Bioenergetics, Institute of Physiology of the Czech Academy of Sciences, Prague, Czech Republic
[6]Laboratory of Biomathematics, Institute of Physiology of the Czech Academy of Sciences, Prague, Czech Republic
[7]Laboratory of Metabolomics, Institute of Physiology of the Czech Academy of Sciences, Prague, Czech Republic

Jakub Otáhal e-mail: jakub.otahal@lfmotol.cuni.cz

Online Resource 1 – Supplementary Figures

Fig. S1 DNA methylation status of individual CpG sites in the *NANOG* and *OCT4* gene promoters in iPSCs, determined by bisulfite sequencing

Fig. S2 Proteomic profiling and metabolic pathway alterations during differentiation of human iPSCs into iNs (iNs_D7 and iNs_D14), based on LFQ-MS analysis

Fig. S3 Differential expression of proteins associated with key metabolic pathways during neuronal differentiation of human iPSCs

Fig. S4 Neuronal differentiation is associated with increased mitochondrial respiratory capacity and mitochondrial biogenesis

Fig. S5 Proteomic analysis of pluripotency and neuronal markers during differentiation

Fig. S6 Time-course of $^{13}C_6$-glucose fractional labeling for selected metabolites in iNs_D14 compared to iPSCs

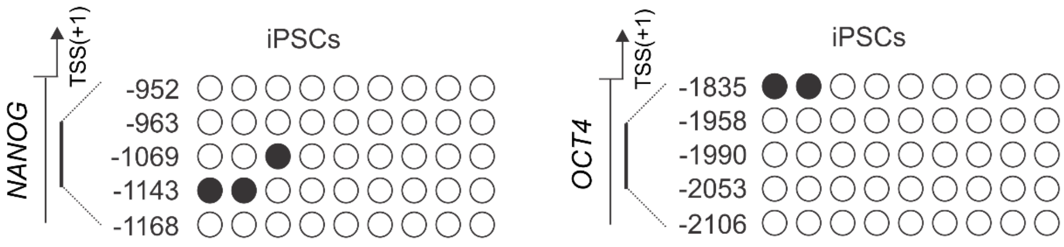

Fig. S1 DNA methylation status of individual CpG sites in the *NANOG* and *OCT4* gene promoters in iPSCs, determined by bisulfite sequencing. White circles represent unmethylated CpG sites, black circles represent methylated CpG sites. Coordinates are based on the GRCh38 reference genome (Ensembl annotation version).

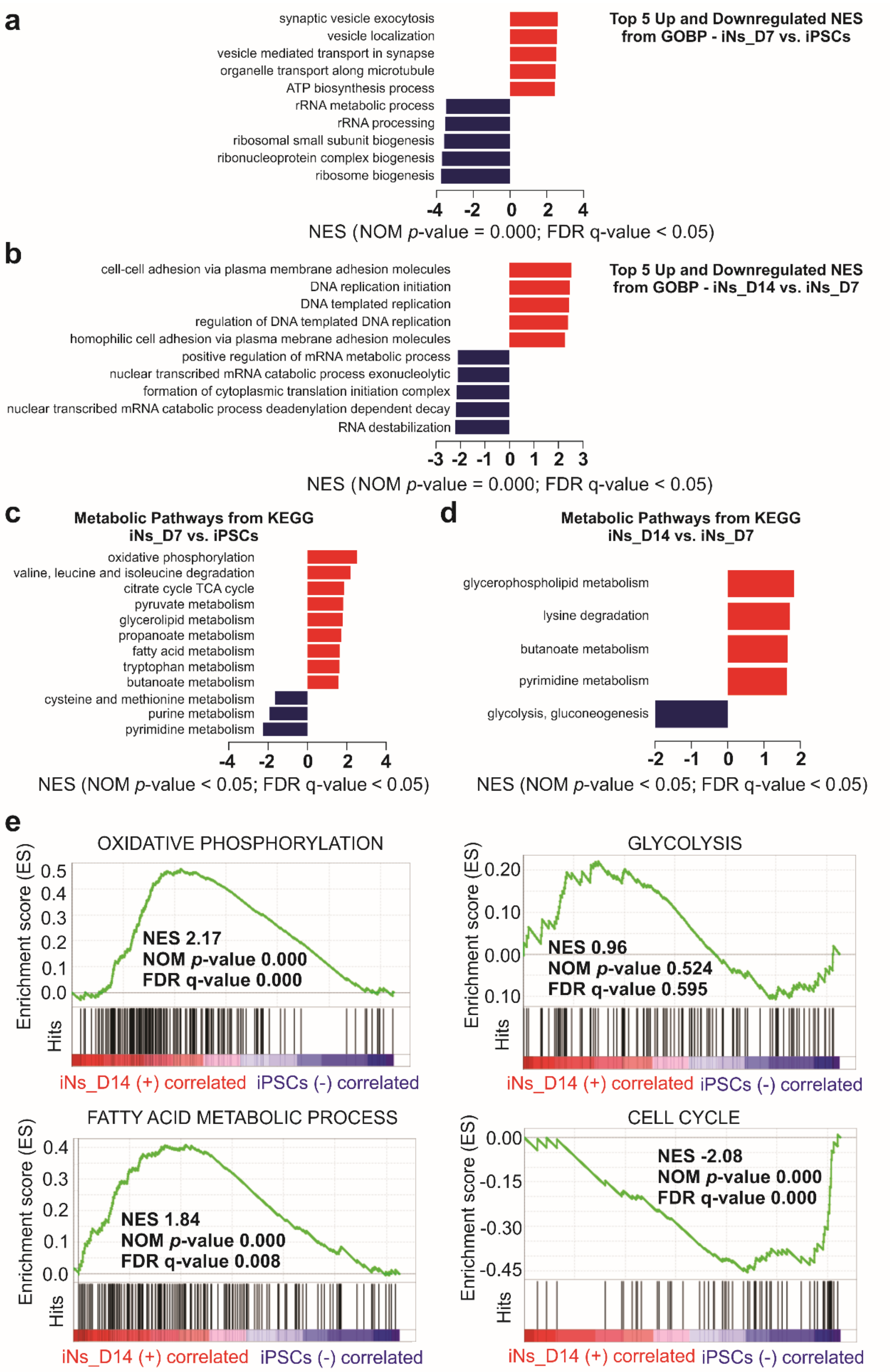

a
Top 5 Up and Downregulated NES from GOBP - iNs_D7 vs. iPSCs
synaptic vesicle exocytosis
vesicle localization
vesicle mediated transport in synapse
organelle transport along microtubule
ATP biosynthesis process
rRNA metabolic process
rRNA processing
ribosomal small subunit biogenesis
ribonucleoprotein complex biogenesis
ribosome biogenesis
-4 -2 0 2 4
NES (NOM p-value = 0.000; FDR q-value < 0.05)
b
Top 5 Up and Downregulated NES from GOBP - iNs_D14 vs. iNs_D7
cell-cell adhesion via plasma membrane adhesion molecules
DNA replication initiation
DNA templated replication
regulation of DNA templated DNA replication
homophilic cell adhesion via plasma mebrane adhesion molecules
positive regulation of mRNA metabolic process
nuclear transcribed mRNA catabolic process exonucleolytic
formation of cytoplasmic translation initiation complex
nuclear transcribed mRNA catabolic process deadenylation dependent decay
RNA destabilization
-3 -2 -1 0 1 2 3
NES (NOM p-value = 0.000; FDR q-value < 0.05)
c
Metabolic Pathways from KEGG iNs_D7 vs. iPSCs
oxidative phosphorylation
valine, leucine and isoleucine degradation
citrate cycle TCA cycle
pyruvate metabolism
glycerolipid metabolism
propanoate metabolism
fatty acid metabolism
tryptophan metabolism
butanoate metabolism
cysteine and methionine metabolism
purine metabolism
pyrimidine metabolism
-4 -2 0 2 4
NES (NOM p-value < 0.05; FDR q-value < 0.05)
d
Metabolic Pathways from KEGG iNs_D14 vs. iNs_D7
glycerophospholipid metabolism
lysine degradation
butanoate metabolism
pyrimidine metabolism
glycolysis, gluconeogenesis
-2 -1 0 1 2
NES (NOM p-value < 0.05; FDR q-value < 0.05)
e
OXIDATIVE PHOSPHORYLATION
Enrichment score (ES)
0.5 0.4 0.3 0.2 0.1 0.0
NES 2.17
NOM p-value 0.000
FDR q-value 0.000
Hits
iNs_D14 (+) correlated
iPSCs (-) correlated
GLYCOLYSIS
Enrichment score (ES)
0.20 0.10 0.00 0.10
NES 0.96
NOM p-value 0.524
FDR q-value 0.595
Hits
iNs_D14 (+) correlated
iPSCs (-) correlated
FATTY ACID METABOLIC PROCESS
Enrichment score (ES)
0.4 0.3 0.2 0.1 0.0
NES 1.84
NOM p-value 0.000
FDR q-value 0.008
Hits
iNs_D14 (+) correlated
iPSCs (-) correlated
CELL CYCLE
Enrichment score (ES)
0.00 -0.15 -0.30 -0.45
NES -2.08
NOM p-value 0.000
FDR q-value 0.000
Hits
iNs_D14 (+) correlated
iPSCs (-) correlated

Fig. S2 Proteomic profiling and metabolic pathway alterations during differentiation of human iPSCs into iNs at day 7 (iNs_D7) and day 14 (iNs_D14), based on LFQ-MS analysis. Sample sizes: n = 3 (iPSCs), n = 3 (iNs_D7), n = 4 (iNs_D14).

a-b) Enrichment analysis of GOBP. The top 5 upregulated and downregulated GOPB terms are shown based on normalized enrichment scores (NES). Analyses were performed for (A) iNs_D7 vs. iPSCs and (B) iNs_D14 vs. iNs_D7. Only enriched pathways with NOM *p*-value = 0.000 and FDR q-value < 0.05 were included.

c-d) Enrichment analysis of metabolic pathways (maximum of 10 pathways) based on KEGG database. Significantly upregulated and downregulated metabolic pathways were identified based on NES for (C) iNs_D7 vs. iPSCs and (D) iNs_D14 vs. iNs_D7 (NOM *p*-value < 0.05; FDR q-value < 0.05).

e) Enrichment plots of selected metabolic pathways: Hallmark Oxidative Phosphorylation, Hallmark Glycolysis, GOBP Fatty Acid Metabolic Process, and KEGG Cell Cycle. Comparisons: iNs_D14 vs. iPSCs.

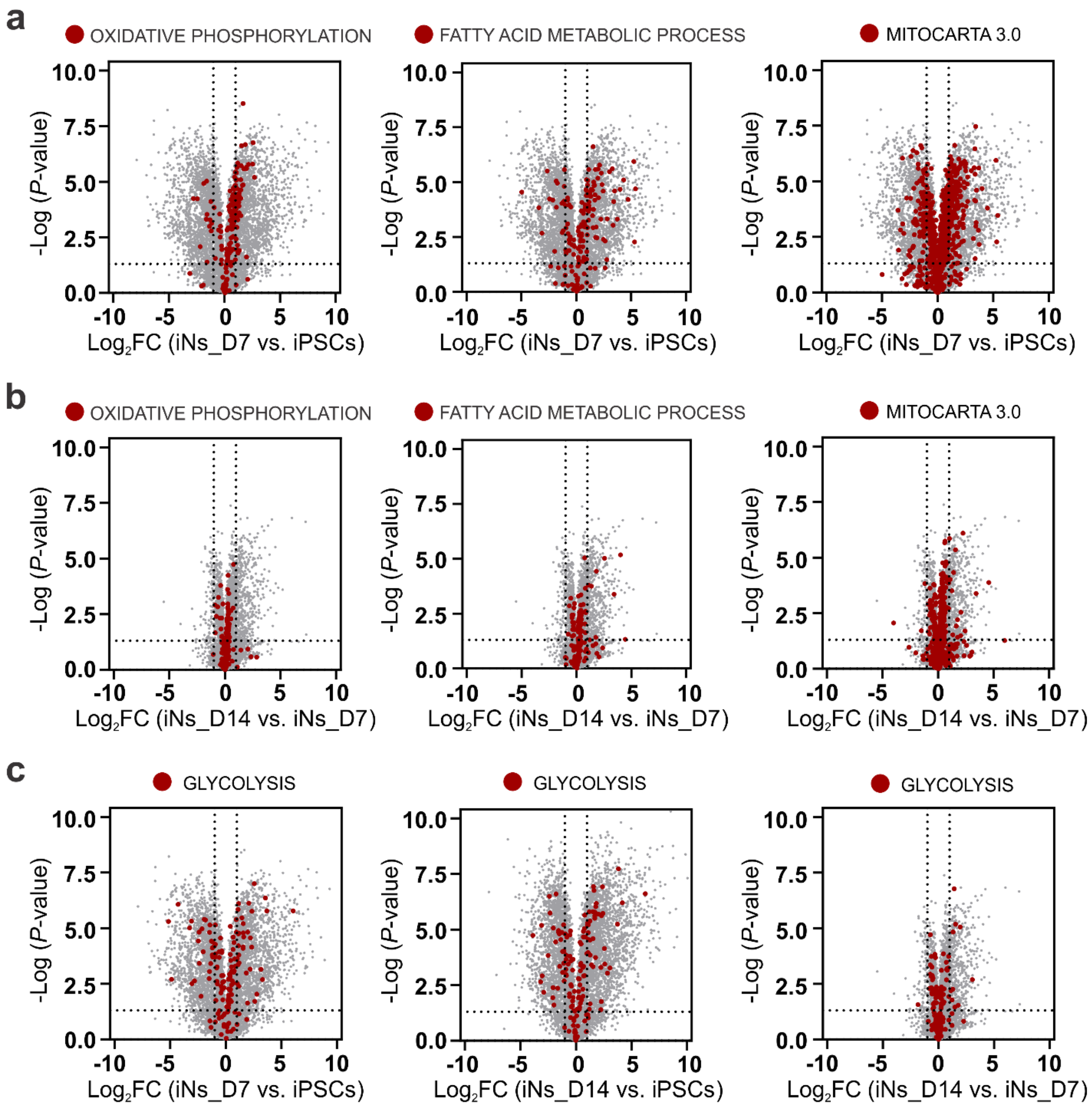

Fig. S3 Differential expression of proteins associated with key metabolic pathways during neuronal differentiation of human iPSCs. Volcano plots show protein expression changes in selected metabolic pathways: Hallmark Oxidative Phosphorylation, GOBP Fatty Acid Metabolic Process, MitoCarta 3.0, and Hallmark Glycolysis, across three comparisons: (a) iNs_D7 vs. iPSCs, (b) iNs_D14 vs. iNs_D7, (c) iNs_D7 vs. iPSCs, iNs_D14 vs. iPSCs, and iNs_D14 vs. iNs_D7. Significantly altered proteins within each pathway are highlighted in red. Analyses are based LFQ-MS; n = 3 (iPSCs), n = 3 (iNs_D7), n = 4 (iNs_D14).

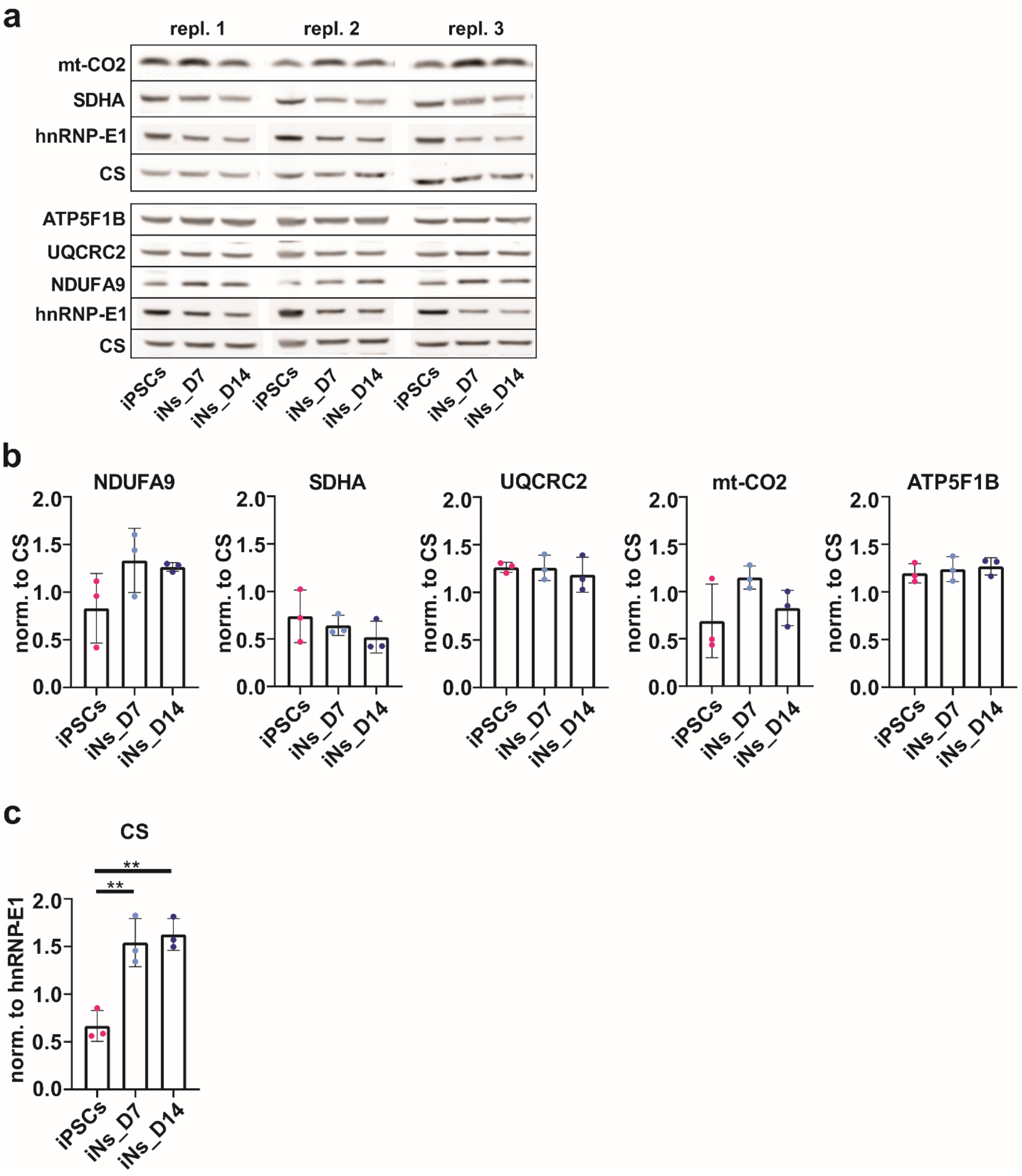
a
repl. 1
repl. 2
repl. 3
mt-CO2
SDHA
hnRNP-E1
CS
ATP5F1B
UQCRC2
NDUFA9
hnRNP-E1
CS
iPSCs
iNs_D7
iNs_D14
b
NDUFA9
SDHA
UQCRC2
mt-CO2
ATP5F1B
norm. to CS
c
CS
norm. to hnRNP-E1
**
**

Fig. S4 Neuronal differentiation is associated with increased mitochondrial respiratory capacity and mitochondrial biogenesis.

a) Western blot analysis of representative subunits of mitochondrial OXPHOS complexes.

b) Western blot quantification of OXPHOS proteins normalized to citrate synthase (CS) as a marker of mitochondrial content.

c) Western blot quantification of CS normalized to hnRNP-E1 as a nuclear marker.

Samples for Western blotting: n = 3 biological replicates per group (repl. 1 – 3). Statistical analysis was performed using one-way ANOVA followed by Tukey's multiple comparisons test. Error bars represent ± SD. **) $p \leq 0.01$; ***) $p \leq 0.001$; ****) $p \leq 0.0001$. NDUFA9 – NADH dehydrogenase [ubiquinone] 1 alpha subcomplex subunit 9, mitochondrial (Complex I); SDHA – Succinate dehydrogenase [ubiquinone] flavoprotein subunit, mitochondrial (Complex II); UQCRC2 – Cytochrome bc1 complex subunit core2, mitochondrial (Complex III); mt-CO2 – Cytochrome c oxidase subunit 2 (Complex IV); ATP5F1B – ATP synthase subunit beta, mitochondrial (Complex V).

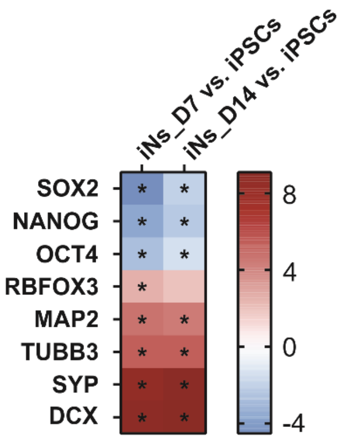

Fig. S5 Proteomic analysis of pluripotency and neuronal markers during differentiation

Label-free quantitative mass spectrometry (LFQ-MS) analysis revealed a decrease in the abundance of pluripotency-associated proteins (NANOG, OCT4) and the pluripotency and neural stem cell marker SOX2, accompanied by an increase in the abundance of neuronal markers (DCX, RBFOX3, SYP, TUBB3, MAP2) during neuronal differentiation. In the graph, the color scale represents $\log_2$ fold change, and asterisks denote significance ($p \leq 0.05$).

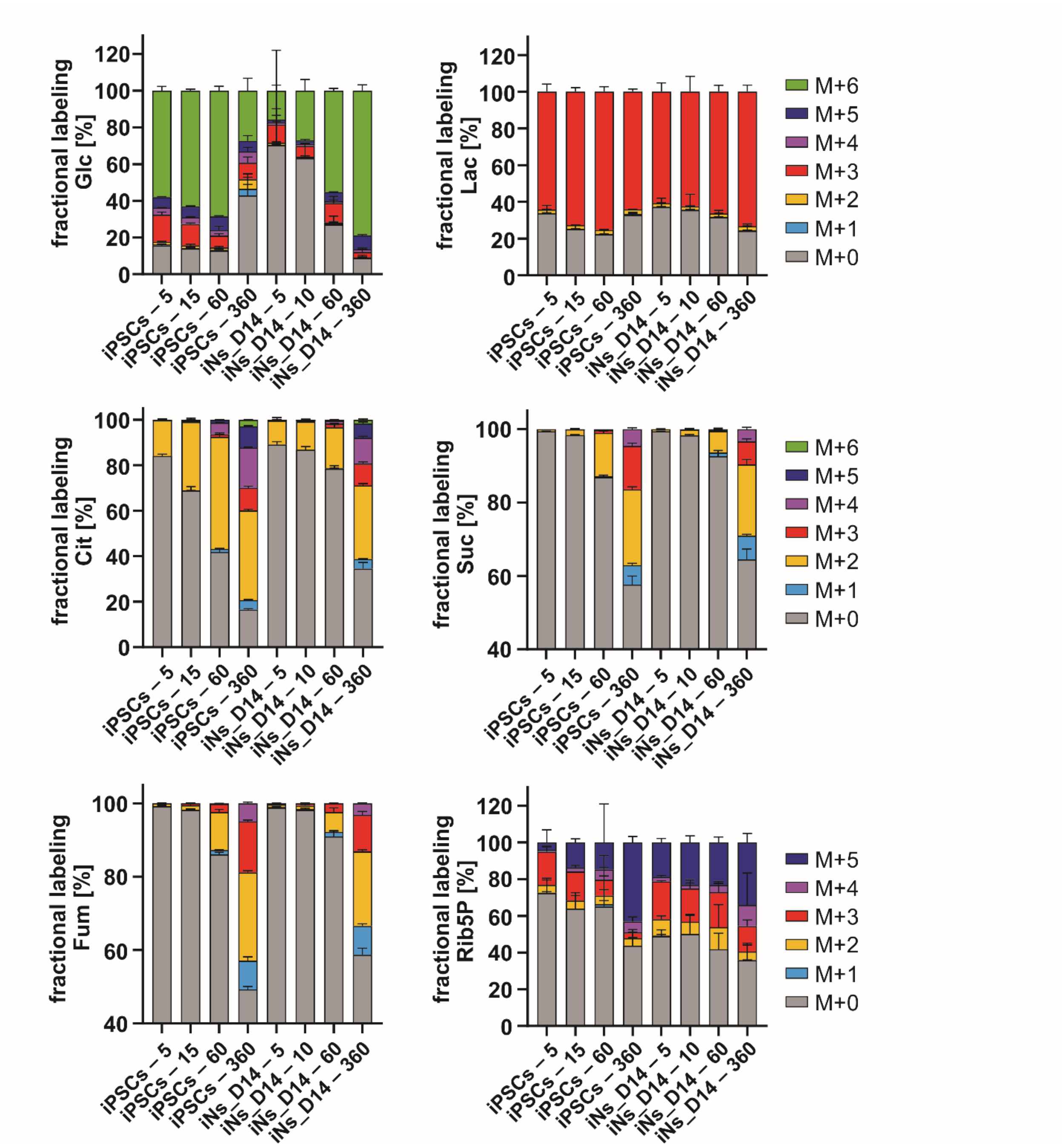

Fig. S6 Time-course of $^{13}C_6$-glucose fractional labeling for glucose (Glc), lactate (Lac), ribose-5-phosphate (Rib5P), and tricarboxylic acid (TCA) cycle metabolites citrate (Cit), succinate (Suc), and fumarate (Fum) in differentiated neurons compared to iPSCs. Timepoints included 5, 15, 60, 360 min. Data represent mean ± SD from three independent experiments.
