# Supplementary material for: Development of Cellular Energy Metabolism During Differentiation of Human iPSCs into Cortical Neurons
Source: Mol Neurobiol. 2025 Nov 13;63(1):37. doi: 10.1007/s12035-025-05284-8 (PMC12615542; doi:10.1007/s12035-025-05284-8)
Supplement: Supplementary file 2 — Supplementary Material 2: Supplementary Methods. (PDF 227 KB) [file 12035_2025_5284_MOESM2_ESM.pdf]

Online Resource 2

Title:
Development of Cellular Energy Metabolism During Differentiation of Human iPSCs Into Cortical Neurons

Authors:
Šárka Danačíková[1,2,3,4], Petr Pecina[5], Alena Pecinová[5], Jan Svoboda[1], David Vondrášek[6], Davide Alessandro Basello[6], Tomáš Čajka[7], Daniel Hadraba[6], Tomáš Mráček[5], Vladimír Kořínek[3], Jakub Otáhal[1,2]*

[1]Department of Pathophysiology, Second Faculty of Medicine, Charles University, Prague, Czech Republic
[2]Laboratory of Developmental Epileptology, Institute of Physiology of the Czech Academy of Sciences, Prague, Czech Republic
[3]Laboratory of Cell and Developmental Biology, Institute of Molecular Genetics of the Czech Academy of Sciences, Prague, Czech Republic
[4]Department of Physiology, Faculty of Science, Charles University, Prague, Czech Republic
[5]Laboratory of Bioenergetics, Institute of Physiology of the Czech Academy of Sciences, Prague, Czech Republic
[6]Laboratory of Biomathematics, Institute of Physiology of the Czech Academy of Sciences, Prague, Czech Republic
[7]Laboratory of Metabolomics, Institute of Physiology of the Czech Academy of Sciences, Prague, Czech Republic

Jakub Otáhal e-mail: jakub.otahal@lfmotol.cuni.cz

Online Resource 2 – Supplementary Methods

Table 1 List of genes and primer sequences for quantitative real-time PCR

Gene (human)	Forward/Reverse	Primer sequence
NANOG	Forward<br>Reverse	ACTCTCCAACATCCTGAACCTC<br>CTTCTGCGTCACACCATTGC
OCT4	Forward<br>Reverse	GTGGAGGAAGCTGACAACAA<br>GCCGGTTACAGAACCACACT
REX1	Forward<br>Reverse	GTGGGAAAGCGTTCGTTGAG<br>CGCTTTCCGCACCCTTC
SOX2	Forward<br>Reverse	CACATGTCCCAGCACTACC<br>CCATGCTGTTTCTTACTCTCCTC
hnRNP-E1	Forward<br>Reverse	ACAACACACCATTTCTCCGC<br>ATGGTGAGTTCATGGGTGGT
DCX	Forward<br>Reverse	AGGAAGATCGGAAGCATGGA<br>AAGTCCTTGTTCTCCCTGGC
RBFOX3	Forward<br>Reverse	TACACGTCTCCAACATCCCC<br>CCCTCTACGATCGTCCCATT
SYP	Forward<br>Reverse	ATCTTCGCCTTTGCCACATG<br>GGCCACGGTGACAAAGAATT
TUBB3	Forward<br>Reverse	GGCCTTTGGACATCTCTTCA<br>ATACTCCTCACGCACCTTGC
MAP2	Forward<br>Reverse	ACTGCAGCTCTGCCTTTAGC<br>ATCGTGGAACTCCATCTTCG
TBP	Forward<br>Reverse	AGTTCTGGGATTGTACCGCA<br>TGTGCACACCATTTTCCCAG

Table 2 Primary and secondary antibodies resource table

Primary antibodies			
RESOURCE	SOURCE	IDENTIFIER	VALIDATION
NDUFA9 antibody	Abcam	Cat# ab14713, RRID:AB_301431	Knock-out validated and literature consistency, see manufacturer information
SDHA antibody	Abcam	Cat# ab14715, RRID:AB_301433	Knock-out validated and literature consistency, see manufacturer information, also KO validated in our previous studies (Ref. 34)
UQCRC2 antibody	Proteintech	Cat# 14742-1-AP, RRID:AB_2241442	Knock-down validated, see manufacturer information
MTCO2 (Cytochrome C oxidase subunit II) antibody	Abcam	Cat# ab110258, RRID:AB_10887758	Knock-out validated in our previous studies (Ref. 34), literature consistency (see manufacturer information)
ATP5F1B (ATPB) antibody	Abcam	Cat# ab14730, RRID:AB_301438	Knock-out validated in our previous studies (Ref. 34), literature consistency (see manufacturer information)
CS (Citrate synthetase) antibody	Abcam	Cat# ab129095, RRID:AB_11143209	No genetic validation but overall literature consistency of the product, see manufacturer information
hnRNPE1 antibody	Cell Signalling Technologies	Cat #8534, RRID:AB_11129258	No genetic validation but overall literature consistency of the product, see manufacturer information

Secondary antibodies		
RESOURCE	SOURCE	IDENTIFIER
Donkey anti-Mouse IgG (H+L) Highly Cross-Adsorbed Secondary Antibody, Alexa Fluor™ 680	Thermo Fisher Scientific	Cat# A10038, RRID:AB_2534014
Donkey anti-Rabbit IgG (H+L) Highly Cross-Adsorbed Secondary Antibody, Alexa Fluor™ 680	Thermo Fisher Scientific	Cat# A10043, RRID:AB_2534018
IRDye 800CW Donkey anti-Mouse IgG	LI-COR Biosciences	Cat# 926-32212, RRID:AB_621847
IRDye 800CW Donkey anti-Rabbit IgG	LI-COR Biosciences	Cat# 926-32213, RRID:AB_621848
