# Supplementary material for: Development of Cellular Energy Metabolism During Differentiation of Human iPSCs into Cortical Neurons
Source: Mol Neurobiol. 2025 Nov 13;63(1):37. doi: 10.1007/s12035-025-05284-8 (PMC12615542; doi:10.1007/s12035-025-05284-8)

Online Resource 4

Title:
Development of Cellular Energy Metabolism During Differentiation of Human iPSCs Into Cortical Neurons

Authors:
Šárka Danačíková[1,2,3,4], Petr Pecina[5], Alena Pecinová[5], Jan Svoboda[1], David Vondrášek[6], Davide Alessandro Basello[6], Tomáš Čajka[7], Daniel Hadraba[6], Tomáš Mráček[5], Vladimír Kořínek[3], Jakub Otáhal[1,2]*

[1]Department of Pathophysiology, Second Faculty of Medicine, Charles University, Prague, Czech Republic
[2]Laboratory of Developmental Epileptology, Institute of Physiology of the Czech Academy of Sciences, Prague, Czech Republic
[3]Laboratory of Cell and Developmental Biology, Institute of Molecular Genetics of the Czech Academy of Sciences, Prague, Czech Republic
[4]Department of Physiology, Faculty of Science, Charles University, Prague, Czech Republic
[5]Laboratory of Bioenergetics, Institute of Physiology of the Czech Academy of Sciences, Prague, Czech Republic
[6]Laboratory of Biomathematics, Institute of Physiology of the Czech Academy of Sciences, Prague, Czech Republic
[7]Laboratory of Metabolomics, Institute of Physiology of the Czech Academy of Sciences, Prague, Czech Republic

Jakub Otáhal e-mail: jakub.otahal@lfmotol.cuni.cz

Online Resource 4 includes two folders:

Folder **Western blot raw image files** contains TIFF files of unprocessed Western blot scans

Folder **Western blot ImageLab quantification reports** contains quntification reports in PDF format

Original Western blot images and quantifications for Fig. 4e-f and Online Resource 1 – Fig. S4

All WB images in the manuscript come from multiple antibody incubation of two PVDF membranes (internal lab codes 7056, 7057) from gels with identical sample loading:

1. Molecular weight marker
2. iPSCs repl1
3. iNs_D7 repl1
4. iNs_D14 repl1
5. sample not relevant for the study
6. iPSCs repl2
7. iNS_D7 repl2
8. iNS_D14 repl2
9. sample not relevant for the study
10. iPSCs repl3
11. iNS_D7 repl3
12. iNS_D14 repl3
13. sample not relevant for the study
14. Molecular weight marker
15. Mixed sample of the 3 replicates of iPSCs used as internal standard

Membrane no. 7056

a

1. Primary antibody xMT-CO2,
secondary xmouse IgG
scanned in 700 nm channel

2. Primary antibody xSDHA,
secondary xmouse IgG
scanned in 700 nm channel

Raw image

SDHA

MT-CO2

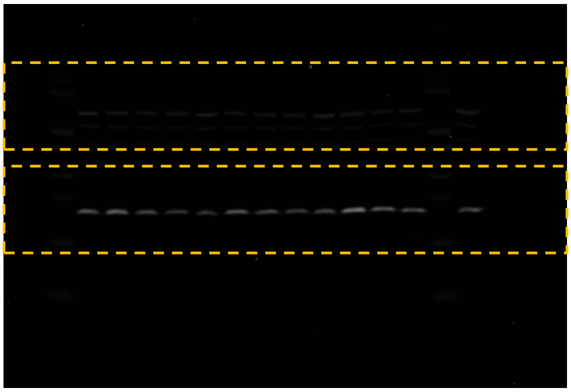

Screenshot from ImageLab quantification

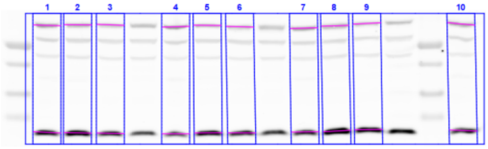

b

3. Primary antibody xhnRNPE1,
secondary xrabbit IgG scanned in
800nm channel

4. Primary antibody xCS,
secondary xrabbit IgG scanned in
800nm channel

Raw image

CS

hnRNPE1

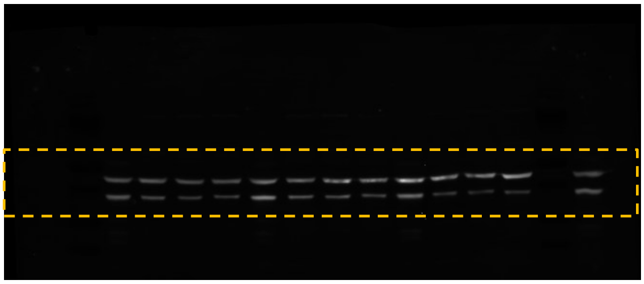

Screenshot from ImageLab quantification

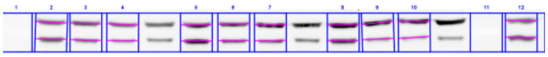

Membrane no. 7057

c

1. Mixture of primary antibody xSDHA, xATP5F1B, xUQCRC2, xNDUFA9,
secondary xmouse IgG
scanned in 700 nm channel

Raw image | Screenshot from ImageLab quantification

SDHA
ATP5F1B
UQCRC2
NDUFA9

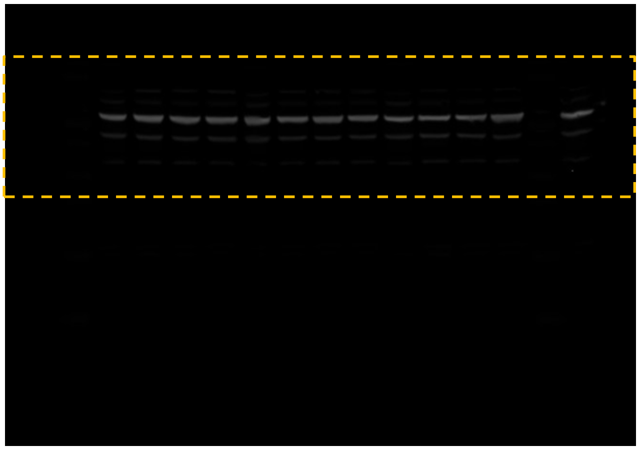

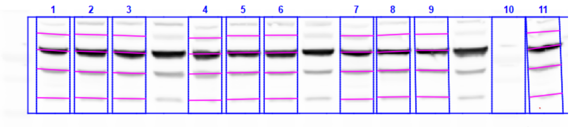

d

2. Primary antibody xhnRNPE1,
secondary xrabbit IgG
scanned in 800nm channel

3. Primary antibody xCS,
secondary xrabbit IgG
scanned in 800nm channel

Raw image | Screenshot from ImageLab quantification

CS
hnRNPE1

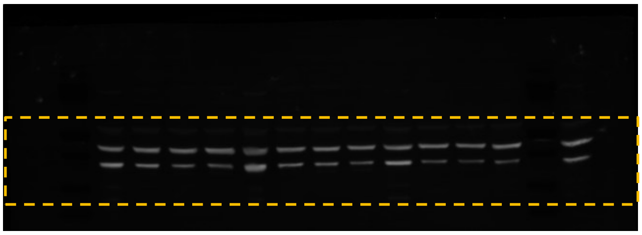

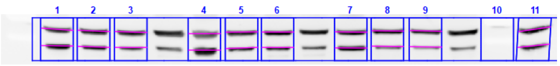

Supplement: Supplementary file 4 — Supplementary Material 4: Zipped folder containing uncropped Western blot images, quantification reports, and a descriptive summary file. (ZIP 4.70 MB) [file 12035_2025_5284_MOESM4_ESM.zip › Online Resource 4/Western blot files description.pdf]
