# Supplementary material for: Development of Cellular Energy Metabolism During Differentiation of Human iPSCs into Cortical Neurons
Source: Mol Neurobiol. 2025 Nov 13;63(1):37. doi: 10.1007/s12035-025-05284-8 (PMC12615542; doi:10.1007/s12035-025-05284-8)
Supplement: Supplementary file 4 — Supplementary Material 4: Zipped folder containing uncropped Western blot images, quantification reports, and a descriptive summary file. (ZIP 4.70 MB) [file 12035_2025_5284_MOESM4_ESM.zip › Online Resource 4/Western blot ImageLab quantification reports/7056_a_quantification_report.pdf]

# Image Report: 7056_SDHA_Cox2_quant2

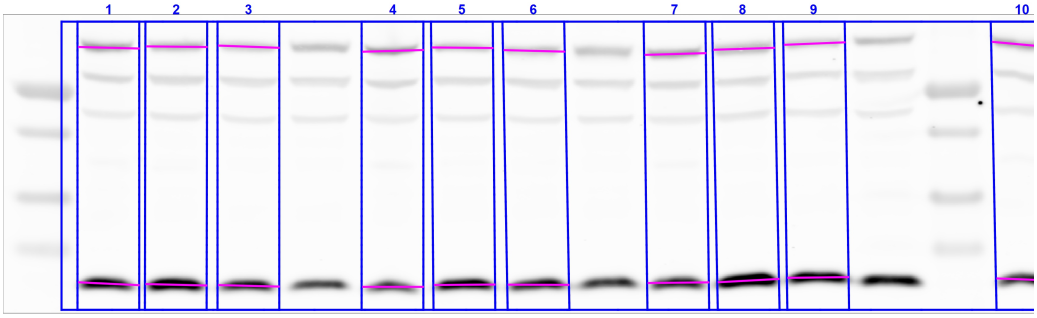


**C:\Users\petr.pecina\Desktop\Neurodiferenciace projekt\Quant\reanalysis without D21\7056_SDHA_Cox2_quant2.scn**

## Acquisition Information

| | |
|---|---|
| Program | 2.1.12 |
| Imager | LI-COR |

## Image Information

| | |
|---|---|
| Acquisition Date | 24/03/2023 3:53:03 PM |
| User Name | Knězů Michal |
| Image Area (mm) | X: 86.8 Y: 24.2 |
| Pixel Size (µm) | X: 84.7 Y: 84.7 |
| Data Range (Int) | 16 - 29963 |

## Analysis Settings

| | |
|---|---|
| Detection | Lane detection:<br>Manually created lanes<br><br>Band detection:<br>Automatically detected bands with sensitivity: Low<br>Manually adjusted bands<br><br>Lane Background Subtraction:<br>Lane background subtracted with disk size: 0.1<br><br>Lane width: 5.00 mm |

## Lane Statistics

| Lane No. | Adj. Total Band Vol. (Int) | Total Band Vol. (Int) | Adj. Total Lane Vol. (Int) | Total Lane Vol. (Int) | Bkgd. Vol. (Int) | Norm. Factor |
|---|---|---|---|---|---|---|
| 1 | 10,513,741 | 10,968,513 | 12,039,363 | 15,232,266 | 3,192,903 | N/A |
| 2 | 12,451,183 | 12,827,485 | 14,224,310 | 17,111,357 | 2,887,047 | N/A |
| 3 | 8,218,936 | 8,547,212 | 9,701,665 | 12,393,658 | 2,691,993 | N/A |
| 4 | 6,852,201 | 7,252,929 | 8,385,788 | 11,535,267 | 3,149,479 | N/A |
| 5 | 10,468,724 | 10,806,086 | 12,049,452 | 14,862,690 | 2,813,238 | N/A |
| 6 | 8,849,941 | 9,196,743 | 10,356,093 | 13,204,908 | 2,848,815 | N/A |
| 7 | 9,311,557 | 9,742,611 | 10,917,065 | 14,125,190 | 3,208,125 | N/A |

| | | | | | | |
|---|---|---|---|---|---|---|
| 8 | 16,063,281 | 16,499,881 | 17,743,778 | 20,722,039 | 2,978,261 | N/A |
| 9 | 11,080,318 | 11,464,703 | 12,513,782 | 15,369,382 | 2,855,600 | N/A |
| 10 | 9,739,071 | 10,191,011 | 11,354,314 | 14,603,444 | 3,249,130 | N/A |

## Lane And Band Analysis

### Lane 1

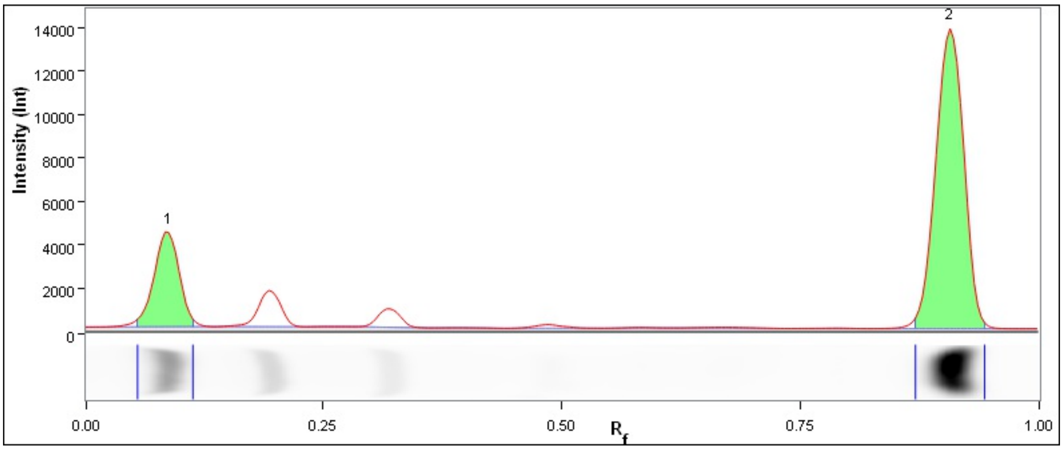


| Band No. | Band Label | Mol. Wt. (KDa) | Relative Front | Adj. Volume (Int) | Volume (Int) | Abs. Quant. | Rel. Quant. | Band % | Lane % |
|---|---|---|---|---|---|---|---|---|---|
| 1 | | N/A | 0.091 | 2,342,595 | 2,602,785 | N/A | N/A | 22.3 | 19.5 |
| 2 | | N/A | 0.909 | 8,171,146 | 8,365,728 | N/A | N/A | 77.7 | 67.9 |

| | |
|---|---|
| Band Detection | Automatically detected bands with sensitivity: Low |
| Lane Background | Lane background subtracted with disk size: 0.1 |
| Lane Width | 5.00 mm |

### Lane 2

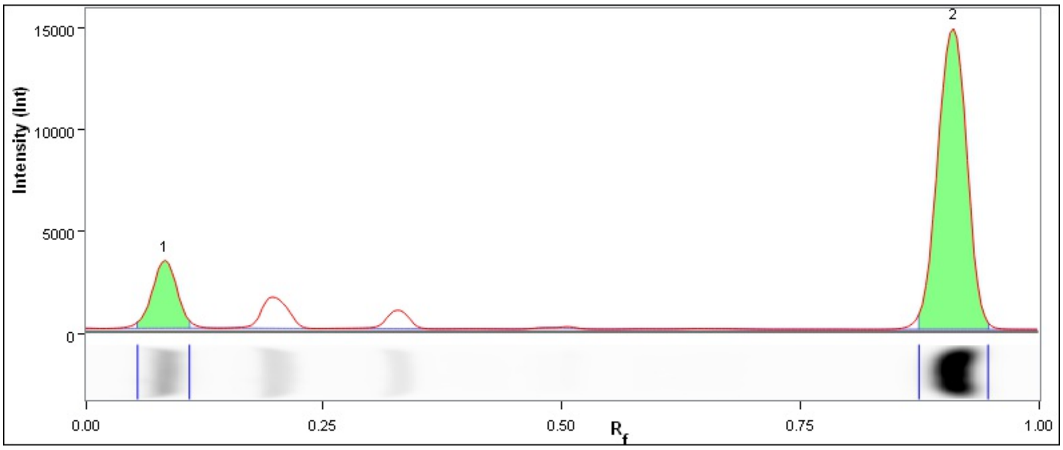


| Band No. | Band Label | Mol. Wt. (KDa) | Relative Front | Adj. Volume (Int) | Volume (Int) | Abs. Quant. | Rel. Quant. | Band % | Lane % |
|---|---|---|---|---|---|---|---|---|---|
| 1 | | N/A | 0.087 | 1,993,492 | 2,192,676 | N/A | N/A | 16.0 | 14.0 |
| 2 | | N/A | 0.913 | 10,457,691 | 10,634,809 | N/A | N/A | 84.0 | 73.5 |

| | |
|---|---|
| Band Detection | Automatically detected bands with sensitivity: Low |

| | |
|---|---|
| Lane Background | Lane background subtracted with disk size: 0.1 |
| Lane Width | 5.00 mm |

## Lane 3

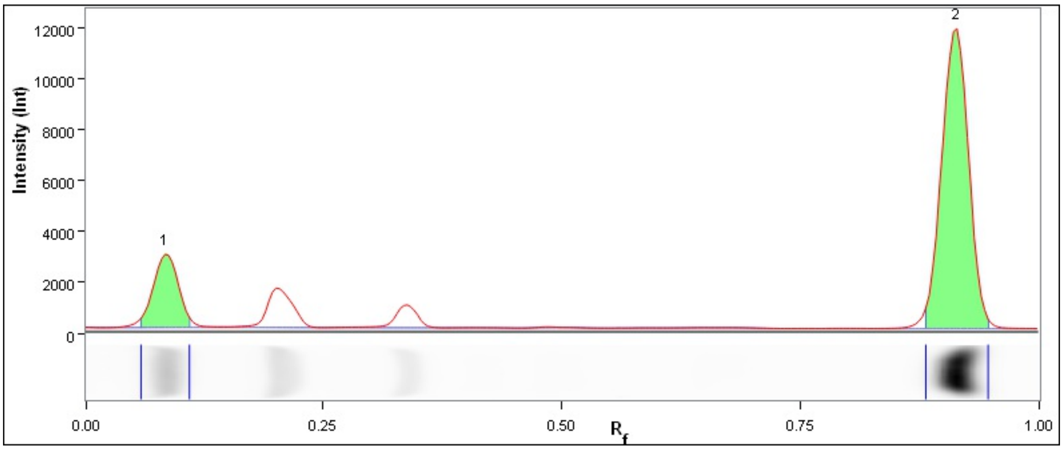


| Band No. | Band Label | Mol. Wt. (KDa) | Relative Front | Adj. Volume (Int) | Volume (Int) | Abs. Quant. | Rel. Quant. | Band % | Lane % |
|---|---|---|---|---|---|---|---|---|---|
| 1 | | N/A | 0.087 | 1,521,315 | 1,690,999 | N/A | N/A | 18.5 | 15.7 |
| 2 | | N/A | 0.917 | 6,697,621 | 6,856,213 | N/A | N/A | 81.5 | 69.0 |

| | |
|---|---|
| Band Detection | Automatically detected bands with sensitivity: Low |
| Lane Background | Lane background subtracted with disk size: 0.1 |
| Lane Width | 5.00 mm |

## Lane 4

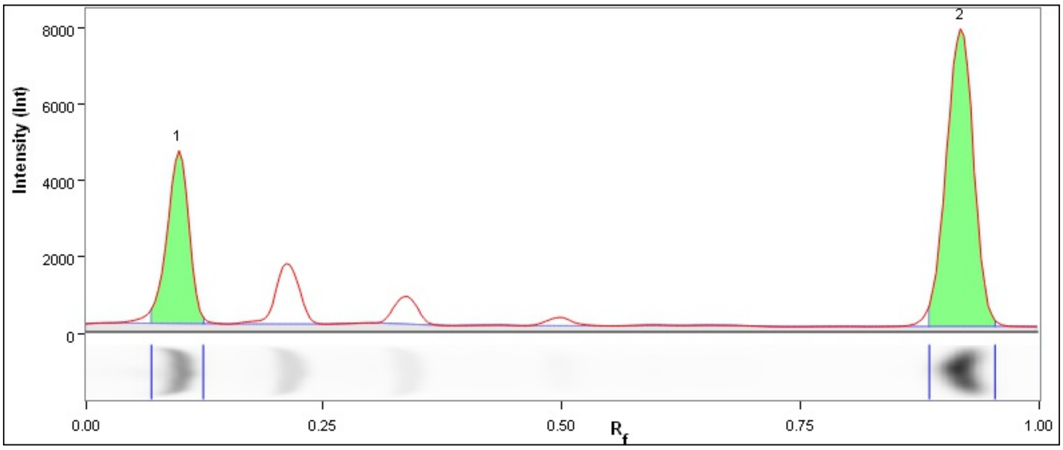


| Band No. | Band Label | Mol. Wt. (KDa) | Relative Front | Adj. Volume (Int) | Volume (Int) | Abs. Quant. | Rel. Quant. | Band % | Lane % |
|---|---|---|---|---|---|---|---|---|---|
| 1 | | N/A | 0.101 | 2,172,970 | 2,401,300 | N/A | N/A | 31.7 | 25.9 |
| 2 | | N/A | 0.920 | 4,679,231 | 4,851,629 | N/A | N/A | 68.3 | 55.8 |

| | |
|---|---|
| Band Detection | Automatically detected bands with sensitivity: Low |
| Lane Background | Lane background subtracted with disk size: 0.1 |
| Lane Width | 5.00 mm |

## Lane 5

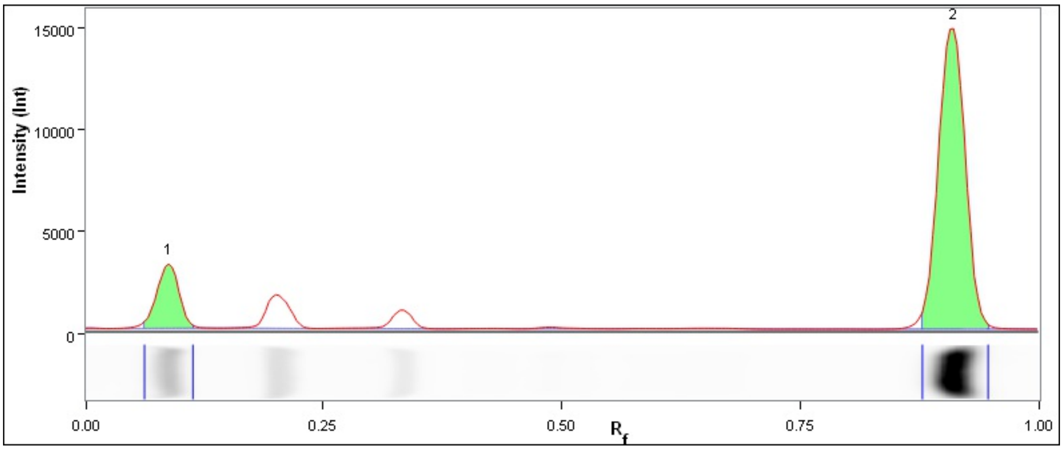


| Band No. | Band Label | Mol. Wt. (KDa) | Relative Front | Adj. Volume (Int) | Volume (Int) | Abs. Quant. | Rel. Quant. | Band % | Lane % |
|---|---|---|---|---|---|---|---|---|---|
| 1 | | N/A | 0.091 | 1,568,279 | 1,739,438 | N/A | N/A | 15.0 | 13.0 |
| 2 | | N/A | 0.913 | 8,900,445 | 9,066,648 | N/A | N/A | 85.0 | 73.9 |

| | |
|---|---|
| Band Detection | Automatically detected bands with sensitivity: Low |
| Lane Background | Lane background subtracted with disk size: 0.1 |
| Lane Width | 5.00 mm |

## Lane 6

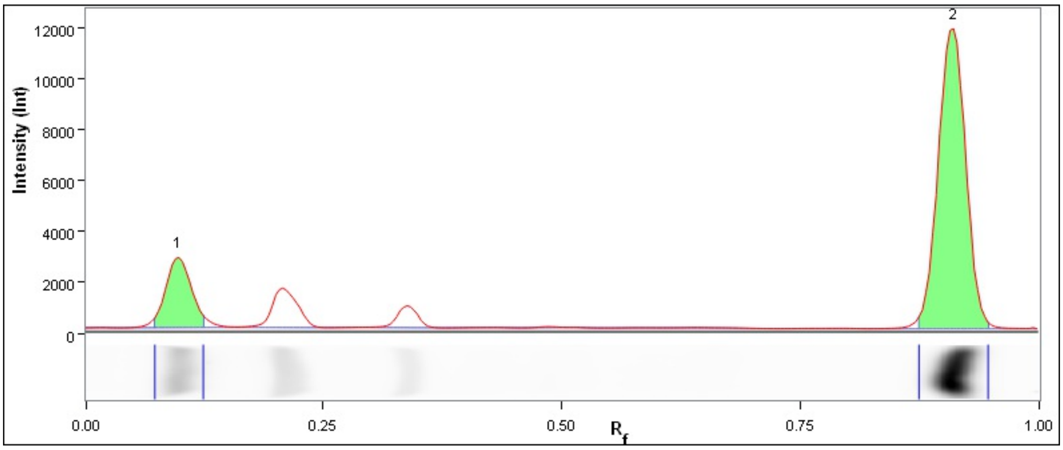


| Band No. | Band Label | Mol. Wt. (KDa) | Relative Front | Adj. Volume (Int) | Volume (Int) | Abs. Quant. | Rel. Quant. | Band % | Lane % |
|---|---|---|---|---|---|---|---|---|---|
| 1 | | N/A | 0.101 | 1,568,869 | 1,742,329 | N/A | N/A | 17.7 | 15.1 |
| 2 | | N/A | 0.913 | 7,281,072 | 7,454,414 | N/A | N/A | 82.3 | 70.3 |

| | |
|---|---|
| Band Detection | Automatically detected bands with sensitivity: Low |
| Lane Background | Lane background subtracted with disk size: 0.1 |
| Lane Width | 5.00 mm |

## Lane 7

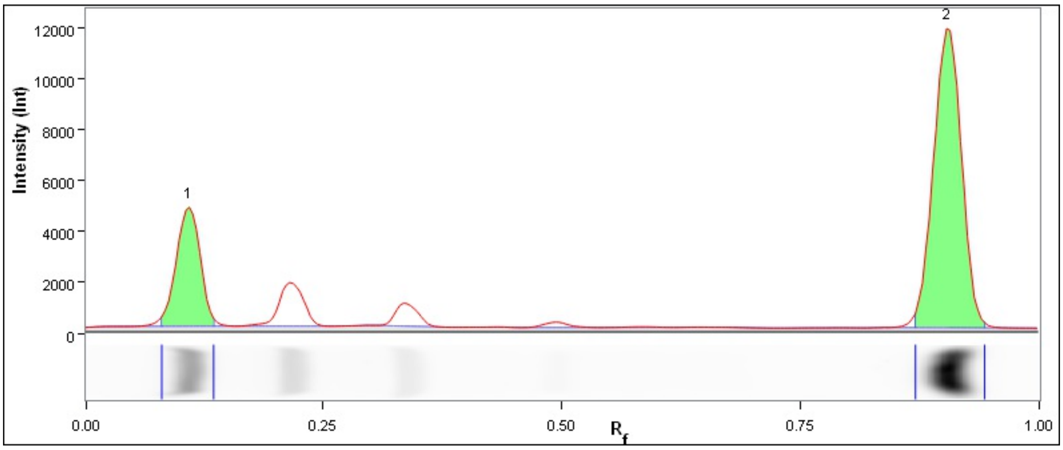


| Band No. | Band Label | Mol. Wt. (KDa) | Relative Front | Adj. Volume (Int) | Volume (Int) | Abs. Quant. | Rel. Quant. | Band % | Lane % |
|---|---|---|---|---|---|---|---|---|---|
| 1 | | N/A | 0.112 | 2,381,240 | 2,615,706 | N/A | N/A | 25.6 | 21.8 |
| 2 | | N/A | 0.906 | 6,930,317 | 7,126,905 | N/A | N/A | 74.4 | 63.5 |

| | |
|---|---|
| Band Detection | Automatically detected bands with sensitivity: Low |
| Lane Background | Lane background subtracted with disk size: 0.1 |
| Lane Width | 5.00 mm |

## Lane 8

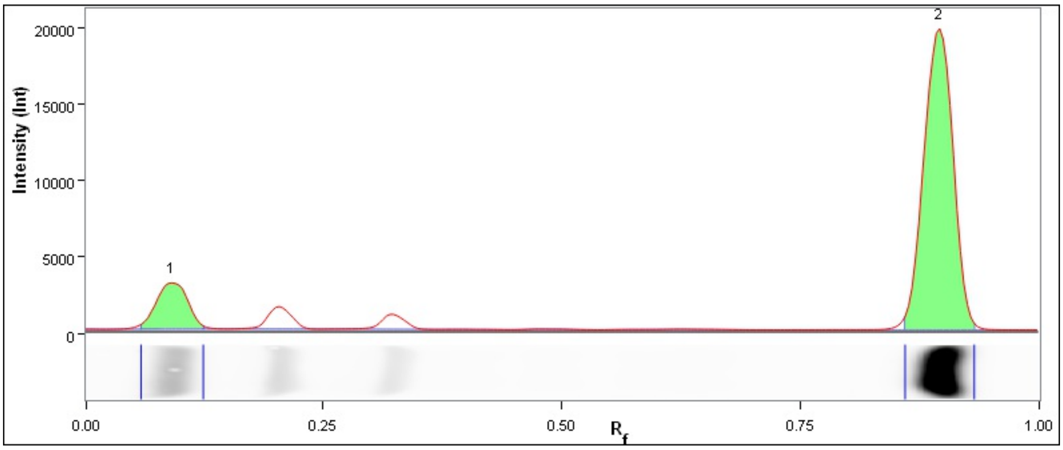


| Band No. | Band Label | Mol. Wt. (KDa) | Relative Front | Adj. Volume (Int) | Volume (Int) | Abs. Quant. | Rel. Quant. | Band % | Lane % |
|---|---|---|---|---|---|---|---|---|---|
| 1 | | N/A | 0.094 | 2,297,991 | 2,537,885 | N/A | N/A | 14.3 | 13.0 |
| 2 | | N/A | 0.899 | 13,765,290 | 13,961,996 | N/A | N/A | 85.7 | 77.6 |

| | |
|---|---|
| Band Detection | Automatically detected bands with sensitivity: Low |
| Lane Background | Lane background subtracted with disk size: 0.1 |
| Lane Width | 5.00 mm |

## Lane 9

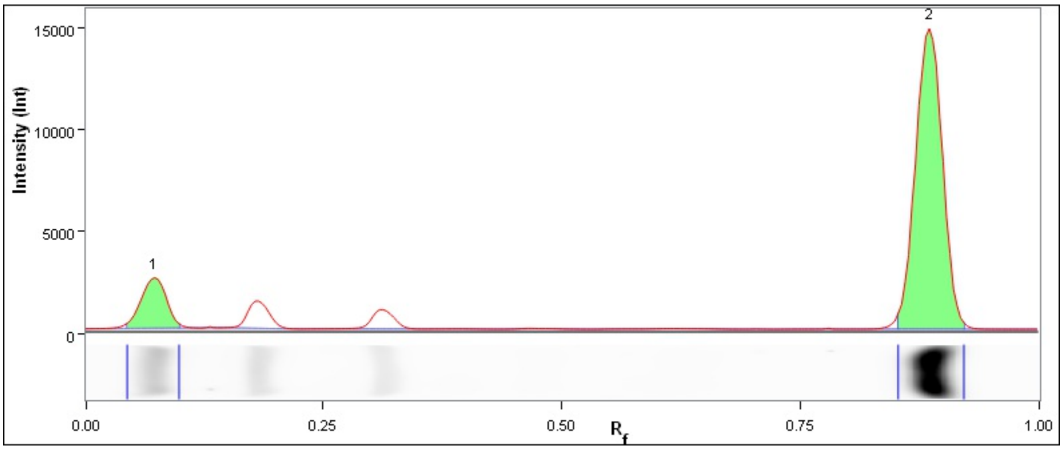


| Band No. | Band Label | Mol. Wt. (KDa) | Relative Front | Adj. Volume (Int) | Volume (Int) | Abs. Quant. | Rel. Quant. | Band % | Lane % |
|---|---|---|---|---|---|---|---|---|---|
| 1 | | N/A | 0.076 | 1,513,173 | 1,713,360 | N/A | N/A | 13.7 | 12.1 |
| 2 | | N/A | 0.888 | 9,567,145 | 9,751,343 | N/A | N/A | 86.3 | 76.5 |

| | |
|---|---|
| Band Detection | Automatically detected bands with sensitivity: Low |
| Lane Background | Lane background subtracted with disk size: 0.1 |
| Lane Width | 5.00 mm |

## Lane 10

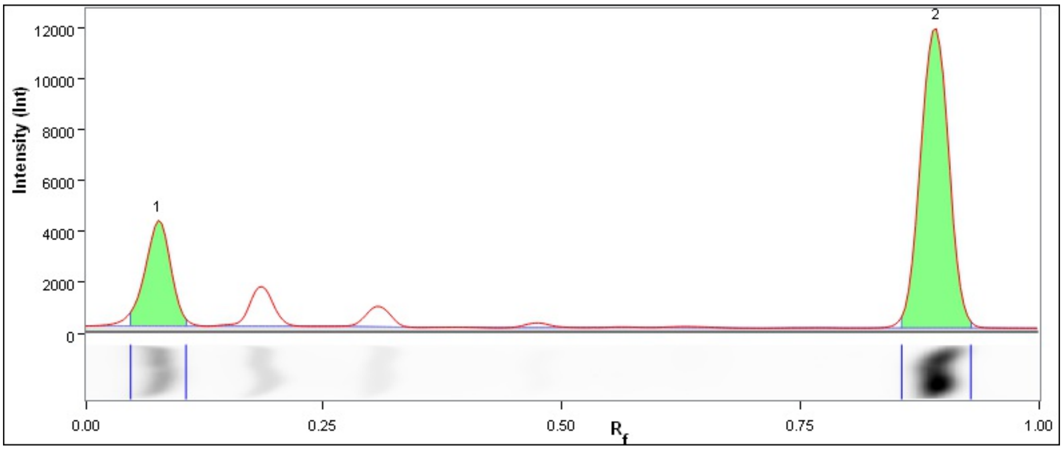


| Band No. | Band Label | Mol. Wt. (KDa) | Relative Front | Adj. Volume (Int) | Volume (Int) | Abs. Quant. | Rel. Quant. | Band % | Lane % |
|---|---|---|---|---|---|---|---|---|---|
| 1 | | N/A | 0.080 | 2,353,746 | 2,611,458 | N/A | N/A | 24.2 | 20.7 |
| 2 | | N/A | 0.895 | 7,385,325 | 7,579,553 | N/A | N/A | 75.8 | 65.0 |

| | |
|---|---|
| Band Detection | Automatically detected bands with sensitivity: Low |
| Lane Background | Lane background subtracted with disk size: 0.1 |
| Lane Width | 5.00 mm |
