# Supplementary material for: Development of Cellular Energy Metabolism During Differentiation of Human iPSCs into Cortical Neurons
Source: Mol Neurobiol. 2025 Nov 13;63(1):37. doi: 10.1007/s12035-025-05284-8 (PMC12615542; doi:10.1007/s12035-025-05284-8)
Supplement: Supplementary file 4 — Supplementary Material 4: Zipped folder containing uncropped Western blot images, quantification reports, and a descriptive summary file. (ZIP 4.70 MB) [file 12035_2025_5284_MOESM4_ESM.zip › Online Resource 4/Western blot ImageLab quantification reports/7056_b_quantification_report.pdf]

Image Report: 7056_ctrsynth_hnRNPe1_quant2

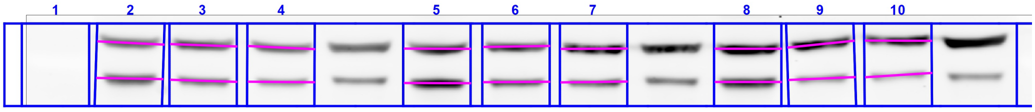

C:\Users\petr.pecina\Desktop\Neurodiferenciace projekt\Quant\reanalysis without D21\7056_ctrsynth_hnRNPe1_quant2.scn

Acquisition Information

Program	2.1.12
Imager	LI-COR

Image Information

Acquisition Date	28/03/2023 2:54:48 PM
User Name	Knězů Michal
Image Area (mm)	X: 85.2 Y: 6.8
Pixel Size (µm)	X: 84.7 Y: 84.7
Data Range (Int)	329 - 56518

Analysis Settings

Detection	Lane detection:<br>Manually created lanes<br><br>Band detection:<br>Automatically detected bands with sensitivity: Low<br>Manually adjusted bands<br><br>Lane Background Subtraction:<br>Lane background subtracted with disk size: 0.1<br><br>Lane width: 5.00 mm

Lane Statistics

Lane No.	Adj. Total Band Vol. (Int)	Total Band Vol. (Int)	Adj. Total Lane Vol. (Int)	Total Lane Vol. (Int)	Bkgd. Vol. (Int)	Norm. Factor
1	N/A	N/A	157,884	3,052,247	2,894,363	N/A
2	12,925,543	14,942,399	13,351,169	17,754,339	4,403,170	N/A
3	11,512,788	13,273,820	11,892,512	16,123,933	4,231,421	N/A
4	8,917,850	10,620,472	9,190,253	13,424,565	4,234,312	N/A
5	17,856,763	20,134,576	18,420,567	23,389,134	4,968,567	N/A
6	12,555,023	14,391,693	13,039,472	17,781,656	4,742,184	N/A
7	14,729,468	16,640,124	15,222,236	19,966,780	4,744,544	N/A
8	22,873,061	25,189,637	23,419,401	28,342,656	4,923,255	N/A
9	13,854,852	15,706,213	14,252,866	18,786,131	4,533,265	N/A
10	12,392,242	14,348,210	12,718,158	17,596,455	4,878,297	N/A
11	N/A	N/A	393,589	3,898,484	3,504,895	N/A
12	13,935,623	16,311,789	14,295,936	19,297,071	5,001,135	N/A

Lane And Band Analysis

Lane 1

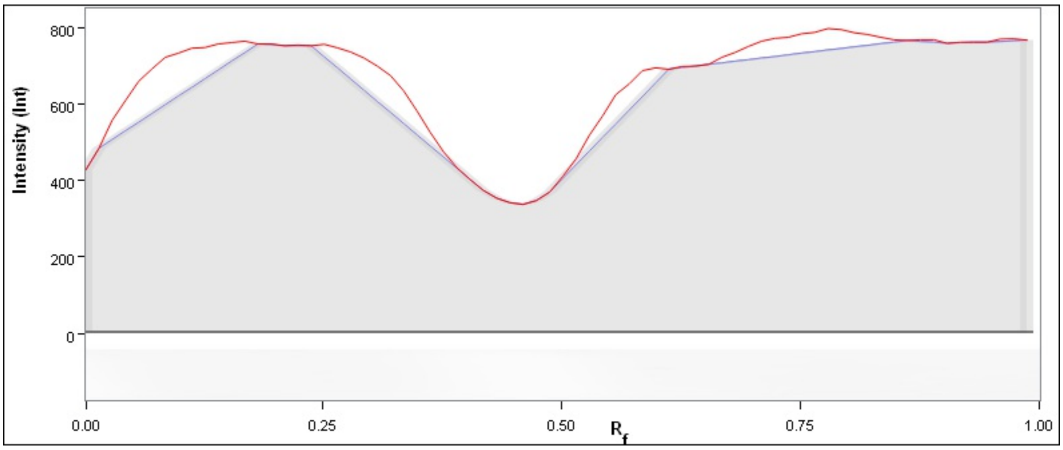

Band No.	Band Label	Mol. Wt. (KDa)	Relative Front	Adj. Volume (Int)	Volume (Int)	Abs. Quant.	Rel. Quant.	Band %	Lane %

Band Detection	Automatically detected bands with sensitivity: Low
Lane Background	Lane background subtracted with disk size: 0.1
Lane Width	5.00 mm

Lane 2

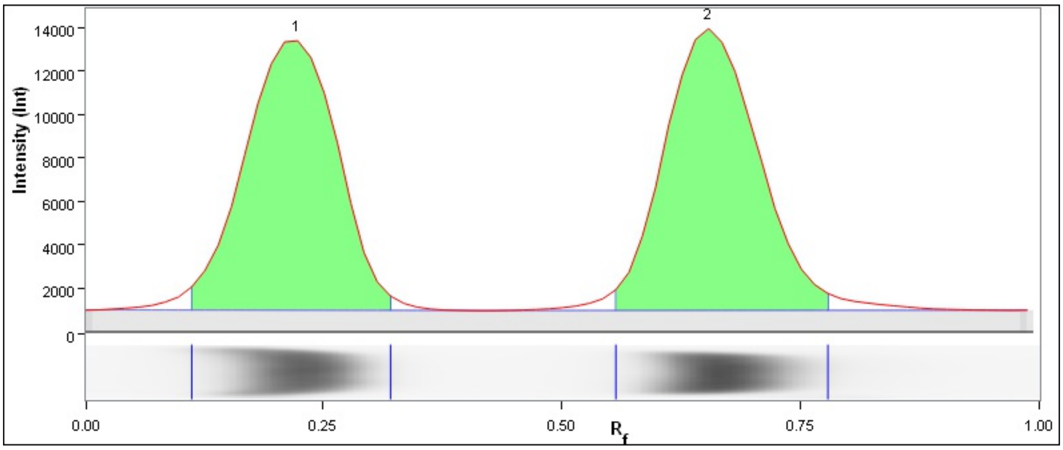

Band No.	Band Label	Mol. Wt. (KDa)	Relative Front	Adj. Volume (Int)	Volume (Int)	Abs. Quant.	Rel. Quant.	Band %	Lane %
1		N/A	0.236	6,292,586	7,275,821	N/A	N/A	48.7	47.1
2		N/A	0.667	6,632,957	7,666,578	N/A	N/A	51.3	49.7

Band Detection	Automatically detected bands with sensitivity: Low
Lane Background	Lane background subtracted with disk size: 0.1
Lane Width	5.00 mm

Lane 3

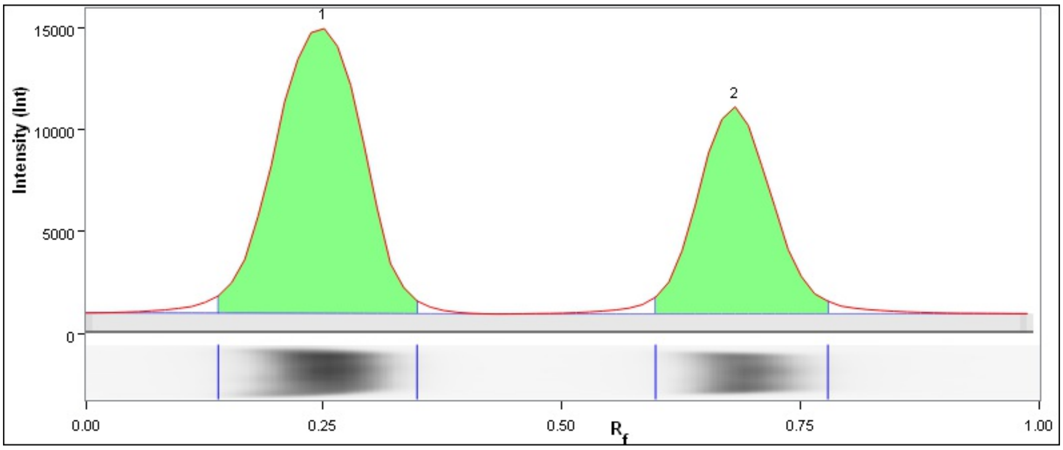

Band No.	Band Label	Mol. Wt. (KDa)	Relative Front	Adj. Volume (Int)	Volume (Int)	Abs. Quant.	Rel. Quant.	Band %	Lane %
1		N/A	0.264	7,159,060	8,107,308	N/A	N/A	62.2	60.2
2		N/A	0.694	4,353,728	5,166,512	N/A	N/A	37.8	36.6

Band Detection	Automatically detected bands with sensitivity: Low
Lane Background	Lane background subtracted with disk size: 0.1
Lane Width	5.00 mm

Lane 4

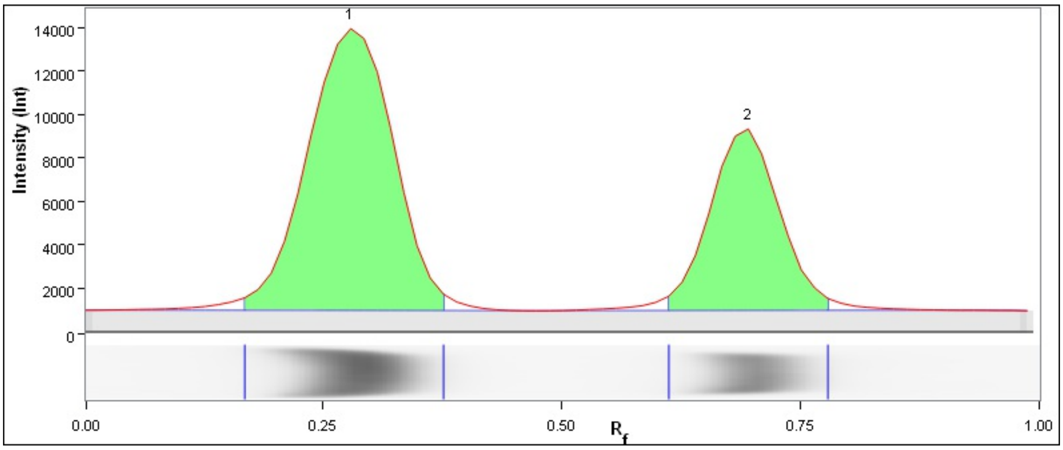

Band No.	Band Label	Mol. Wt. (KDa)	Relative Front	Adj. Volume (Int)	Volume (Int)	Abs. Quant.	Rel. Quant.	Band %	Lane %
1		N/A	0.292	5,864,895	6,803,998	N/A	N/A	65.8	63.8
2		N/A	0.708	3,052,955	3,816,474	N/A	N/A	34.2	33.2

Band Detection	Automatically detected bands with sensitivity: Low
Lane Background	Lane background subtracted with disk size: 0.1
Lane Width	5.00 mm

Lane 5

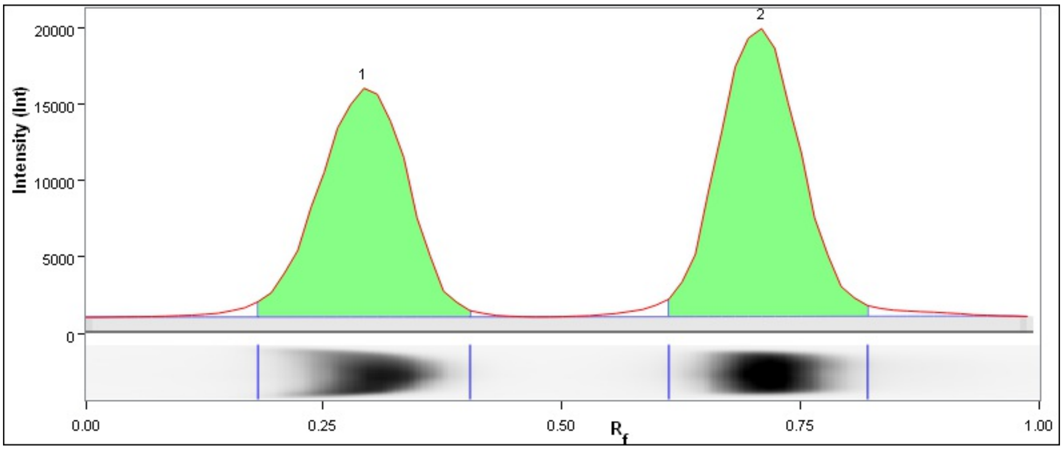

Band No.	Band Label	Mol. Wt. (KDa)	Relative Front	Adj. Volume (Int)	Volume (Int)	Abs. Quant.	Rel. Quant.	Band %	Lane %
1		N/A	0.306	8,267,788	9,422,359	N/A	N/A	46.3	44.9
2		N/A	0.722	9,588,975	10,712,217	N/A	N/A	53.7	52.1

Band Detection	Automatically detected bands with sensitivity: Low
Lane Background	Lane background subtracted with disk size: 0.1
Lane Width	5.00 mm

Lane 6

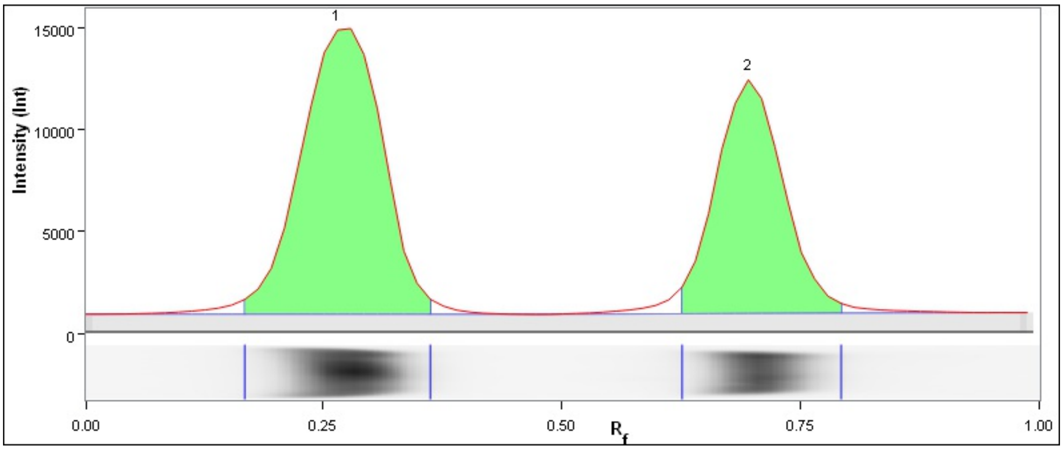

Band No.	Band Label	Mol. Wt. (KDa)	Relative Front	Adj. Volume (Int)	Volume (Int)	Abs. Quant.	Rel. Quant.	Band %	Lane %
1		N/A	0.278	7,516,187	8,478,654	N/A	N/A	59.9	57.6
2		N/A	0.708	5,038,836	5,913,039	N/A	N/A	40.1	38.6

Band Detection	Automatically detected bands with sensitivity: Low
Lane Background	Lane background subtracted with disk size: 0.1
Lane Width	5.00 mm

Lane 7

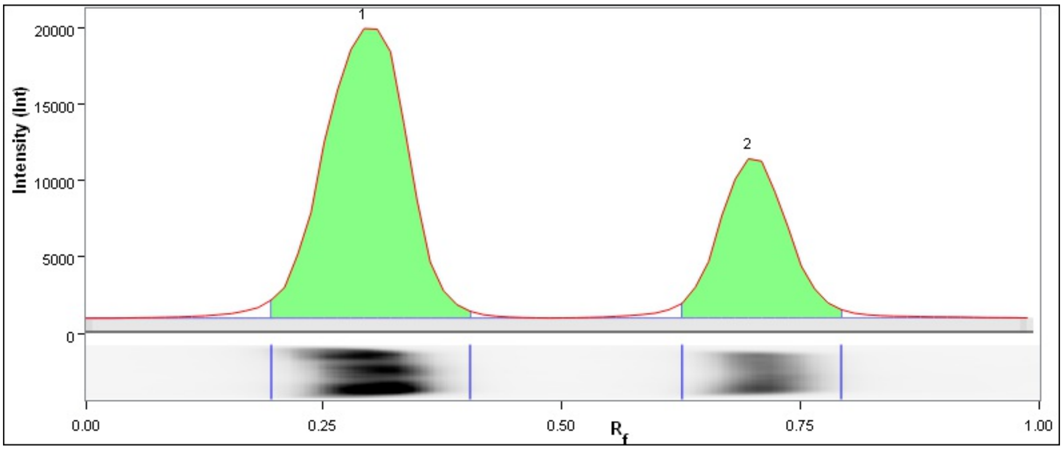

Band No.	Band Label	Mol. Wt. (KDa)	Relative Front	Adj. Volume (Int)	Volume (Int)	Abs. Quant.	Rel. Quant.	Band %	Lane %
1		N/A	0.306	10,135,138	11,183,155	N/A	N/A	68.8	66.6
2		N/A	0.708	4,594,330	5,456,969	N/A	N/A	31.2	30.2

Band Detection	Automatically detected bands with sensitivity: Low
Lane Background	Lane background subtracted with disk size: 0.1
Lane Width	5.00 mm

Lane 8

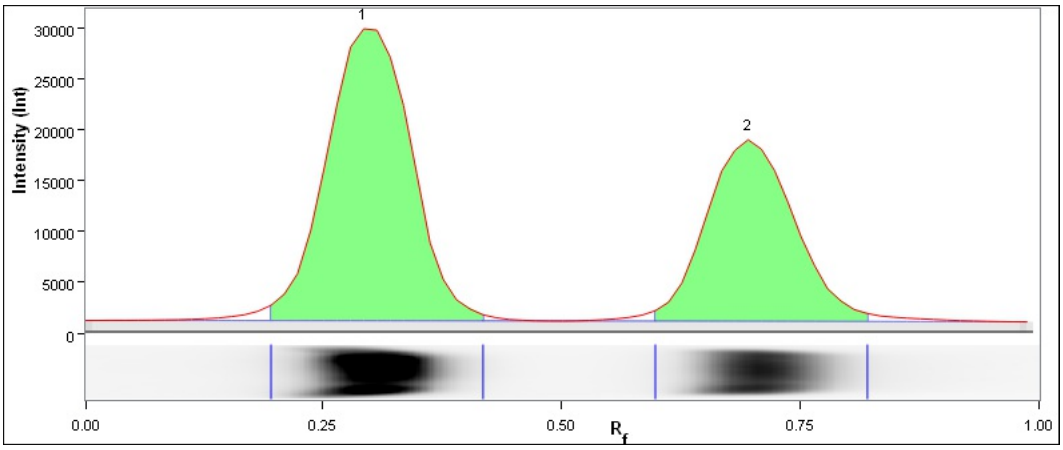

Band No.	Band Label	Mol. Wt. (KDa)	Relative Front	Adj. Volume (Int)	Volume (Int)	Abs. Quant.	Rel. Quant.	Band %	Lane %
1		N/A	0.306	13,904,530	15,096,448	N/A	N/A	60.8	59.4
2		N/A	0.708	8,968,531	10,093,189	N/A	N/A	39.2	38.3

Band Detection	Automatically detected bands with sensitivity: Low
Lane Background	Lane background subtracted with disk size: 0.1
Lane Width	5.00 mm

Lane 9

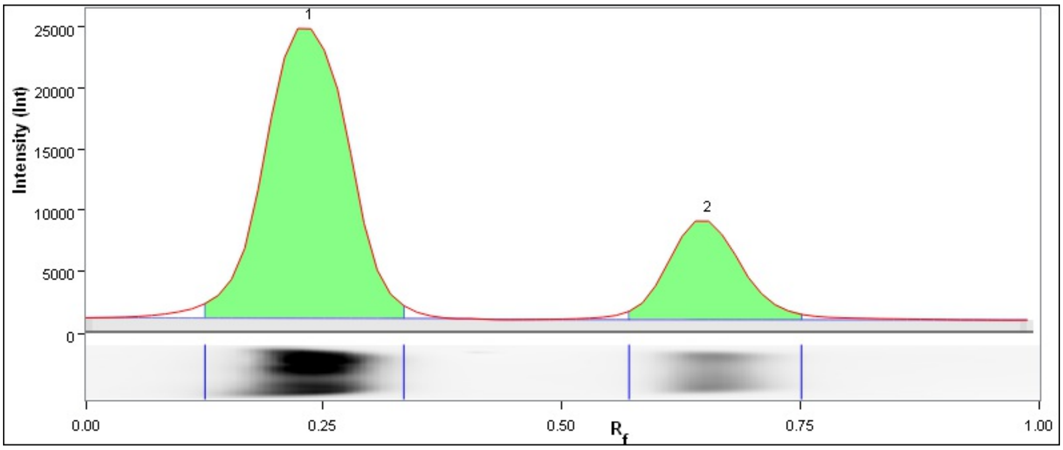

Band No.	Band Label	Mol. Wt. (KDa)	Relative Front	Adj. Volume (Int)	Volume (Int)	Abs. Quant.	Rel. Quant.	Band %	Lane %
1		N/A	0.250	10,677,230	11,753,921	N/A	N/A	77.1	74.9
2		N/A	0.667	3,177,622	3,952,292	N/A	N/A	22.9	22.3

Band Detection	Automatically detected bands with sensitivity: Low
Lane Background	Lane background subtracted with disk size: 0.1
Lane Width	5.00 mm

Lane 10

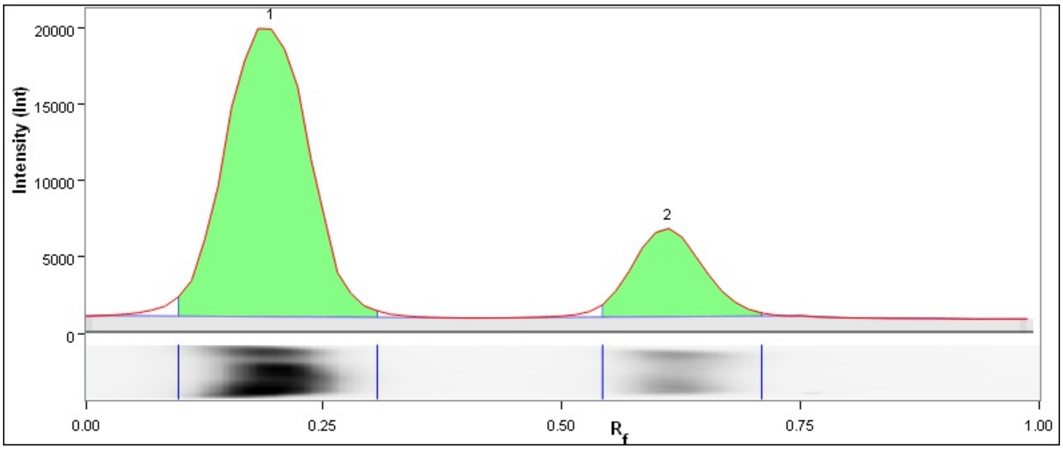

Band No.	Band Label	Mol. Wt. (KDa)	Relative Front	Adj. Volume (Int)	Volume (Int)	Abs. Quant.	Rel. Quant.	Band %	Lane %
1		N/A	0.208	9,867,927	10,977,009	N/A	N/A	79.6	77.6
2		N/A	0.625	2,524,315	3,371,201	N/A	N/A	20.4	19.8

Band Detection	Automatically detected bands with sensitivity: Low
Lane Background	Lane background subtracted with disk size: 0.1
Lane Width	5.00 mm

Lane 11

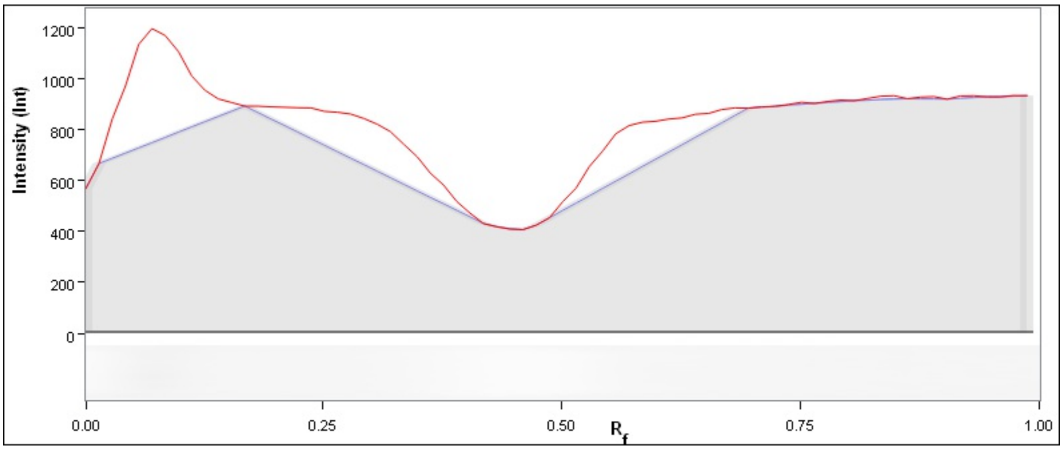

Band No.	Band Label	Mol. Wt. (KDa)	Relative Front	Adj. Volume (Int)	Volume (Int)	Abs. Quant.	Rel. Quant.	Band %	Lane %

Band Detection	Automatically detected bands with sensitivity: Low
Lane Background	Lane background subtracted with disk size: 0.1
Lane Width	5.00 mm

Lane 12

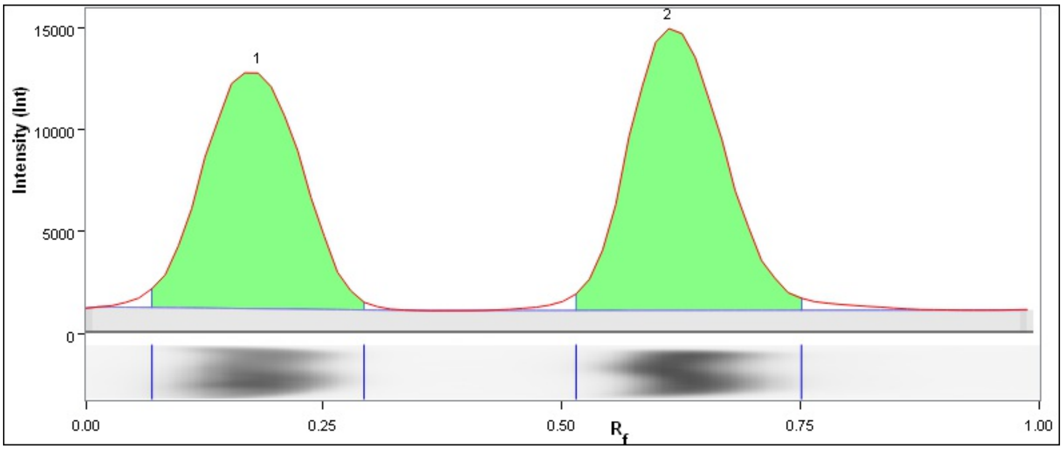

Band No.	Band Label	Mol. Wt. (KDa)	Relative Front	Adj. Volume (Int)	Volume (Int)	Abs. Quant.	Rel. Quant.	Band %	Lane %
1		N/A	0.194	6,468,878	7,698,497	N/A	N/A	46.4	45.2
2		N/A	0.625	7,466,745	8,613,292	N/A	N/A	53.6	52.2

Band Detection	Automatically detected bands with sensitivity: Low
Lane Background	Lane background subtracted with disk size: 0.1
Lane Width	5.00 mm
