# Supplementary material for: Development of Cellular Energy Metabolism During Differentiation of Human iPSCs into Cortical Neurons
Source: Mol Neurobiol. 2025 Nov 13;63(1):37. doi: 10.1007/s12035-025-05284-8 (PMC12615542; doi:10.1007/s12035-025-05284-8)
Supplement: Supplementary file 4 — Supplementary Material 4: Zipped folder containing uncropped Western blot images, quantification reports, and a descriptive summary file. (ZIP 4.70 MB) [file 12035_2025_5284_MOESM4_ESM.zip › Online Resource 4/Western blot ImageLab quantification reports/7057_c_quantification_report.pdf]

Image Report: 7057_SDHA_ATPaseBeta_core2_NDUFA9_quant2

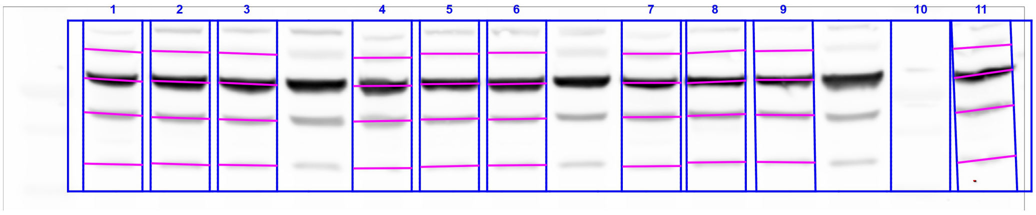

C:\Users\petr.pecina\Desktop\Neurodiferenciace projekt\Quant\reanalysis without D21\7057_SDHA_ATPaseBeta_core2_NDUFA9_quant2.scn

---

Acquisition Information

Program	2.1.12
Imager	LI-COR

Image Information

Acquisition Date	26/01/2023 9:56:08 AM
User Name	Kněžů Michal
Image Area (mm)	X: 86.3 Y: 17.5
Pixel Size (µm)	X: 84.7 Y: 84.7
Data Range (Int)	4 - 65535

Analysis Settings

Detection	Lane detection:<br>Manually created lanes<br><br>Band detection:<br>Automatically detected bands with sensitivity: High<br>Manually adjusted bands<br><br>Lane Background Subtraction:<br>Lane background subtracted with disk size: 0.1<br><br>Lane width: 5.00 mm

Lane Statistics

Lane No.	Adj. Total Band Vol. (Int)	Total Band Vol. (Int)	Adj. Total Lane Vol. (Int)	Total Lane Vol. (Int)	Bkgd. Vol. (Int)	Norm. Factor
1	12,244,860	12,933,744	12,666,474	14,191,270	1,524,796	N/A
2	16,248,482	16,868,454	17,190,712	18,547,299	1,356,587	N/A
3	15,017,270	15,421,656	15,930,059	16,908,102	978,043	N/A
4	13,854,321	14,553,884	14,346,558	15,696,596	1,350,038	N/A
5	13,975,566	14,360,187	14,800,327	15,755,124	954,797	N/A
6	14,588,694	15,032,433	15,409,620	16,426,072	1,016,452	N/A
7	13,567,345	14,289,151	13,966,303	15,527,266	1,560,963	N/A
8	14,501,669	15,131,848	15,091,846	16,436,633	1,344,787	N/A
9	12,884,302	13,252,344	13,371,406	14,291,511	920,105	N/A
10	N/A	N/A	640,268	1,434,290	794,022	N/A

11	14,284,608	15,044,764	14,833,367	16,415,747	1,582,380	N/A

Lane And Band Analysis

Lane 1

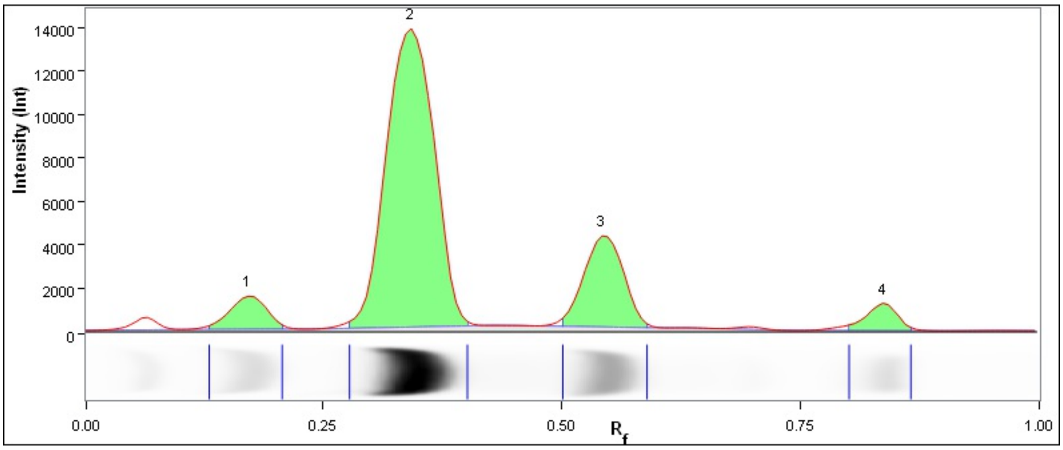

Band No.	Band Label	Mol. Wt. (KDa)	Relative Front	Adj. Volume (Int)	Volume (Int)	Abs. Quant.	Rel. Quant.	Band %	Lane %
1		N/A	0.176	751,365	856,503	N/A	N/A	6.1	5.9
2		N/A	0.347	8,778,846	9,094,909	N/A	N/A	71.7	69.3
3		N/A	0.547	2,199,815	2,427,791	N/A	N/A	18.0	17.4
4		N/A	0.841	514,834	554,541	N/A	N/A	4.2	4.1

Band Detection	Automatically detected bands with sensitivity: High
Lane Background	Lane background subtracted with disk size: 0.1
Lane Width	5.00 mm

Lane 2

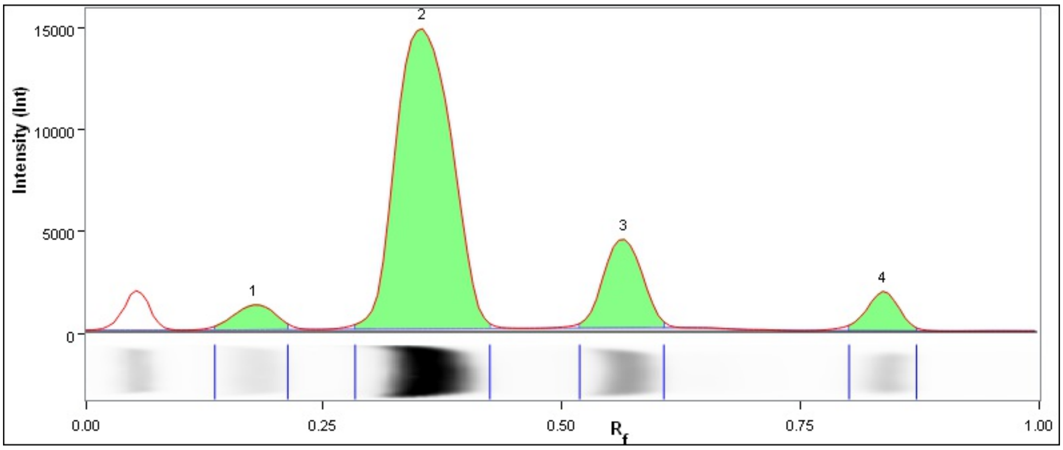

Band No.	Band Label	Mol. Wt. (KDa)	Relative Front	Adj. Volume (Int)	Volume (Int)	Abs. Quant.	Rel. Quant.	Band %	Lane %
1		N/A	0.182	745,347	845,706	N/A	N/A	4.6	4.3
2		N/A	0.359	12,100,546	12,358,317	N/A	N/A	74.5	70.4
3		N/A	0.571	2,496,644	2,713,882	N/A	N/A	15.4	14.5
4		N/A	0.841	905,945	950,549	N/A	N/A	5.6	5.3

Band Detection	Automatically detected bands with sensitivity: High
Lane Background	Lane background subtracted with disk size: 0.1
Lane Width	5.00 mm

Lane 3

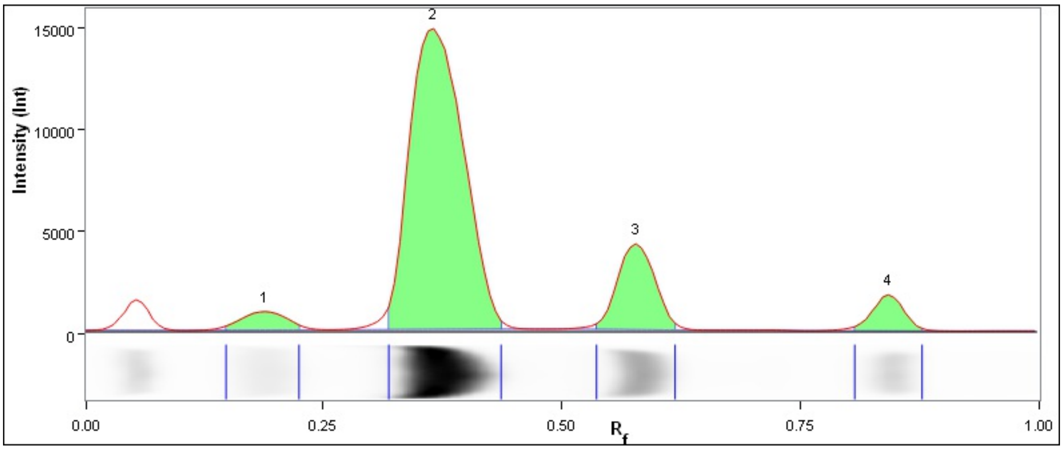

Band No.	Band Label	Mol. Wt. (KDa)	Relative Front	Adj. Volume (Int)	Volume (Int)	Abs. Quant.	Rel. Quant.	Band %	Lane %
1		N/A	0.194	581,032	662,806	N/A	N/A	3.9	3.6
2		N/A	0.371	11,399,095	11,572,732	N/A	N/A	75.9	71.6
3		N/A	0.582	2,247,723	2,357,463	N/A	N/A	15.0	14.1
4		N/A	0.847	789,420	828,655	N/A	N/A	5.3	5.0

Band Detection	Automatically detected bands with sensitivity: High
Lane Background	Lane background subtracted with disk size: 0.1
Lane Width	5.00 mm

Lane 4

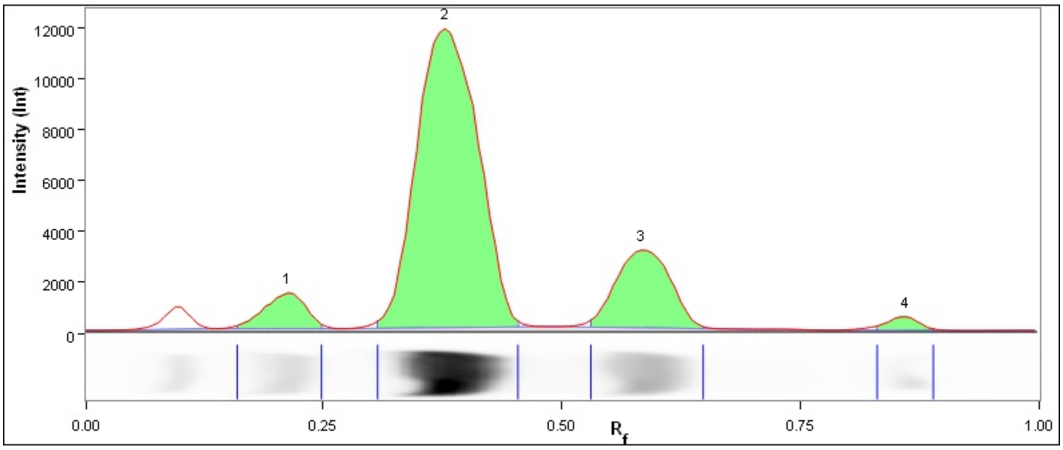

Band No.	Band Label	Mol. Wt. (KDa)	Relative Front	Adj. Volume (Int)	Volume (Int)	Abs. Quant.	Rel. Quant.	Band %	Lane %
1		N/A	0.218	836,502	974,326	N/A	N/A	6.0	5.8
2		N/A	0.382	10,395,800	10,697,349	N/A	N/A	75.0	72.5
3		N/A	0.588	2,383,600	2,604,437	N/A	N/A	17.2	16.6

4		N/A	0.865	238,419	277,772	N/A	N/A	1.7	1.7

Band Detection	Automatically detected bands with sensitivity: High
Lane Background	Lane background subtracted with disk size: 0.1
Lane Width	5.00 mm

Lane 5

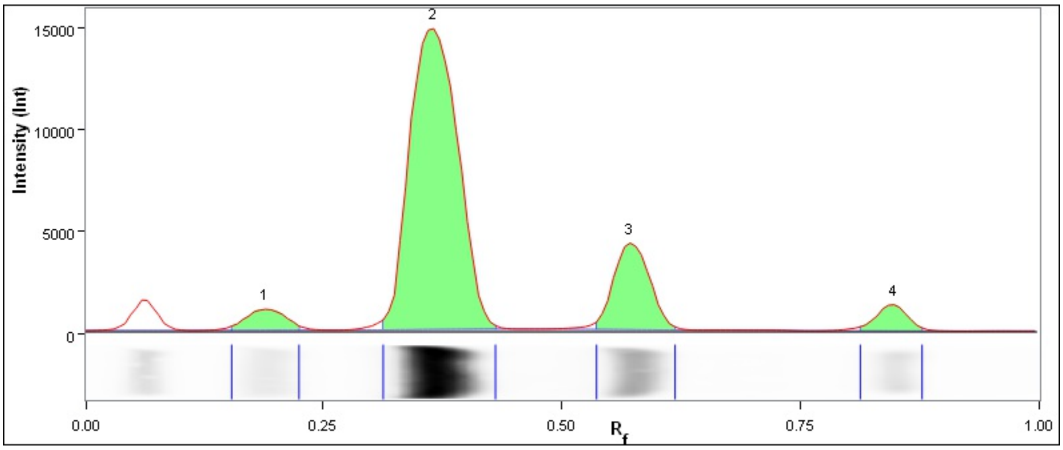

Band No.	Band Label	Mol. Wt. (KDa)	Relative Front	Adj. Volume (Int)	Volume (Int)	Abs. Quant.	Rel. Quant.	Band %	Lane %
1		N/A	0.194	602,449	671,007	N/A	N/A	4.3	4.1
2		N/A	0.371	10,545,365	10,715,934	N/A	N/A	75.5	71.3
3		N/A	0.576	2,240,643	2,350,383	N/A	N/A	16.0	15.1
4		N/A	0.853	587,109	622,863	N/A	N/A	4.2	4.0

Band Detection	Automatically detected bands with sensitivity: High
Lane Background	Lane background subtracted with disk size: 0.1
Lane Width	5.00 mm

Lane 6

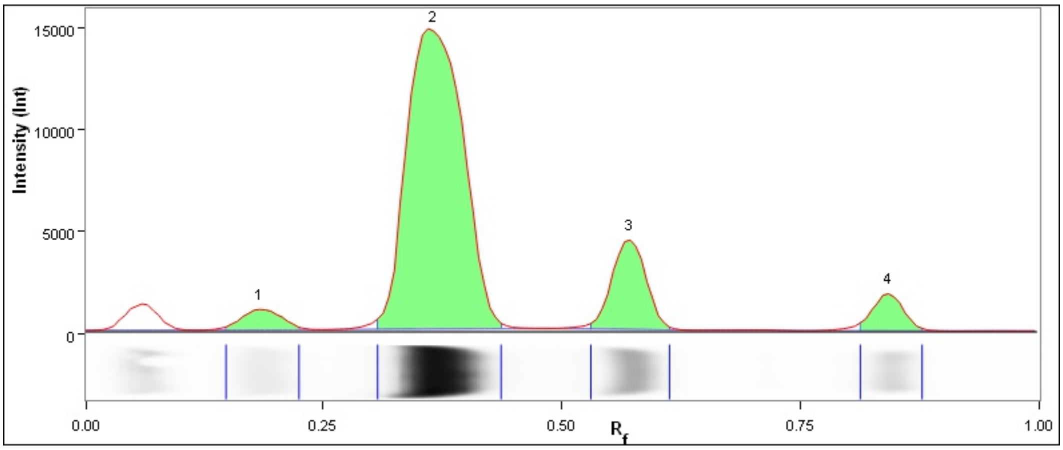

Band No.	Band Label	Mol. Wt. (KDa)	Relative Front	Adj. Volume (Int)	Volume (Int)	Abs. Quant.	Rel. Quant.	Band %	Lane %
1		N/A	0.188	543,095	628,114	N/A	N/A	3.7	3.5
2		N/A	0.371	11,280,328	11,491,076	N/A	N/A	77.3	73.2

3		N/A	0.576	2,022,107	2,134,915	N/A	N/A	13.9	13.1
4		N/A	0.847	743,164	778,328	N/A	N/A	5.1	4.8

Band Detection	Automatically detected bands with sensitivity: High
Lane Background	Lane background subtracted with disk size: 0.1
Lane Width	5.00 mm

Lane 7

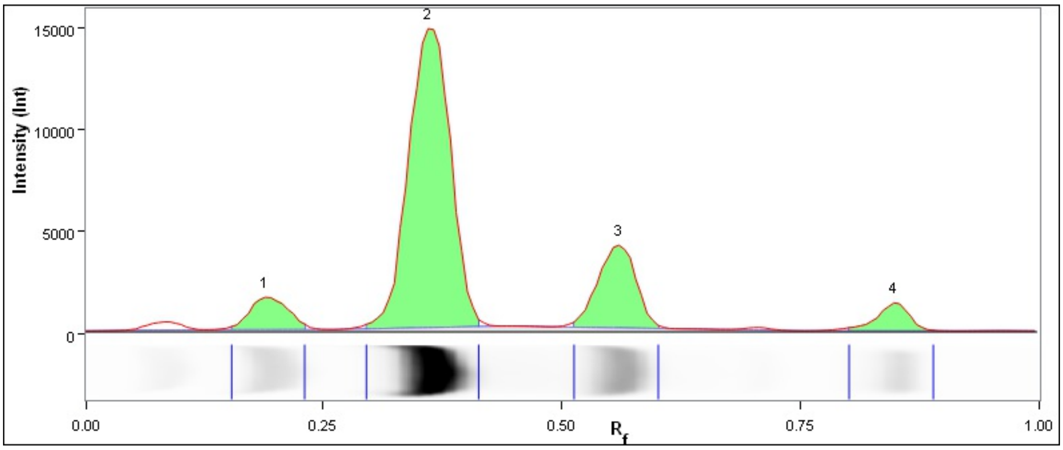

Band No.	Band Label	Mol. Wt. (KDa)	Relative Front	Adj. Volume (Int)	Volume (Int)	Abs. Quant.	Rel. Quant.	Band %	Lane %
1		N/A	0.194	943,646	1,055,982	N/A	N/A	7.0	6.8
2		N/A	0.365	9,561,776	9,886,630	N/A	N/A	70.5	68.5
3		N/A	0.565	2,375,635	2,605,145	N/A	N/A	17.5	17.0
4		N/A	0.853	686,288	741,394	N/A	N/A	5.1	4.9

Band Detection	Automatically detected bands with sensitivity: High
Lane Background	Lane background subtracted with disk size: 0.1
Lane Width	5.00 mm

Lane 8

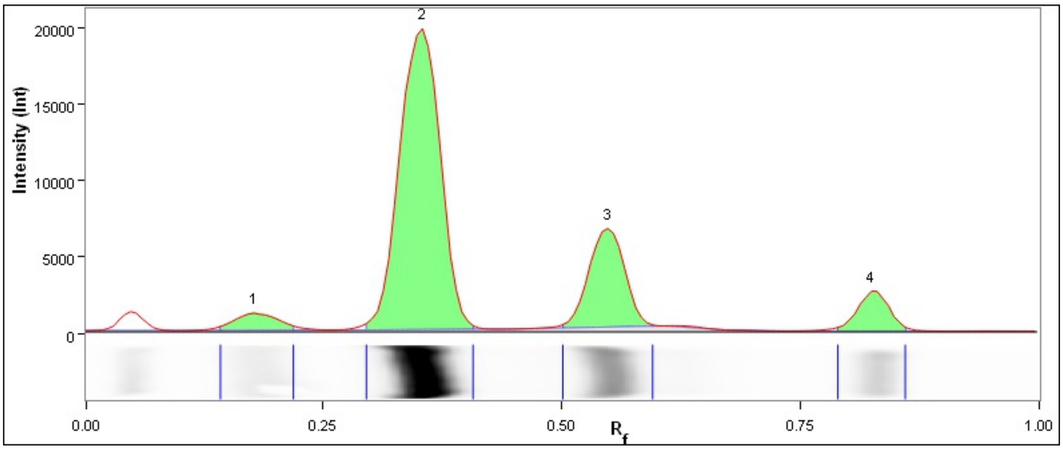

Band No.	Band Label	Mol. Wt. (KDa)	Relative Front	Adj. Volume (Int)	Volume (Int)	Abs. Quant.	Rel. Quant.	Band %	Lane %
1		N/A	0.182	605,340	686,701	N/A	N/A	4.2	4.0

2		N/A	0.359	9,991,001	10,172,013	N/A	N/A	68.9	66.2
3		N/A	0.553	2,877,843	3,207,889	N/A	N/A	19.8	19.1
4		N/A	0.829	1,027,485	1,065,245	N/A	N/A	7.1	6.8

Band Detection	Automatically detected bands with sensitivity: High
Lane Background	Lane background subtracted with disk size: 0.1
Lane Width	5.00 mm

Lane 9

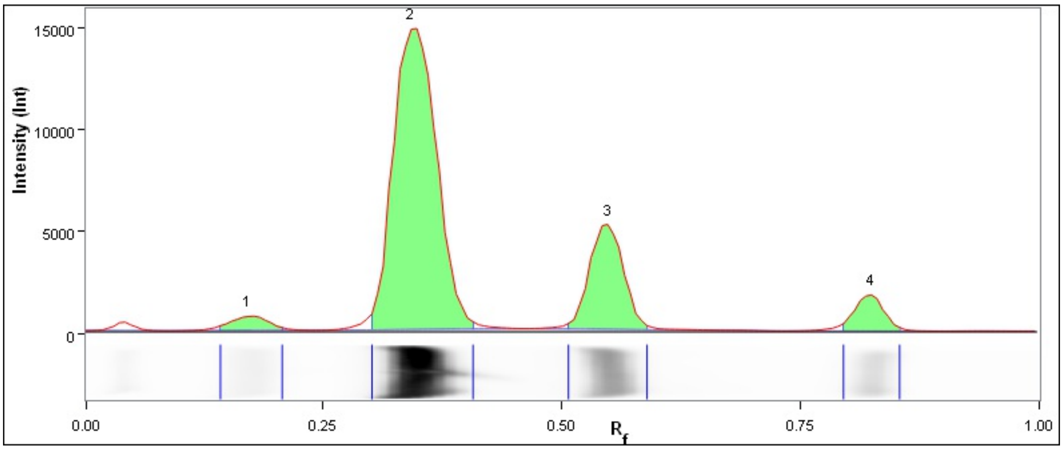

Band No.	Band Label	Mol. Wt. (KDa)	Relative Front	Adj. Volume (Int)	Volume (Int)	Abs. Quant.	Rel. Quant.	Band %	Lane %
1		N/A	0.176	370,461	425,862	N/A	N/A	2.9	2.8
2		N/A	0.347	9,249,607	9,408,317	N/A	N/A	71.8	69.2
3		N/A	0.553	2,527,914	2,652,699	N/A	N/A	19.6	18.9
4		N/A	0.829	736,320	765,466	N/A	N/A	5.7	5.5

Band Detection	Automatically detected bands with sensitivity: High
Lane Background	Lane background subtracted with disk size: 0.1
Lane Width	5.00 mm

Lane 10

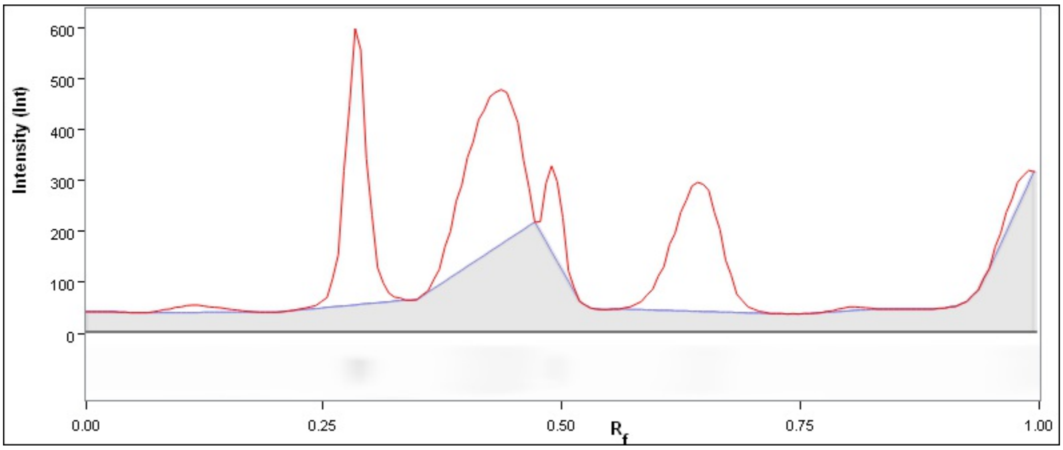

Band No.	Band Label	Mol. Wt. (KDa)	Relative Front	Adj. Volume (Int)	Volume (Int)	Abs. Quant.	Rel. Quant.	Band %	Lane %

Band Detection	Automatically detected bands with sensitivity: High
Lane Background	Lane background subtracted with disk size: 0.1
Lane Width	5.00 mm

Lane 11

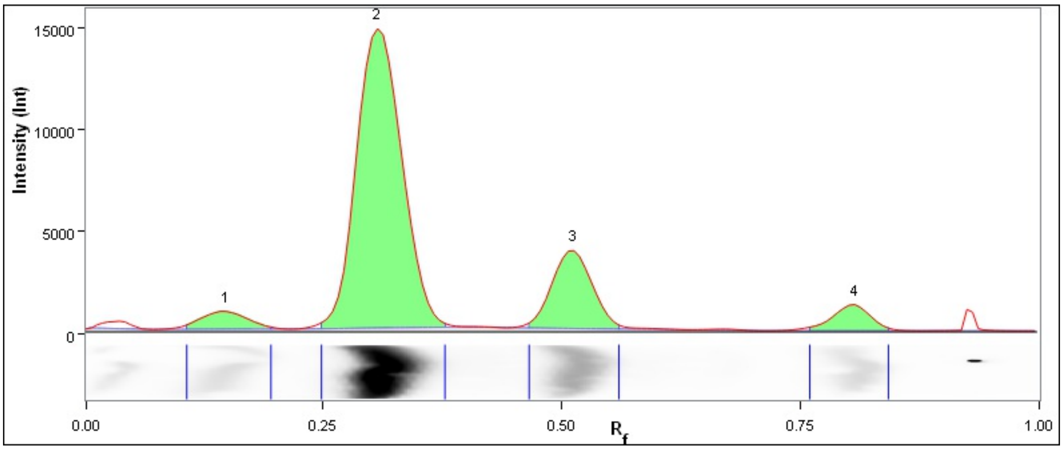

Band No.	Band Label	Mol. Wt. (KDa)	Relative Front	Adj. Volume (Int)	Volume (Int)	Abs. Quant.	Rel. Quant.	Band %	Lane %
1		N/A	0.153	629,530	785,113	N/A	N/A	4.4	4.2
2		N/A	0.312	10,494,979	10,835,586	N/A	N/A	73.5	70.8
3		N/A	0.518	2,408,734	2,616,178	N/A	N/A	16.9	16.2
4		N/A	0.812	751,365	807,887	N/A	N/A	5.3	5.1

Band Detection	Automatically detected bands with sensitivity: High
Lane Background	Lane background subtracted with disk size: 0.1
Lane Width	5.00 mm
