# Supplementary material for: Development of Cellular Energy Metabolism During Differentiation of Human iPSCs into Cortical Neurons
Source: Mol Neurobiol. 2025 Nov 13;63(1):37. doi: 10.1007/s12035-025-05284-8 (PMC12615542; doi:10.1007/s12035-025-05284-8)
Supplement: Supplementary file 4 — Supplementary Material 4: Zipped folder containing uncropped Western blot images, quantification reports, and a descriptive summary file. (ZIP 4.70 MB) [file 12035_2025_5284_MOESM4_ESM.zip › Online Resource 4/Western blot ImageLab quantification reports/7057_d_quantification_report.pdf]

Image Report: 7057_CS_hnRNPe1_quant2

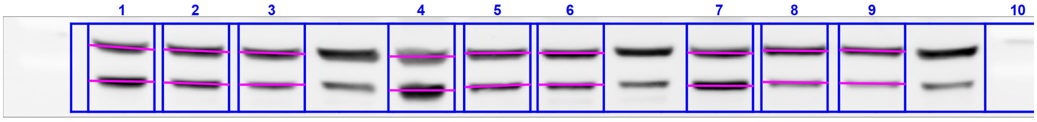

C:\Users\petr.pecina\Desktop\Neurodiferenciace projekt\Quant\reanalysis without D21\7057_CS_hnRNPe1_quant2.scn

Acquisition Information

Program	2.1.12
Imager	LI-COR

Image Information

Acquisition Date	28/03/2023 2:43:38 PM
User Name	Knězů Michal
Image Area (mm)	X: 85.7 Y: 7.5
Pixel Size (µm)	X: 84.7 Y: 84.7
Data Range (Int)	456 - 33793

Analysis Settings

Detection	Lane detection:<br>Manually created lanes<br><br>Band detection:<br>Automatically detected bands with sensitivity: Low<br>Manually adjusted bands<br><br>Lane Background Subtraction:<br>Lane background subtracted with disk size: 0.1<br><br>Lane width: 5.00 mm

Lane Statistics

Lane No.	Adj. Total Band Vol. (Int)	Total Band Vol. (Int)	Adj. Total Lane Vol. (Int)	Total Lane Vol. (Int)	Bkgd. Vol. (Int)	Norm. Factor
1	16,552,686	19,566,288	16,875,475	23,404,828	6,529,353	N/A
2	15,287,490	18,179,611	15,659,131	22,634,701	6,975,570	N/A
3	12,972,743	15,770,700	13,360,196	20,590,587	7,230,391	N/A
4	19,785,709	24,048,282	20,034,689	27,492,584	7,457,895	N/A
5	14,396,413	17,374,497	14,830,181	22,512,453	7,682,272	N/A
6	13,778,329	16,939,844	14,304,314	22,435,812	8,131,498	N/A
7	19,060,363	22,440,001	19,621,453	28,153,620	8,532,167	N/A
8	12,654,025	15,458,472	13,083,545	21,178,522	8,094,977	N/A
9	10,614,926	13,084,017	11,116,013	18,813,448	7,697,435	N/A
10	N/A	N/A	1,320,184	5,500,452	4,180,268	N/A
11	17,468,543	20,594,953	17,928,920	24,914,933	6,986,013	N/A

Lane And Band Analysis

Lane 1

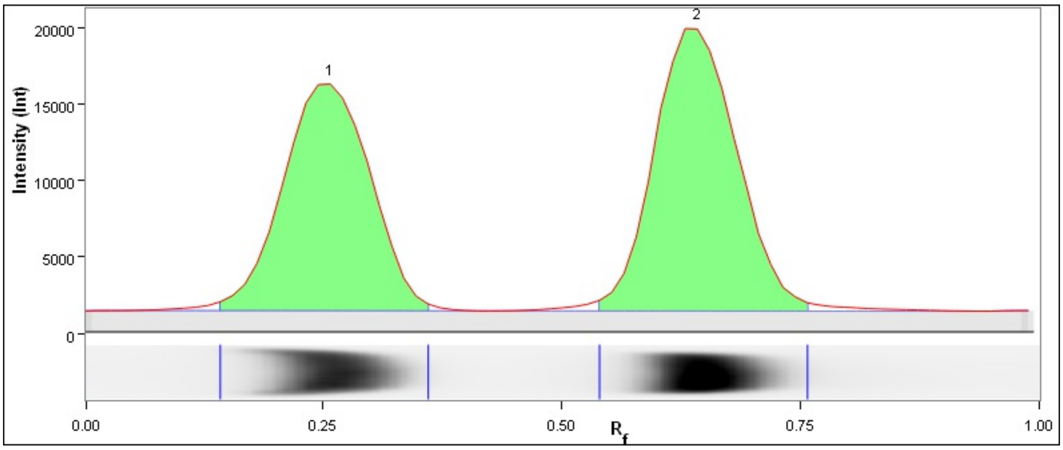

Band No.	Band Label	Mol. Wt. (KDa)	Relative Front	Adj. Volume (Int)	Volume (Int)	Abs. Quant.	Rel. Quant.	Band %	Lane %
1		N/A	0.269	7,601,088	9,115,559	N/A	N/A	45.9	45.0
2		N/A	0.654	8,951,598	10,450,729	N/A	N/A	54.1	53.0

Band Detection	Automatically detected bands with sensitivity: Low
Lane Background	Lane background subtracted with disk size: 0.1
Lane Width	5.00 mm

Lane 2

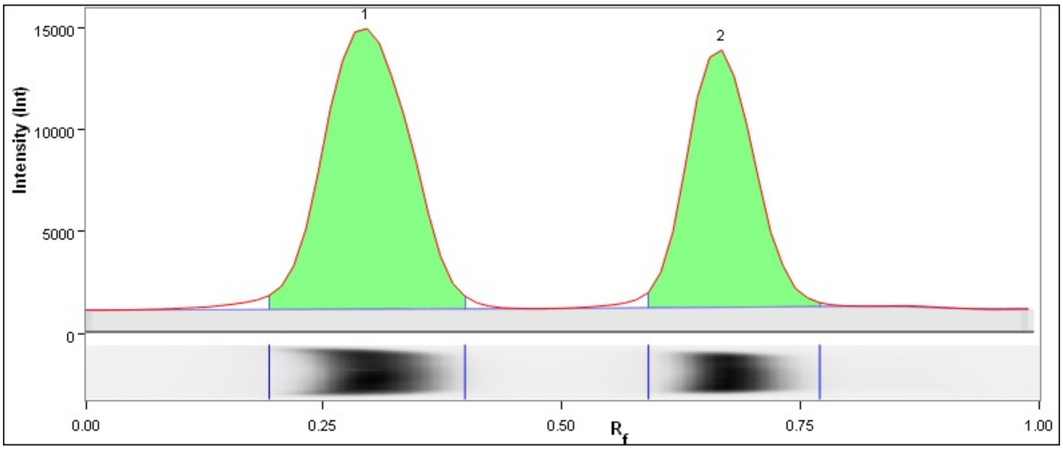

Band No.	Band Label	Mol. Wt. (KDa)	Relative Front	Adj. Volume (Int)	Volume (Int)	Abs. Quant.	Rel. Quant.	Band %	Lane %
1		N/A	0.308	8,917,083	10,391,139	N/A	N/A	58.3	56.9
2		N/A	0.679	6,370,407	7,788,472	N/A	N/A	41.7	40.7

Band Detection	Automatically detected bands with sensitivity: Low
Lane Background	Lane background subtracted with disk size: 0.1
Lane Width	5.00 mm

Lane 3

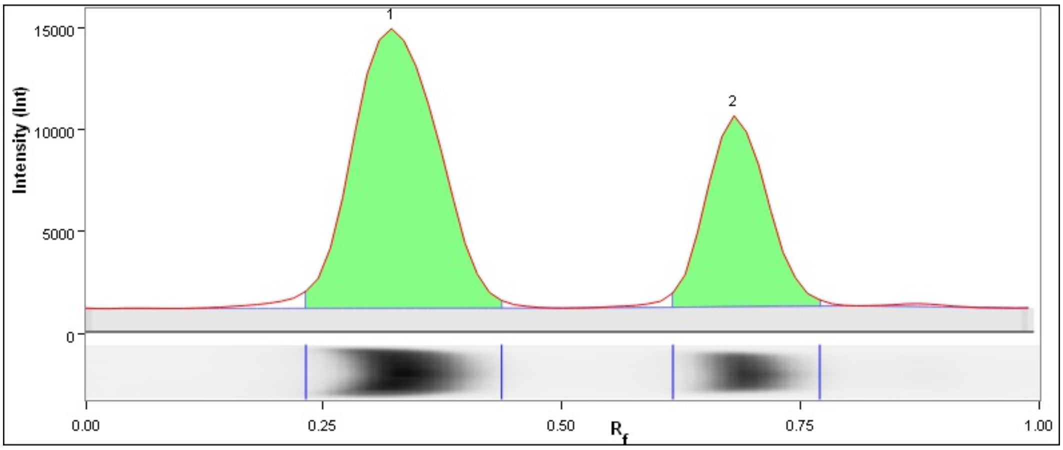

Band No.	Band Label	Mol. Wt. (KDa)	Relative Front	Adj. Volume (Int)	Volume (Int)	Abs. Quant.	Rel. Quant.	Band %	Lane %
1		N/A	0.333	8,713,415	10,250,896	N/A	N/A	67.2	65.2
2		N/A	0.692	4,259,328	5,519,804	N/A	N/A	32.8	31.9

Band Detection	Automatically detected bands with sensitivity: Low
Lane Background	Lane background subtracted with disk size: 0.1
Lane Width	5.00 mm

Lane 4

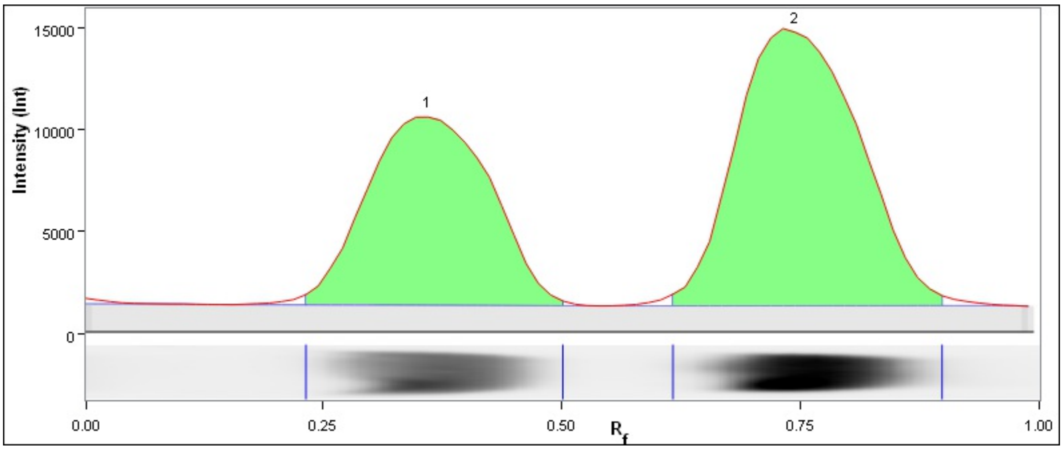

Band No.	Band Label	Mol. Wt. (KDa)	Relative Front	Adj. Volume (Int)	Volume (Int)	Abs. Quant.	Rel. Quant.	Band %	Lane %
1		N/A	0.372	8,055,329	10,155,611	N/A	N/A	40.7	40.2
2		N/A	0.756	11,730,380	13,892,671	N/A	N/A	59.3	58.6

Band Detection	Automatically detected bands with sensitivity: Low
Lane Background	Lane background subtracted with disk size: 0.1
Lane Width	5.00 mm

Lane 5

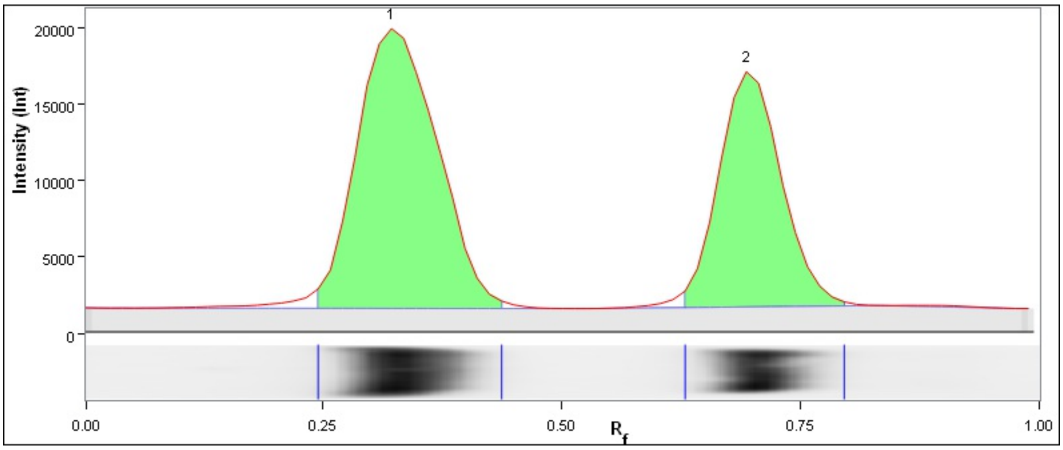

Band No.	Band Label	Mol. Wt. (KDa)	Relative Front	Adj. Volume (Int)	Volume (Int)	Abs. Quant.	Rel. Quant.	Band %	Lane %
1		N/A	0.333	8,707,692	10,240,276	N/A	N/A	60.5	58.7
2		N/A	0.705	5,688,721	7,134,221	N/A	N/A	39.5	38.4

Band Detection	Automatically detected bands with sensitivity: Low
Lane Background	Lane background subtracted with disk size: 0.1
Lane Width	5.00 mm

Lane 6

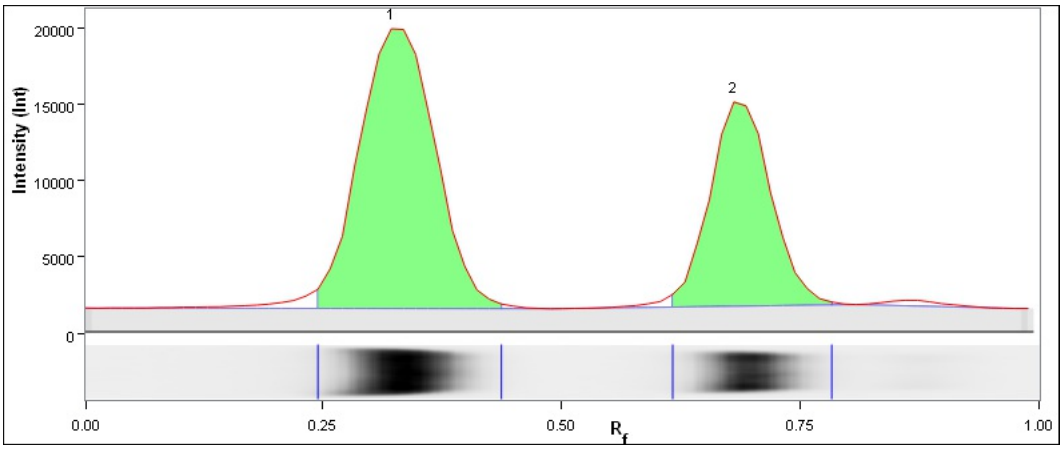

Band No.	Band Label	Mol. Wt. (KDa)	Relative Front	Adj. Volume (Int)	Volume (Int)	Abs. Quant.	Rel. Quant.	Band %	Lane %
1		N/A	0.333	8,681,791	10,280,514	N/A	N/A	63.0	60.7
2		N/A	0.692	5,096,538	6,659,330	N/A	N/A	37.0	35.6

Band Detection	Automatically detected bands with sensitivity: Low
Lane Background	Lane background subtracted with disk size: 0.1
Lane Width	5.00 mm

Lane 7

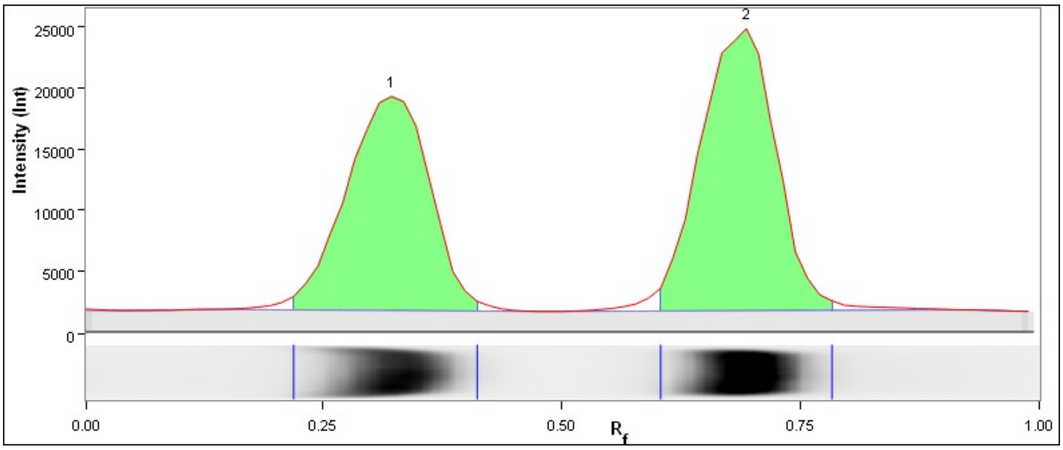

Band No.	Band Label	Mol. Wt. (KDa)	Relative Front	Adj. Volume (Int)	Volume (Int)	Abs. Quant.	Rel. Quant.	Band %	Lane %
1		N/A	0.333	8,721,616	10,469,727	N/A	N/A	45.8	44.4
2		N/A	0.705	10,338,747	11,970,274	N/A	N/A	54.2	52.7

Band Detection	Automatically detected bands with sensitivity: Low
Lane Background	Lane background subtracted with disk size: 0.1
Lane Width	5.00 mm

Lane 8

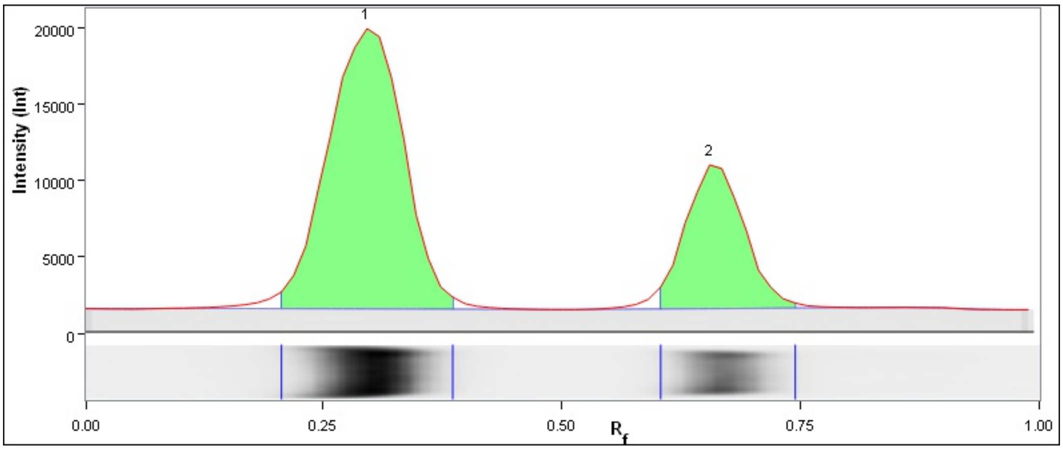

Band No.	Band Label	Mol. Wt. (KDa)	Relative Front	Adj. Volume (Int)	Volume (Int)	Abs. Quant.	Rel. Quant.	Band %	Lane %
1		N/A	0.308	9,080,513	10,623,658	N/A	N/A	71.8	69.4
2		N/A	0.667	3,573,512	4,834,814	N/A	N/A	28.2	27.3

Band Detection	Automatically detected bands with sensitivity: Low
Lane Background	Lane background subtracted with disk size: 0.1
Lane Width	5.00 mm

Lane 9

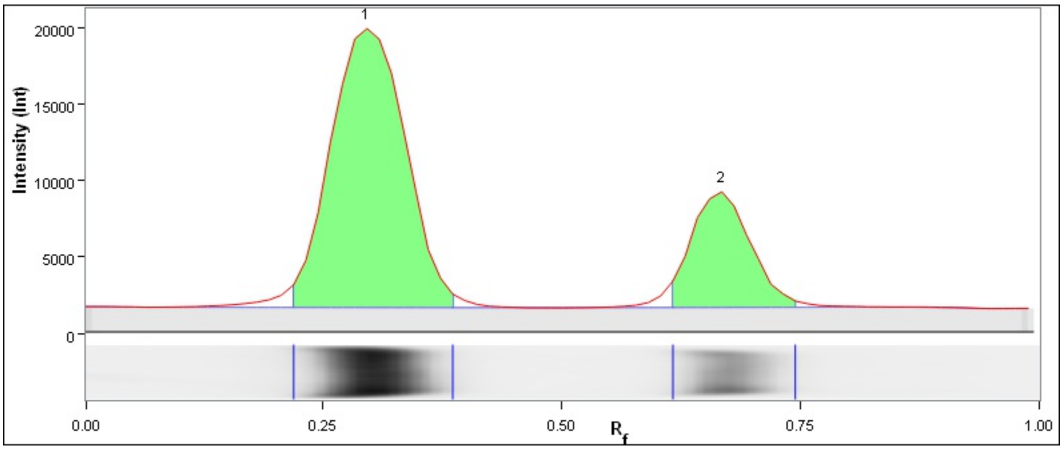

Band No.	Band Label	Mol. Wt. (KDa)	Relative Front	Adj. Volume (Int)	Volume (Int)	Abs. Quant.	Rel. Quant.	Band %	Lane %
1		N/A	0.308	8,040,520	9,418,642	N/A	N/A	75.7	72.3
2		N/A	0.679	2,574,406	3,665,375	N/A	N/A	24.3	23.2

Band Detection	Automatically detected bands with sensitivity: Low
Lane Background	Lane background subtracted with disk size: 0.1
Lane Width	5.00 mm

Lane 10

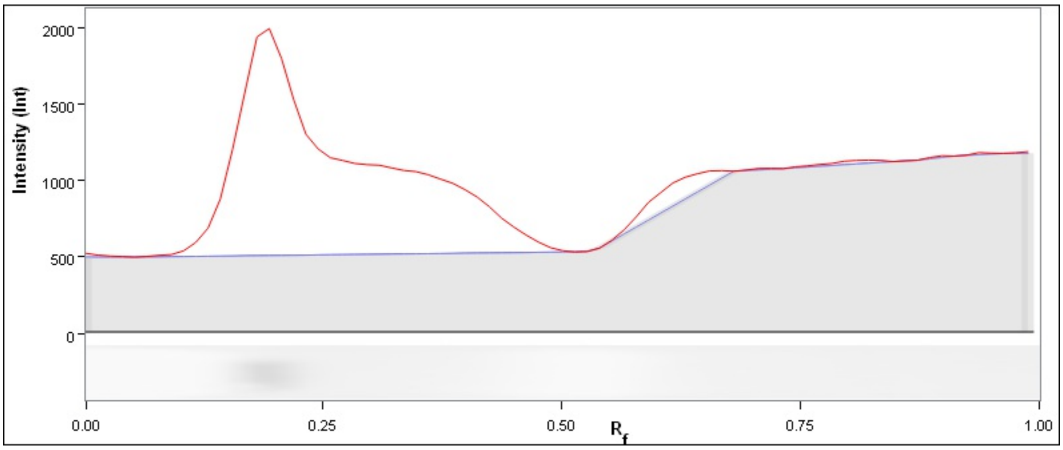

Band No.	Band Label	Mol. Wt. (KDa)	Relative Front	Adj. Volume (Int)	Volume (Int)	Abs. Quant.	Rel. Quant.	Band %	Lane %

Band Detection	Automatically detected bands with sensitivity: Low
Lane Background	Lane background subtracted with disk size: 0.1
Lane Width	5.00 mm

Lane 11

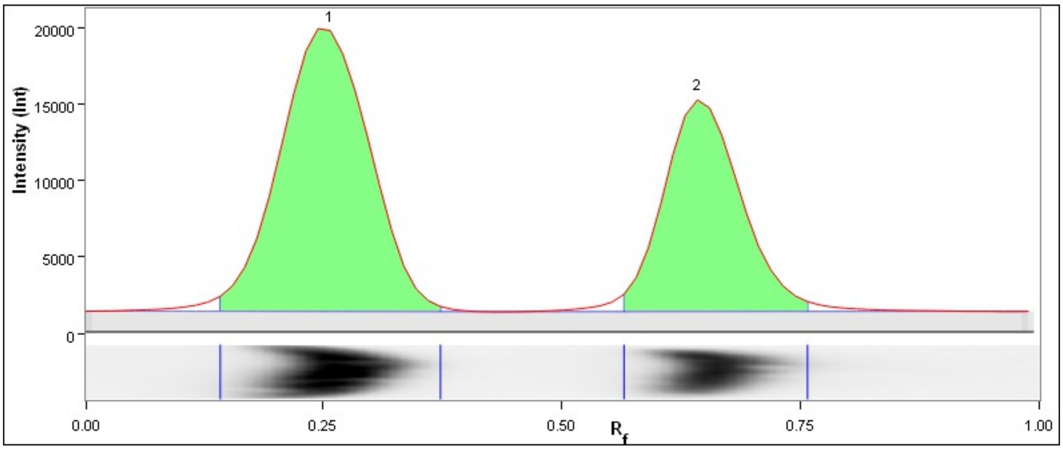

Band No.	Band Label	Mol. Wt. (KDa)	Relative Front	Adj. Volume (Int)	Volume (Int)	Abs. Quant.	Rel. Quant.	Band %	Lane %
1		N/A	0.269	10,639,234	12,337,431	N/A	N/A	60.9	59.3
2		N/A	0.654	6,829,309	8,257,522	N/A	N/A	39.1	38.1

Band Detection	Automatically detected bands with sensitivity: Low
Lane Background	Lane background subtracted with disk size: 0.1
Lane Width	5.00 mm
