# Supplementary figures and images for: Development of Cellular Energy Metabolism During Differentiation of Human iPSCs into Cortical Neurons
Source: Mol Neurobiol. 2025 Nov 13;63(1):37. doi: 10.1007/s12035-025-05284-8 (PMC12615542; doi:10.1007/s12035-025-05284-8)

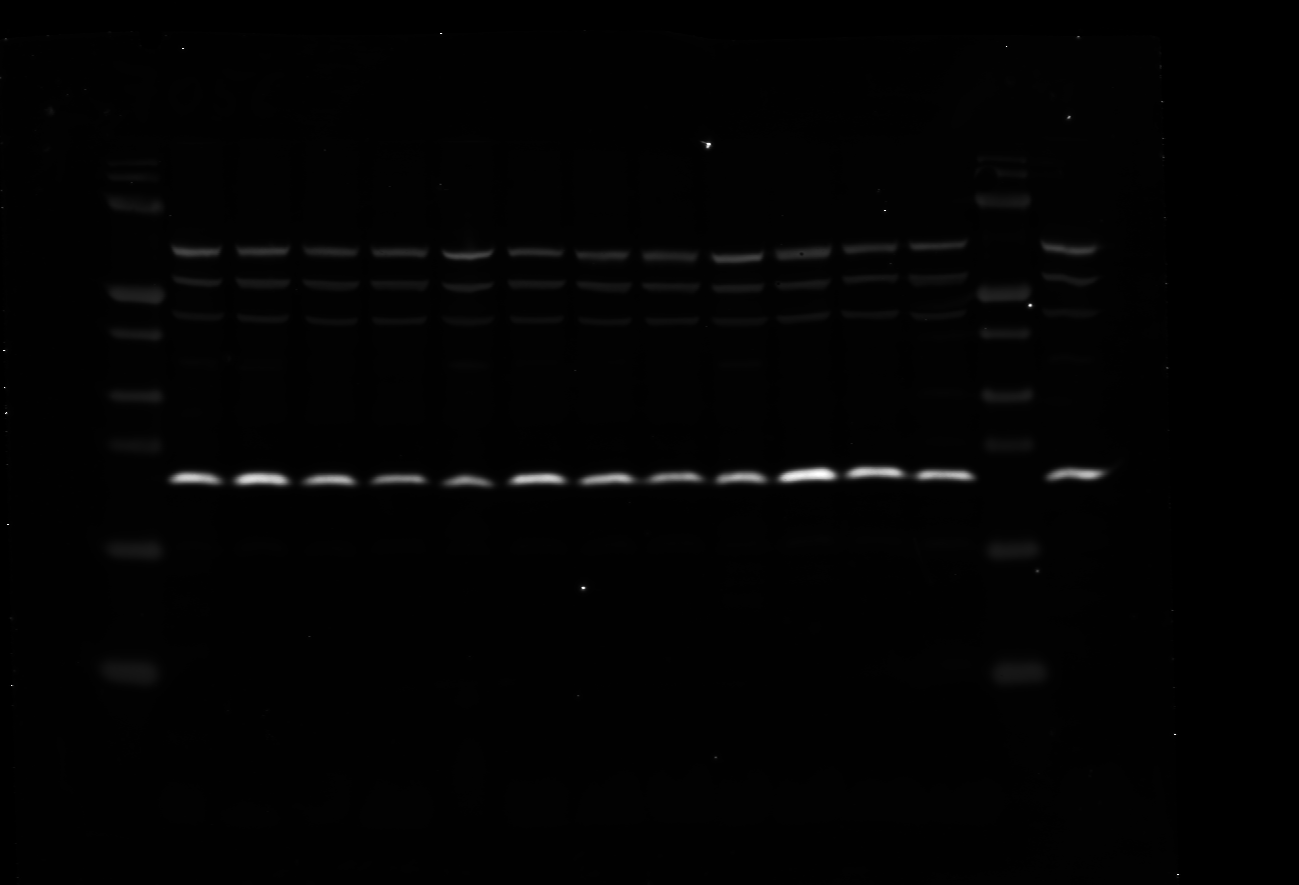

Supplement: Supplementary file 4 — Supplementary Material 4: Zipped folder containing uncropped Western blot images, quantification reports, and a descriptive summary file. (ZIP 4.70 MB) [file 12035_2025_5284_MOESM4_ESM.zip › Online Resource 4/Western blot raw image files/7056_a_image.tif]

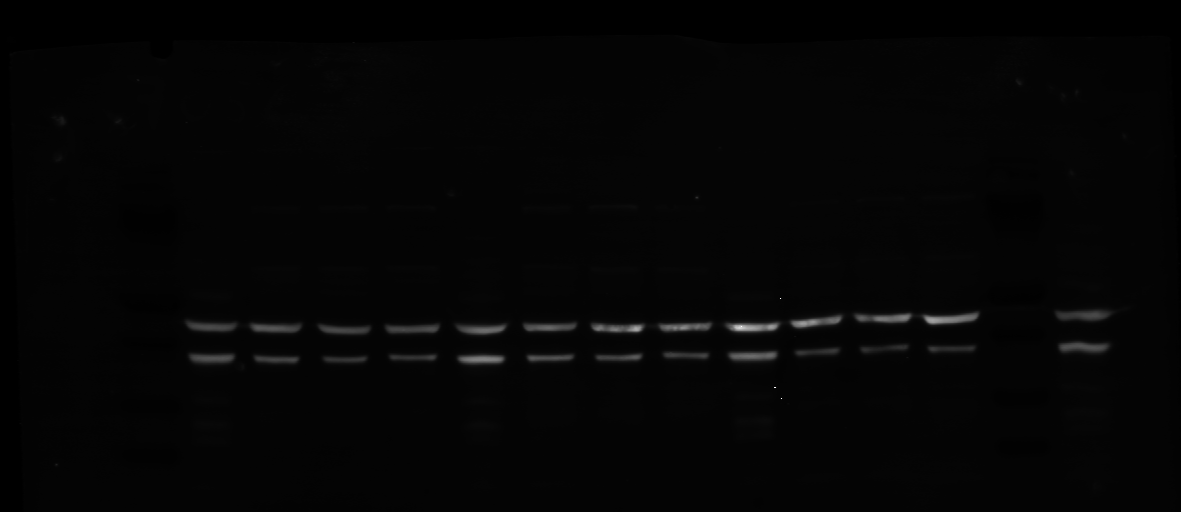

Supplement: Supplementary file 4 — Supplementary Material 4: Zipped folder containing uncropped Western blot images, quantification reports, and a descriptive summary file. (ZIP 4.70 MB) [file 12035_2025_5284_MOESM4_ESM.zip › Online Resource 4/Western blot raw image files/7056_b_image.tif]

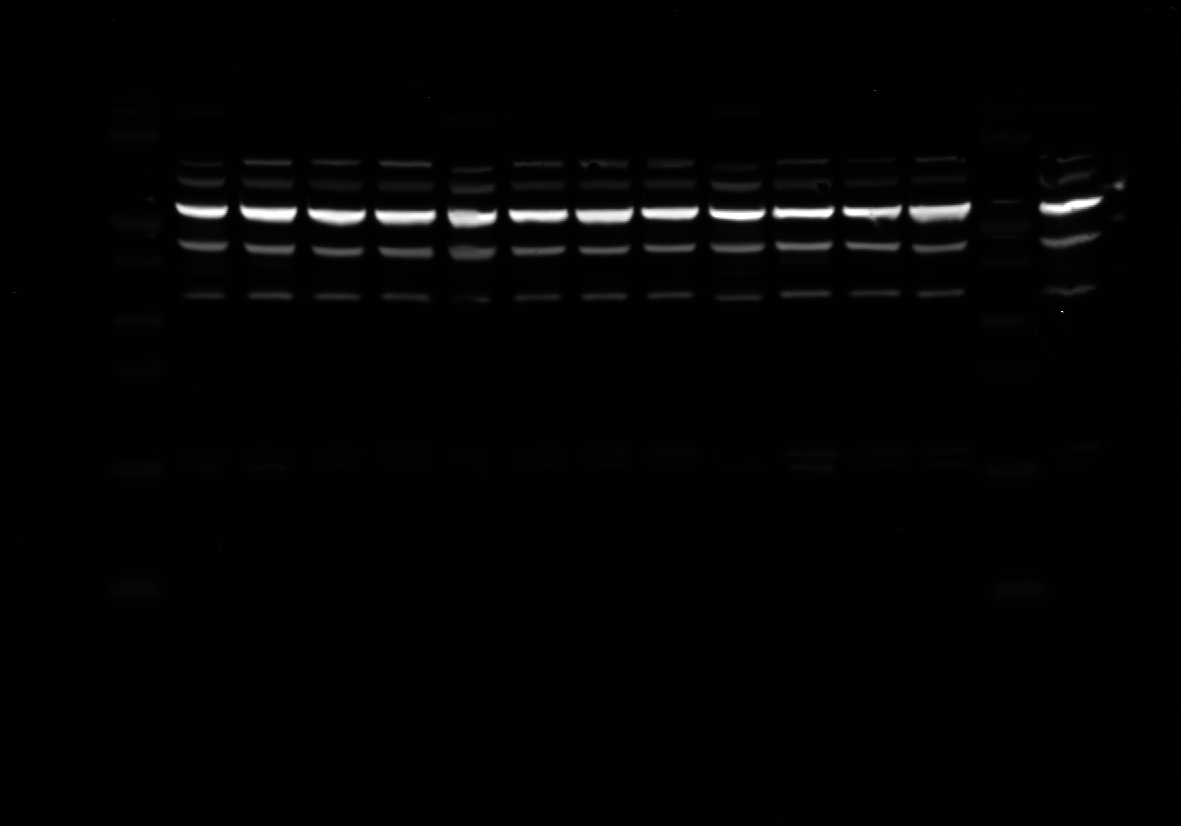

Supplement: Supplementary file 4 — Supplementary Material 4: Zipped folder containing uncropped Western blot images, quantification reports, and a descriptive summary file. (ZIP 4.70 MB) [file 12035_2025_5284_MOESM4_ESM.zip › Online Resource 4/Western blot raw image files/7057_c_image.tif]

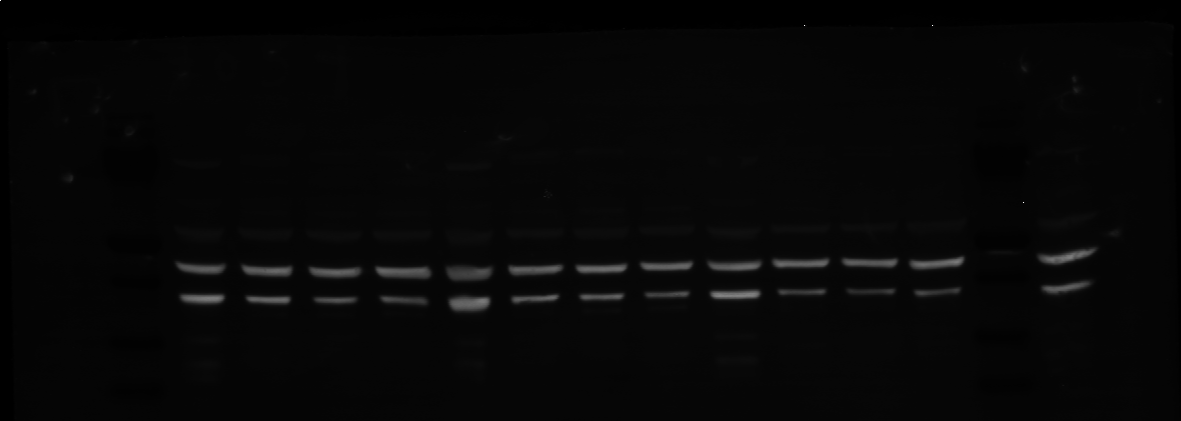

Supplement: Supplementary file 4 — Supplementary Material 4: Zipped folder containing uncropped Western blot images, quantification reports, and a descriptive summary file. (ZIP 4.70 MB) [file 12035_2025_5284_MOESM4_ESM.zip › Online Resource 4/Western blot raw image files/7057_d_image.tif]
